# Generation of Mixed Anhydrides via Oxidative Fragmentation of Tertiary Cyclopropanols with Phenyliodine(III) Dicarboxylates

**DOI:** 10.3390/molecules26010140

**Published:** 2020-12-30

**Authors:** Dzmitry M. Zubrytski, Gábor Zoltán Elek, Margus Lopp, Dzmitry G. Kananovich

**Affiliations:** 1 Department of Organic Chemistry, Belarusian State University, Leningradskaya 14, 220050 Minsk, Belarus; zubrytski_dzmitry@rambler.ru; 2 Department of Chemistry and Biotechnology, School of Science, Tallinn University of Technology, Akadeemia tee 15, 12618 Tallinn, Estonia; gabor.elek@taltech.ee (G.Z.E.); margus.lopp@taltech.ee (M.L.)

**Keywords:** cyclopropanols, mixed anhydrides, amides, macrolactonization, hypervalent iodine reagents, acylation, diversity-oriented synthesis

## Abstract

Oxidative fragmentation of tertiary cyclopropanols with phenyliodine(III) dicarboxylates in aprotic solvents (dichloromethane, chloroform, toluene) produces mixed anhydrides. The fragmentation reaction is especially facile with phenyliodine(III) reagents bearing electron-withdrawing carboxylate ligands (trifluoroacetyl, 2,4,6-trichlorobenzoyl, 3-nitrobenzoyl), and affords 95−98% yields of the corresponding mixed anhydride products. The latter can be straightforwardly applied for the acylation of various nitrogen, oxygen and sulfur-centered nucleophiles (primary and secondary amines, hydroxylamines, primary alcohols, phenols, thiols). Intramolecular acylation yielding macrocyclic lactones can also be performed. The developed transformation has bolstered the synthetic utility of cyclopropanols as pluripotent intermediates in diversity-oriented synthesis of bioactive natural products and their synthetic congeners. For example, it was successfully applied for the last-stage modification of a cyclic peptide to produce a precursor of a known histone deacetylase inhibitor.

## 1. Introduction

Currently, hydroxy-substituted cyclopropanes (cyclopropanols) are widely used in organic synthesis as versatile C_3_-building blocks [1,2,3,4,5], with a rapidly growing number of novel applications [6,7]. The remarkable progress in the field has been largely driven by the two following reasons. First, the hydroxy-substituted cyclopropanes are readily available compounds [8], which can be prepared by a number of high-yielding methods, including titanium-catalyzed cyclopropanation of carboxylic esters with Grignard reagents (the Kulinkovich reaction) [9,10], cyclopropanations with zinc carbenoids [11,12], 1,3-cyclization reactions [13,14] and via transformations of different cyclopropane precursors [15,16,17,18,19]. Second, the internal ring strain and the presence of electron-donating hydroxyl group facilitate ring-opening reactions of cyclopropanols under mild conditions, well tolerated by other functionalities. Typically, these reactions occur upon treatment with electrophilic reagents and proceed via homo- or heterolytic fission of the electron-rich carbon−carbon bonds adjacent to oxygen, affording β-functionalized ketones [20,21,22].

**Scheme 1:**
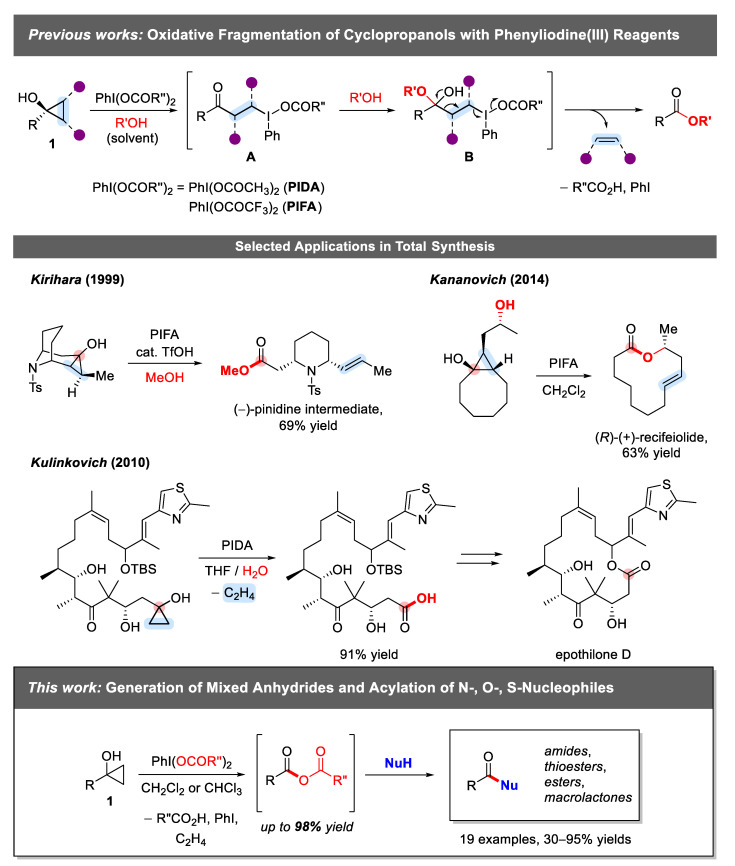
Oxidative fragmentation of tertiary cyclopropanols with phenyliodine(III) dicarboxylates and its applications in total synthesis. Outline of the current work.

However, in the reactions of cyclopropanols **1** with lead(IV) acetate [23,24] and phenyliodine(III) dicarboxylates [25,26] the initially produced β-functionalized ketone intermediates (e.g., **A**, Scheme 1) easily undergo Grob-type fragmentation, which is triggered by the nucleophilic attack of a solvent R′OH on the carbonyl group of **A**. The reaction cascade results in a fast (typically, within few minutes) oxidative fragmentation of **1** via the cleavage of both carbon−carbon bonds adjacent to oxygen and produces carboxylic acid (or ester) and alkene. Importantly, the fragmentation step requires *anti*-periplanar arrangement of the cleaved C−C and C−I bonds in intermediate **B** and occurs in a stereospecific fashion, with the stereochemistry of starting cyclopropanol fully preserved in the double bond of olefin product [24,25].

Oxidative ring scissoring of cyclopropanols with phenyliodine(III) diacetate (PIDA) or more reactive bis(trifluoroacetate) (PIFA) has been frequently utilized in total synthesis of natural products (selected examples are presented in Scheme 1). Intramolecular ring cleavage of bicyclic cyclopropanols is especially powerful since it allows installation of both ester function and a stereodefined double bond in a single preparative step [13,27]. For example, Kirihara and co-workers utilized oxidative fragmentation as the key step in the short synthesis of an alkaloid (−)-pinidine [28,29], while our group reported synthesis of natural macrolactone (+)-recifeiolide via the ring opening-macrolactonization cascade in an aprotic solvent [30]. On the other hand, cyclopropanol moiety can be considered as a convenient “protecting group” for carboxylate functionality, readily installed by Kulinkovich reaction with ethylmagnesium bromide [31,32,33,34,35,36]. The “deprotection” is performed by oxidative cleavage with PIDA and produces ethylene gas as the side product. The fragmentation reaction is generally high-yielding, compatible with densely functionalized substrates, and therefore can be rendered at the last steps, as Kulinkovich’s synthesis of epothilone D exemplifies [37,38].

Recently, we proposed the use of cyclopropanol moiety as pluripotent functional group for the diversity-oriented synthesis of bioactive compounds, which was validated by the efficient preparation of several histone deacetylase inhibitory cyclopeptides from the single cyclopropane-containing precursor [39]. The last-step ring opening in the latter required an expedient and high yielding protocol to generate a range of different carboxylic acid derivatives. Although the oxidation with hypervalent iodine reagents could provide access to esters and carboxylic acids, the similar route had not been previously applied for the preparation of other carboxylic acid derivatives, such as amides and hydroxamic acids, known as powerful histone deacetylase inhibitors [40,41,42,43]. Taking into account that amide bond is frequently occurs in pharmaceuticals [44,45] and therefore novel methods of amide synthesis currently attract a great deal of attention [46,47,48], one-pot preparation of amides from cyclopropanols was highly desired to bolster the performance of cyclopropanol moiety as pluripotent functionality suitable for the generation of bioactive molecular libraries.

In 2014, we noticed that oxidation of cyclopropanols with PIFA in CH_2_Cl_2_ affords mixed anhydrides of trifluoroacetic acid (TFA) as by-products [30], a plausible result of nucleophilic attack of TFA on the carbonyl group of **A** (Scheme 1), which occurs in the absence of large excess of a competitive nucleophile. Generation of mixed anhydride as intermediate in the oxidation reaction with PIDA in acetic acid was also evidenced by Momose and co-workers in 1995 [25]. However, the scope and limitations of the oxidative fragmentation leading to mixed anhydrides have remained unexamined and its synthetic potential has stayed untapped. We surmised that generation of mixed anhydrides as activated derivatives of carboxylic acids would offer a suitable solution to render the desired amide products upon the reaction with amines [49]. Here we report the general strategy for one-pot preparation of various carboxylic acid derivatives from cyclopropanols by oxidative fragmentation with phenyliodine(III) dicarboxylates via the intermediate formation of mixed anhydrides.

## 2. Results and Discussion

### 2.1. Generation of Mixed Anhydrides via Ring Scissoring of Tertiary Cyclopropanols with Phenyliodine(III) Dicarboxylates

**Scheme 2:**
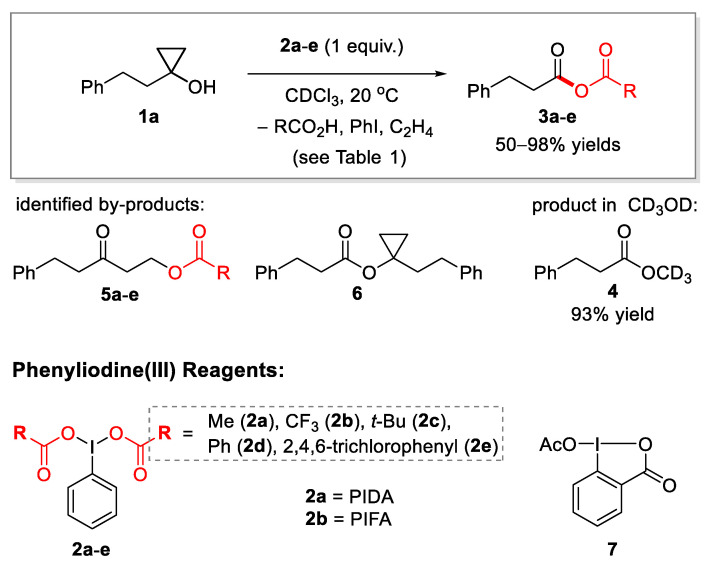
Generation of mixed anhydrides by oxidative fragmentation of cyclopropanol **1a.**

At the outset, the reactions of cyclopropanol **1a** (Scheme 2) with phenyliodine(III) reagents **2a**–**e**, bearing various carboxylate ligands, have been monitored by ^1^H-NMR spectroscopy (Table 1) in order to establish the best reaction conditions furnishing the desired mixed anhydride products **3** in high yields.

Oxidative fragmentation of **1a** with PIDA (**2a**) in a nucleophilic solvent (CD_3_OD) resulted in a rapid formation of the corresponding methyl ester **4** in 93% yield (Table 1, Entry 1). On the other hand, performing the same oxidation reaction in inert solvent (CDCl_3_) in the presence of benzylamine as external nucleophile, completely inhibited the oxidation of cyclopropanol **1a** and did not produce the corresponding *N*-benzylamide product (Entry 2). This result evidences that nucleophilic amines are incompatible with the oxidation step and therefore must be added only after its completion. Without the amine additive, the reaction between **1a** and PIDA in CDCl_3_ slowly generated the corresponding mixed anhydride **3a**, which was produced in 50% yield after 10 h reaction time (Entry 3). The structure of **3a** was confirmed by ^13^C-NMR and HMBC correlation spectra, showing two distinct signals of carbonyl groups in **3a** at δ_C_ 168.7 and 166.5 ppm (see the Supplementary Materials), while HRMS analysis of the crude reaction mixture revealed a mass peak with *m*/*z* = 215.0670, expected for a **3a**-derived molecular ion C_11_H_12_O_3_Na^+^. Although rather high (~90%) conversion of the starting materials was observed after 10 h reaction time, the modest 50% yield of anhydride **3a** was attained due to the generation of several oxidation side products, e.g., β-acyloxyketone **5a** (R = Me) [50].

As it was shown before by Momose and co-workers [51], catalytic amounts of triflic acid can greatly enhance the rate of oxidation, probably because of the generation of highly reactive iodonium triflates in situ [52,53,54] (Brønsted acid activation of carbonyl group in γ-keto iodonium intermediate **A** can also contribute to the rate acceleration effect). In our hands, addition of TfOH (1 mol%) tremendously accelerated the oxidation rate in CDCl_3_ (Entry 4). The reaction was completed less than in 5 min and yield of anhydride **3a** was also noticeably improved (up to 90%). The only side product was ester **6** (10% yield), a plausible outcome of acylation of **1a** with **3a** in the presence of TfOH catalyst. 

The use of more reactive PIFA reagent (**2b**) also resulted in fast (7 min) oxidation reaction, rendering the corresponding mixed TFA anhydride **3b** in high 95% yield with only trace amount of by-products (Entry 5). In contrast, the reaction with sterically hindered pivalate reagent **2c** was found to be extremely sluggish and produced a complex mixture of oxidation products after 24 h, even in the presence of TfOH catalyst (Entries 6 and 7).

Next, we attempted to generate mixed anhydrides of aromatic carboxylic acid, by using hypervalent iodine oxidants **2d** and **2e**, derivatives of benzoic and 2,4,6-trichlorobenzoic acids respectively. While the oxidation reaction with **2d** was rather slow and low-yielding (Entry 8), enhancing the electrophilic character of the phenyliodine(III) reagent by introducing more electron-deficient 2,4,6-trichlorobenzoate ligand was highly beneficial. Thus, the oxidative fragmentation of cyclopropanol **1a** with reagent **2e** was completed within 2 h (entry 9), producing the corresponding mixed anhydride in 98% yield and only trace amount of β-acyloxyketone side product **5e**. The structure of mixed anhydride **3e** was firmly confirmed by ^13^C-NMR and HMBC correlation spectra and supported by HRMS data (see the Supplementary Materials). The oxidation reaction can be further accelerated with Brønsted acid catalysts with the rate enhancement order TfOH>MsOH>TFA, corresponding to increasing acid strength (Entries 10–12). On the other hand, the Brønsted acid additives reduced yield of **3e** due to competitive acylation of cyclopropanol **1a**. Interestingly, replacement of CDCl_3_ solvent with toluene also notably accelerated the oxidation reaction (Entry 13 vs. 9), however resulted in slightly less clean reaction mixture (90% yield of **3e**). In contrast to dicarboxylates **2**, benziodoxole reagent **7** did not oxidize **1a** even in a nucleophilic solvent (methanol). Commonly, the reactions of benziodoxole-type reagents with cyclopropanols follow the radical mechanistic pathway [55,56] and require transition metal catalyst to trigger the ring opening [57,58,59].

As it can be clearly seen from the results presented in Table 1, the oxidation rate increases with an increase of electrophilic character of hypervalent iodine reagent **2** (e.g., **2d** vs. **2e**, Entries 8 and 9), which can be adjusted by altering the carboxylate substituents. To confirm that, we examined the reactivity difference of four phenyliodine(III) dicarboxylates, derivatives of benzoic (**2d**), 3-fluorobenzoic (**2f**), 3-nitrobenzoic (**2g**) and 2,4,6-trichlorobenzoic (**2e**) acids. These hypervalent iodine compounds were prepared in high yields from the corresponding benzoic acids and commercially available PIDA reagent via replacement of acetate ligands in the latter, performed in chlorobenzene [60,61] or diethyl carbonate as a sustainable replacement solvent (see the Experimental Section).

**Figure 1:**
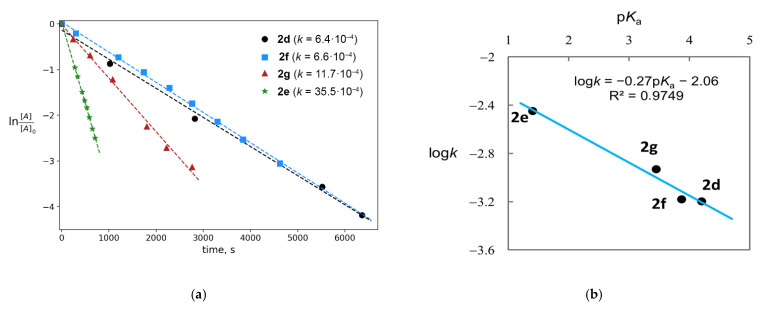
(**a**) Kinetic plot for the reaction of PhI(OCOAr)_2_ reagents **2d** (Ar = phenyl), **2f** (3-fluorophenyl), **2g** (3-nitrophenyl) and **2e** (2,4,6-trichlorophenyl) with excess of cyclopropanol **1a** (10 equiv.) in CDCl_3_ at 295 K and the corresponding pseudo-first-order rate constants. Initial concentration of **2** [*A*]_0_ = 0.02 M; (**b**) Relationship between the logarithm of rate constants and p*K*_a_ values of the corresponding substituted benzoic acids ArCO_2_H.

The oxidation reactions with PhI(OCOAr)_2_ reagents were carried out under the pseudo-first-order reaction conditions with excess of cyclopropanol **1a** (10 equiv., 0.2 M in CDCl_3_). Progress of the reactions was followed by ^1^H-NMR spectroscopy and pseudo-first-order rate constants have been determined (Figure 1a). As expected, the rate constants increased in order **2e** > **2g** > **2f** > **2d** following the increased electron-withdrawing character of the respective carboxylate ligands, a conclusion also supported by the corresponding linear free energy relationship (Figure 1b), showing a correlation between log*k* values and p*K*_a_ values of the corresponding benzoic acids [62], a parameter strongly dependent on phenyl ring electron density.

To summarize, electrophilic and reactive oxidants like PIFA (**2b**) or 2,4,6-trichlorobenzoic reagent **2e** are required for clean and fast generation of mixed anhydrides. The reactivity of phenyliodine(III) dicarboxylates in this reaction can be enhanced by installation of electron-deficient carboxylate ligands or by addition of strong Brønsted acid catalyst (e.g., TfOH).

### 2.2. Application of Mixed Anhydrides for Acylation of N-, O-, S-Nucleophiles and in Macrolactonizations

Mixed anhydrides contain two distinct reactive carbonyl groups, with the direction of nucleophilic attack and therefore selectivity of amide product formation governed by steric and/or electronic factors. Therefore, at the next stage, we evaluated suitability of several mixed anhydrides, produced by oxidation of cyclopropanol **1b**, for preparation of a model *N*-benzyl amide **8** (Table 2).

According to ^1^H-NMR monitoring, oxidation of cyclopropanol **1b** occurred faster than for **1a**. After completion of the oxidation step (TLC control) the reaction mixtures were quenched with excess (3 equiv.) of benzylamine. *N*-Benzylnonanamide (**8**) was isolated by column chromatography on silica gel (Table 2). As expected, the reaction with PIDA (**2a**, Entry 1) afforded low 22% yield of amide **8**, due to the lack of acylation selectivity (44% of *N*-benzylacetamide was formed) and formation of oxidation by-products (8-oxoundecyl acetate was isolated in 12% yield). On the other hand, mixed anhydrides of aromatic carboxylic acids, generated with reagents **2h**, **2e**, **2i** and **2g**, cleanly produced the required *N*-benzylamide **8** in high 88−95% yields (Entries 2−5). As in the case of cyclopropanol **1a** described above, the reaction rate increased with the increased electron-withdrawing character of a carboxylate ligand, with the fastest oxidation reaction (<14 min) observed for 2,4-dinitrobenzoyloxy reagent **2i** (Entry 4). On the contrary, oxidations with reagents **2k** and **2j** failed to render high yield of amide **8** due to the low solubility of these reagents in CH_2_Cl_2_ or chloroform and therefore slow oxidation rates (Entries 6 and 7). Reagents **2e** and **2g** were found to be the most suitable for practical applications, in terms of both amide product yields and the reaction times (Entries 3 and 5).

**Scheme 3:**
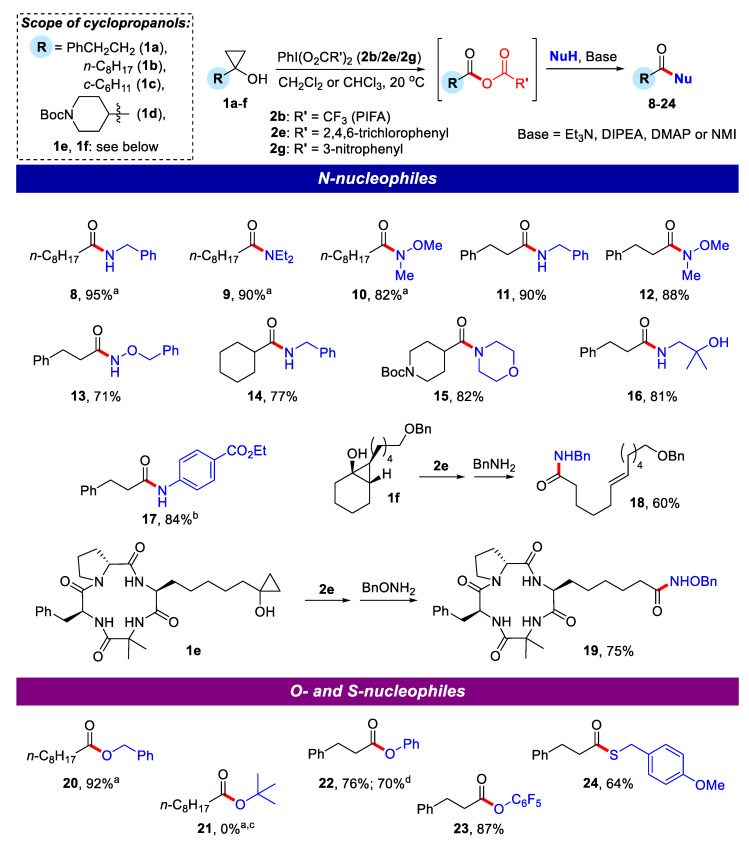
Acylation of *N*, *O* and *S*-nucleophiles with mixed anhydrides generated from cyclopropanols **1a**-**f** and phenyliodine(III) dicarboxylates. Unless noted otherwise, the reactions were run with 2,4,6-trichlorobenzoyloxy reagent **2e** in 0.5 mmol scale. Yields of isolated products are given (see Experimental Section for the details). NMR inspection of the several crude reaction mixtures evidenced quantitative formation of the corresponding amide products. Notes: ^a^ The reaction was performed with 3-nitrobenzoyloxy reagent **2g**. ^b^ With *N*-methylimidazole (NMI) as a base. ^c^ Nonanoic acid was obtained after aqueous work-up. ^d^ Oxidation was carried out with PIFA (**2b**), acylation of PhOH was performed in the absence of a base.

Having the most optimal conditions in hand, we performed one-pot synthesis of several amide products **8–19** (Scheme 3) by utilizing mixed anhydrides derived from reagents **2g**, **2e** and a number of cyclopropanols **1a**-**f**. After generation of the corresponding mixing anhydrides, the reaction mixtures were treated with excess (3 equiv.) of amine coupling partner (for BnNH_2_, Et_2_NH, morpholine) or with 1–2 equiv. of the corresponding amine in the presence of a base (3 equiv., Et_3_N, DIPEA or *N*-methylimidazole, see the Experimental Section). For the reactions with *O*-benzylhydroxylamine, a solution of mixed anhydride was added dropwise to excess of BnONH_2_ (10 equiv.) to avoid double acylation of the latter.

Following the general reaction protocol (see the Experimental Section), both primary *N*-benzyl amides **11**, **8** and secondary amide **9** were obtained in high 90−95% yields from cyclopropanols **1b** and **1a**. Weinreb amides **10**, **12** and *N*-benzyl hydroxamic acid **13** were also prepared in satisfactory yields from the same starting materials. The amidation reactions performed via generation of more sterically hindered mixed anhydrides, containing secondary alkyl substituents, were also successful, for both primary (**14**) and secondary (**15**) amide products. Hydroxy-substituted amide **16** was flawlessly prepared in 81% yield by the selective acylation of amino group in 1-amino-2-methylpropan-2-ol, since tertiary alcohols were found to be totally unreactive (see below). No column chromatography was required for the isolation of crystalline products **14–15** (see the Experimental Section). Aromatic and low-nucleophilic ethyl 4-aminobenzoate also afforded high 84% yield of amide **17** in the presence of *N*-methylimidazole (NMI) as a base [63]. Intramolecular oxidative fragmentation of bicyclic hydroxycyclopropane **1f** with reagent **2e** afforded amide with stereodefined *trans*-double bond **18** after quenching with benzylamine. The most important, a cyclopeptide scaffold tolerated the developed one-pot oxidation-amidation protocol, to produce *N*-benzyl-protected hydroxamic acid **19** in 75% yield. Compound **19** is known as immediate precursor of the corresponding hydroxamic acid Ky-2, a powerful histone deacetylase (HDAC) inhibitor and a potent anticancer agent [40,41,64]. This achievement illustrates the suitability of the developed protocol for the synthesis of bioactive amides and was previously employed by us, along with several other ring-opening reactions, to generate a number of HDAC inhibitors from the single cyclopropanol precursor according to diversity-oriented synthesis paradigm [39].

Besides preparation of amides, oxygen and sulfur-centered nucleophiles can also be employed to furnish the corresponding esters and thioester (Scheme 3). The two-step approach is advantageous for easily oxidizing nucleophilic partners (e.g., phenols, thiols), which are incompatible with hypervalent iodine compounds or are impractical to be used as solvents in a classical Momose reaction protocol. Thus, benzyl ester **20** was obtained in 92% yield by acylation of benzyl alcohol in the presence of Et_3_N. Acylation of phenol and pentafluorophenol can also be performed, to afford the corresponding ester products **22** and **23**. Ester **22** can also be prepared without a basic additive, upon the reaction of mixed TFA anhydride with phenol [65]. Thioester **24** was obtained in 64% yield by acylation of the corresponding thiol. The only limitation was the reaction with *tert*-butyl alcohol, which did not afford the expected ester **21**. This restriction, on the other hand, allowed to preform high-yielding synthesis of amide **16**. As it was shown by Momose and co-workers, preparation of esters from tertiary alcohols can be achieved in the presence of TfOH catalyst [51].

Synthesis of macrocyclic lactones from ω-hydroxycarboxylic (seco) acids represent one of the most prominent applications of mixed anhydrides in organic synthesis [66,67]. Thus, the venerable Yamaguchi esterification protocol relies on generation of mixed anhydrides with 2,4,6-trichlorobenzoic acid [68], while in the Shiina macrolactonizations the use of 4-trifluoromethylbenzoic [69] and 2-methyl-6-nitrobenzoic [70] anhydrides have been manifested. Importantly, macrolactonization of seco acid-derived anhydrides can also be promoted by Lewis acid catalysts [71,72,73,74] thus avoiding organic bases, what prompted us to investigate the direct conversion of ω-hydroxyalkyl substituted cyclopropanols **1g** and **1h** into the corresponding macrolactones **25a**,**b** upon treatment with phenyliodine(III) reagents **2e** and **2h** (Table 3).

At the outset (Entry 1), we examined scandium triflate-mediated intramolecular cyclization of the mixed anhydride, derived from cyclopropanol **1g** and 3-nitrobenzoic reagent **2g**, under the conditions developed by Yamamoto and co-workers [71]. Unfortunately, cyclopropanol ring opening product **27** was obtained instead of expected 11-membered macrolactone **25a**. To our delight, simple exchange of the solvent system from acetonitrile/THF to CH_2_Cl_2_/acetonitrile (Entry 2) allowed to perform the desired oxidative fragmentation-macrolactonization reaction. Addition of small amount of acetonitrile as co-solvent (ca. 1 mL per 150 mL of CH_2_Cl_2_) was only necessary to solubilize Sc(OTf)_3_ catalyst, which mediated intramolecular cyclization of intermediate 3-nitrobenzoic mixed anhydride [71] and furnished **25a** in 62% isolated yield, along with 14% of cyclic diolide side product **26a**. The macrocyclizations were performed with a diluted solution of **2g** or **2e** (ca. 0.005 M).

The analogous transformation performed with cyclopropanol **1h** afforded larger 13-membered macrocyclic lactone **25b** in 82% yield (Entry 3), although proceeded significantly slower and required 189 h reaction time, including slow 21 h addition of **1h** and subsequent refluxing of the reaction mixture for ca. 7 days. Aqueous work-up before the indicated time resulted in reduced yields of **25b** and delivered considerable amounts of 12-hydroxydodecanoic acid. The reaction outcome can be further improved with 2,4,6-trichlorobenzoic reagent **2e** (Entry 4) or with the use or hafnium(IV) triflate catalyst (Entry 5) [73,74].

**Scheme 4:**
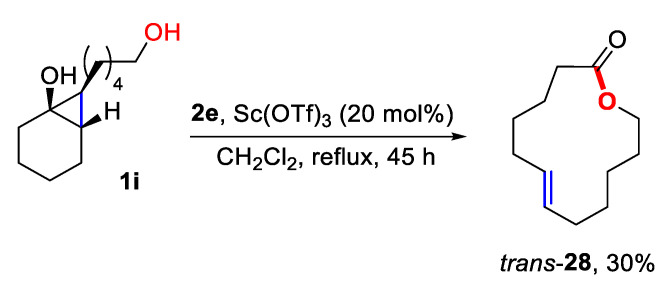
Synthesis of *trans*-alkenolide **28** via intramolecular ring scissoring-macrocyclization of bicyclic cyclopropanol **1i**.

Finally, we tested the performance Sc(OTf)_3_-catalyzed macrolactonization in tandem with intramolecular ring scissoring of bicyclic cyclopropanol **1i** with reagent **2e** (Scheme 4). Unfortunately in this case macrolactonization was less successful, affording the corresponding 13-membered macrocycle **28** with endocyclic *trans*-double bond in low 30% yield due to formation of several non-cyclic and diolide side-products. Nevertheless, yield of macrocycle **28** was almost twice higher than obtained previously by the reaction of **1i** with PIFA [30].

## 3. Experimental Section

### 3.1. General Experimental Methods

Solvents were used as obtained from commercial sources without any further purification or dried if required over 4 Å molecular sieves. Chemicals (including phenyliodine(III) reagents **2a**, **2b**) were purchased from Sigma-Aldrich (St. Louis, MO, USA), Fluorochem (London, UK) and Alfa Aesar (Ward Hill, Mass, USA) and used as received unless other indicated. Bis(*tert*-butylcarbonyloxy)iodobenzene **2c** was purchased from Santa Cruz Biotechnology (Dallas, TX, USA). Commercial samples of scandium and hafnium triflates were dehydrated prior to use by heating under reduced pressure (>1 torr). Cyclopropanols **1a**-**d** have been prepared by Kulinkovich cyclopropanation of the corresponding esters or lactones [9]. Preparation of cyclopropanols **1g** and **1h** is described in the Supplementary Materials. Peptide cyclopropanol **1e** and bicyclic cyclopropanols **1f** and **1i** have been prepared by following the published protocols [30,39]. Silica gel 40–100 μm was used for column chromatography; silica gel 60F_254_ plates were used for TLC. ^1^H-NMR (400 and 500 MHz), ^13^C-NMR (100.6 and 125 MHz) spectra were recorded on Avance spectrometers (Bruker, Billerica, Mass, USA). Chemical shifts are given in δ value with CHCl_3_ (δ = 7.26 ppm) and CDCl_3_ (δ = 77.16 ppm) as internal standards for ^1^H-NMR and ^13^C-NMR spectra. FT-IR spectra were recorded on a Bruker Tensor 27 FT or a Bruker Vertex 70 spectrometers. HRMS data was obtained on a HPLC/Q-TOF G6540A Mass Spectrometer (Agilent, Santa Clara, CA, USA) using AJS ESI method in positive ion detection modes or a LTQ Orbitrap Discovery spectrometer (Thermo Fisher Scientific, Waltham, Mass, USA) using electrospray ionization (ESI). Melting points were determined with a SMP40 apparatus (Stuart, Staffordshire, UK).

### 3.2. Typical Procedure for Preparation of PhI(O_2_CAr)_2_ Reagents [for Ar = 2,4,6-trichlorophenyl, ***2e***]

A 250 mL round-bottom flask was charged with (diacetoxyiodo)benzene (3.22 g, 10 mmol) and 2,4,6-trichlorobenzoic acid (4,51 g, 20 mmol). Anhydrous diethyl carbonate (50 mL) was added and the solids were dissolved via stirring and gentle heating with a heat gun. The obtained clear solution was stirred at room temperature for 2 h (during this stage, white precipitate could start to form). The solvent was evaporated under reduced pressure. The remained solid or oily residue was triturated with cyclohexane, filtered, washed with cyclohexane (2 × 10 mL) and dried under reduced pressure to afford the title compound (6.19 g, 95% yield) as white or slightly yellowish crystals. The obtained material is pure enough to be used as obtained. If required, purification can be performed by recrystallization from ethyl acetate/cyclohexane. (Bis(2,4,6-trichlorobenzoyloxy)iodo)benzene **2e**: white crystalline solid, m.p. 159 °C (ethyl acetate/cyclohexane). IR (KBr), cm^−1^ 1659, 1580, 1572, 1120, 557. ^1^H-NMR (400 MHz, CDCl_3_) *δ* = 8.26 (d, *J* = 7.6 Hz, 2H), 7.63 (t, *J* = 7.6 Hz, 1H), 7.52 (t, *J* = 7.6 Hz, 2H), 7.27 (s, 4H). ^13^C-NMR (100.6 MHz, CDCl_3_) *δ* = 168.2, 135.7, 135.6, 132.7, 132.4, 131.4, 128.1, 123.0. HRMS (ESI) *m/z* calcd for C_20_H_9_Cl_6_IO_4_Na^+^ [M+Na]^+^ 672.7569 (monoisotopic), 674.7541 (most abundant), found [M+Na]^+^ 672.7555 (monoisotopic), 674.7531 (most abundant).

*(Bis(3-nitrobenzoyloxy)iodo)benzene* (**2g**): white crystalline solid, m.p. 150–151 °C (dichloromethane/petroleum ether). IR (KBr), cm^−1^ 1657, 1625, 1615, 1524, 1348, 721, 430. ^1^H-NMR (400 MHz, CDCl_3_) *δ* = 8.71 (t, *J* = 1.9 Hz, 2H), 8.36 (ddd, *J* = 8.3, 1.9, 1.1 Hz, 2H), 8.32–8.20 (m, 4H), 7.78–7.67 (m, 1H), 7.66–7.56 (m, 4H). ^13^C-NMR (100.6 MHz, CDCl_3_) *δ* = 169.1, 148.2, 136.0, 135.2, 132.6, 131.8, 131.6, 129.6, 127.2, 125.2, 122.3.

*(Bis(benzoyloxy)iodo)benzene* (**2d**): white crystalline solid. ^1^H-NMR (400 MHz, CDCl_3_) *δ* = 8.27–8.21 (m, 2H), 7.96–7.90 (m, 4H), 7.66–7.60 (m, 1H), 7.59–7.46 (m, 4H), 7.41–7.34 (m, 4H). ^13^C-NMR (100.6 MHz, CDCl_3_) *δ* = 171.5, 135.0, 132.6, 131.8, 131.1, 130.3, 130.2, 128.3, 122.5. NMR data correspond to those published previously [75].

*(Bis(3-fluorobenzoyloxy)iodo)benzene* (**2f**): white crystalline solid. ^1^H-NMR (400 MHz, CDCl_3_) *δ* = 8.27–8.20 (m, 2H), 7.74–7.70 (m, 2H), 7.69–7.62 (m, 1H), 7.62–7.51 (m, 4H), 7.35 (td, J = 8.0, 5.5 Hz, 2H), 7.19 (tdd, J = 8.0, 2.7, 1.1 Hz, 2H). ^13^C-NMR (100.6 MHz, CDCl_3_) *δ* = 170.2 (d, *J*_CF_ = 2.8 Hz), 162.5 (d, *J*_CF_ = 246.9 Hz), 135.0, 132.4 (d, *J*_CF_ = 7.2 Hz), 132.1, 131.3, 129.9 (d, *J*_CF_ = 7.7 Hz), 126.0 (d, *J*_CF_ = 3.0 Hz), 122.4, 119.7 (d, *J*_CF_ = 21.4 Hz), 117.1 (d, *J*_CF_ = 22.7 Hz).

Other phenyliodine(III) dicarboxylates **2h**-**k** were prepared analogously in chlorobenzene as solvent on a 1–2 mmol scale following published procedures [60,61], and used as obtained without any purification.

### 3.3. General Procedure for Generation of Mixed Anhydrides from Cyclopropanols and Reagent ***2e***

A solution of cyclopropanol **1** (0.5 mmol) in anhydrous CHCl_3_ or CH_2_Cl_2_ (3 mL) was added to the stirred solution of phenyliodine(III) reagent **2e** (327 mg, 0.5 mmol, 1.0 equiv.) in anhydrous CHCl_3_ or CH_2_Cl_2_ (3 mL). The reaction mixture was stirred at room temperature. TLC analysis or ^1^H-NMR monitoring (in CDCl_3_) showed full consumption of the starting cyclopropanol **1** after 2 h. The reaction with reagent **2g** was carried out in the same manner. The obtained solution of a mixed anhydride was quenched with a corresponding nucleophile as described below, affording compounds **8**–**24**.

#### 3.3.1. N-Benzylnonanamide (**8**)

Benzylamine (160 mg, 1.5 mmol, 3 equiv.) was added to the solution of mixed anhydride, generated from 1-octylcyclopropan-1-ol **1b** and reagent **2g** in CH_2_Cl_2_. Stirring was continued overnight (12 h). The reaction was quenched with 10% HCl solution, then extracted with CH_2_Cl_2_ (3 × 5 mL). The combined organic layers were dried (MgSO_4_) and filtered. After evaporation of the solvent, the product was isolated by silica gel column chromatography (PE/EtOAc). Colorless oil (117 mg, 95% yield). IR (CCl_4_) cm^−1^ 3453, 3331, 3089, 3068, 3032, 1682, 1532, 1505. ^1^H-NMR (400 MHz, CDCl_3_) *δ* = 7.38–7.22 (m, 5H), 5.69 (br.s, 1H), 4.45 (d, *J* = 5.6 Hz, 2H), 2.21 (t, *J* = 7.5 Hz, 2H), 1.66 (quint., *J* = 7.5 Hz, 2H), 1.38–1.18 (m, 10H), 0.88 (t, *J* = 6.8 Hz, 3H). ^13^C-NMR (100.6 MHz, CDCl_3_) *δ* = 173.5, 138.5, 128.7, 127.8, 127.5, 43.7, 36.8, 31.9, 29.4, 29.2, 25.9, 22.7, 14.1. NMR data correspond to those published previously [76].

#### 3.3.2. *N*,*N*-Diethylnonanamide (**9**)

Diethylamine (110 mg, 1.5 mmol, 3 equiv.) was added to the solution of mixed anhydride, generated from cyclopropanol **1b** and reagent **2g** in CH_2_Cl_2_. Stirring was continued overnight (12 h) at room temperature, then refluxed for 2 h. The reaction was quenched with 10% HCl solution, then extracted with CH_2_Cl_2_ (3 × 5 mL). The combined organic layers were dried (MgSO_4_) and filtered. After evaporation of the solvent, the product was isolated by silica gel column chromatography (PE/EtOAc). Colorless oil (96 mg, 90% yield). IR (liquid film) cm^−1^ 1643. ^1^H-NMR (500 MHz, CDCl_3_) *δ* = 3.36 (q, *J* = 7.1 Hz, 2H), 3.29 (q, *J* = 7.1 Hz, 2H), 2.27 (t, *J* = 7.8 Hz, 2H), 1.71–1.53 (m, 2H), 1.38–1.20 (m, 10H), 1.16 (t, *J* = 7.1 Hz, 3H), 1.09 (t, *J* = 7.1 Hz, 3H), 0.86 (t, *J* = 6.7 Hz, 3H). ^13^C-NMR (125 MHz, CDCl_3_) *δ* = 172.7, 42.1, 40.2, 33.3, 32.0, 29.7, 29.6, 29.3, 25.7, 22.8, 14.5, 14.2, 13.2. NMR data correspond to those published previously [77].

#### 3.3.3. *N*-Methoxy*-N-*methylnonanamide (**10**)

A solution of *N*-methoxy-*N*-methylamine was prepared by mixing of *N*,*O*-dimethylhydroxylamine hydrochloride (98 mg, 1 mmol, 2 equiv.) and triethylamine (404 mg, 4 mmol, 8 equiv.) in anhydrous CH_2_Cl_2_ (5 mL) and CH_3_CN (3 mL). The obtained solution was added to the solution of mixed anhydride, generated from cyclopropanol **1b** and reagent **2g** in CH_2_Cl_2_. Stirring was continued overnight (12 h) at room temperature, then refluxed for 2 h. The reaction was quenched with 10% HCl solution, then extracted with CH_2_Cl_2_ (3 × 8 mL). The combined organic layers were washed successively with saturated NaHCO_3_, NaCl, dried (Na_2_SO_4_) and filtered. After evaporation of the solvents, the product was isolated by silica gel column chromatography (PE/EtOAc). Colorless oil (82 mg, 82% yield). IR (liquid film) cm^−1^ 1671. ^1^H-NMR (500 MHz, CDCl_3_) *δ* = 3.67 (s, 3H), 3.16 (s, 3H), 2.39 (t, *J* = 7.4 Hz, 2H), 1.66–1.55 (m, 2H), 1.37–1.17 (m, 10H), 0.86 (t, *J* = 6.9 Hz, 3H). ^13^C-NMR (125 MHz, CDCl_3_) *δ* = 175.0, 61.3, 32.0, 32.0, 29.8, 29.6, 29.5, 29.3, 24.8, 22.8, 14.2. NMR data correspond to those published previously [78].

#### 3.3.4. *N*-Benzyl*-3-*phenylpropanamide (**11**)

Benzylamine (80 mg, 0.75 mmol, 1.5 equiv.) and triethylamine (152 mg, 1.5 mmol, 3 equiv.) were added to the solution of mixed anhydride, generated from 1-phenethylcyclopropan-1-ol **1a** and reagent **2e** in CHCl_3_. Stirring was continued overnight (12 h). The reaction was quenched with 10% HCl solution, then extracted with CHCl_3_ (3 × 5 mL). The combined organic layers were dried (MgSO_4_) and filtered. After evaporation of the solvent, the product was isolated by silica gel column chromatography (PE/EtOAc). White crystalline solid (108 mg, 90% yield). ^1^H-NMR (400 MHz, CDCl_3_) *δ* = 7.33–7.25 (m, 5H), 7.25–7.18 (m, 3H), 7.17–7.12 (m, 2H), 5.59 (br.s, 1H), 4.40 (d, *J* = 5.7 Hz, 2H), 3.00 (t, *J* = 7.6 Hz, 2H), 2.52 (t, *J* = 7.6 Hz, 2H). ^13^C-NMR (100.6 MHz, CDCl_3_) *δ* = 172.0, 140.9, 138.3, 128.8, 128.7, 128.6, 127.9, 127.6, 126.4, 43.7, 38.7, 31.9. NMR data correspond to those published previously [79].

#### 3.3.5. *N*-Methoxy*-N-*methyl*-3-*phenylpropanamide (**12**)

*N*,*O*-Dimethylhydroxylamine hydrochloride (73 mg, 0.75 mmol, 1.5 equiv.) and triethylamine (152 mg, 1.5 mmol, 3 equiv.) were added to the reaction mixture containing the mixed anhydride, generated from cyclopropanol **1a** and reagent **2e** in CHCl_3_. Stirring was continued overnight (12 h). The reaction was quenched with 10% HCl solution, then extracted with CHCl_3_ (3 × 5 mL). The combined organic layers were dried (MgSO_4_) and filtered. After evaporation of the solvent, the product was isolated by silica gel column chromatography (PE/EtOAc). Colorless oil (85 mg, 88% yield). ^1^H-NMR (400 MHz, CDCl_3_) *δ* = 7.37–7.13 (m, 5H), 3.58 (s, 3H), 3.17 (s, 3H), 2.96 (t, *J* = 7.9 Hz, 2H), 2.74 (t, *J* = 7.9 Hz, 2H). ^13^C-NMR (100.6 MHz, CDCl_3_) *δ* = 173.8, 141.3, 128.5, 128.5, 126.1, 61.2, 33.8, 32.2, 30.7. NMR data correspond to those published previously [80].

#### 3.3.6. *N-(*Benzyloxy*)-3-*phenylpropanamide (**13**)

The reaction mixture containing the mixed anhydride, generated from cyclopropanol **1a** and reagent **2e** in CHCl_3_, was slowly added dropwise (within an hour) to the excess of *O*-benzylhydroxylamine BnONH_2_ (615 mg, 5.0 mmol, 10 equiv.) in anhydrous CHCl_3_ (3.5 mL). Stirring was continued overnight (10 h). After evaporation of the solvent, the product was isolated by silica gel column chromatography (PE/EtOAc). White crystalline solid (91 mg, 71% yield). ^1^H-NMR (400 MHz, CD_3_OD) *δ* = 7.41–7.23 (m, 7H), 7.23–7.15 (m, 3H), 4.71 (s, 2H), 2.89 (t, *J* = 7.5 Hz, 2H), 2.33 (t, *J* = 7.5 Hz, 2H). Note: signal of the amide NH proton was not observed due to proton exchange with the solvent. ^13^C-NMR (100.6 MHz, CD_3_OD) *δ* = 171.8, 141.7, 136.9, 130.3, 129.6, 129.5, 129.5, 129.4, 127.3, 79.0, 35.6, 32.4. NMR data correspond to those published previously [81].

#### 3.3.7. *N*-Benzylcyclohexanecarboxamide (**14**)

A solution of benzylamine (160 mg, 1.5 mmol, 3 equiv.) in CHCl_3_ (1 mL) was added to the solution of mixed anhydride, generated from 1-cyclohexylcyclopropan-1-ol **1c** and reagent **2e** in CHCl_3_. Stirring was continued overnight (12 h). The organic phase was washed with 2M aq. HCl (2 mL), water (2 mL), sat. aq. NaCl (1 mL) and organic solvent was evaporated under reduced pressure. To remove 2,4,6-trichlorobenzoic acid, the residue was treated with 2M aq. KOH (2 mL), then extracted with CH_2_Cl_2_ (3 × 2 mL) and dried (MgSO_4_). The solvent was evaporated and the obtained residue was triturated with hexane, filtered and washed with hexane to afford the title compound as white crystalline solid (84 mg, 77% yield). ^1^H-NMR (400 MHz, CDCl_3_) *δ* = 7.37–7.28 (m, 2H), 7.31–7.22 (m, 3H), 5.78 (br.s, 1H), 4.43 (d, *J* = 5.6 Hz, 2H), 2.11 (tt, *J* = 11.8, 3.5 Hz, 1H), 1.93–1.84 (m, 2H), 1.84–1.70 (m, 2H), 1.70–1.61 (m, 1H), 1.59–1.39 (m, 2H), 1.34–1.13 (m, 3H). ^13^C-NMR (100.6 MHz, CDCl_3_) *δ* = 176.0, 138.7, 128.8, 127.8, 127.6, 45.7, 43.5, 29.9, 25.9. HRMS (ESI) *m/z* calcd for C_14_H_20_NO^+^ [M+H]^+^ 218.1539, found 218.1541. NMR data correspond to those published previously [82].

#### 3.3.8. *tert*-Butyl *4-(*morpholine*-4-*carbonyl*)*piperidine*-1-*carboxylate (**15**)

A solution of morpholine (130 mg, 1.5 mmol, 3 equiv.) in CHCl_3_ (1 mL) was added to the solution of mixed anhydride, generated from the *tert*-butyl 4-(1-hydroxycyclopropyl)piperidine-1-carboxylate **1d** and reagent **2e** in CHCl_3_. Stirring was continued overnight (12 h). The organic phase was washed with 2M aq. HCl (2 mL), water (2 mL), sat. aq. NaCl (1 mL) and the organic solvent was evaporated under reduced pressure. To remove 2,4,6-trichlorobenzoic acid, the residue was treated with 2M aq. KOH (2 mL), then extracted with CH_2_Cl_2_ (3 × 2 mL) and dried (MgSO_4_). The solvent was evaporated and the obtained residue was triturated with hexane, filtered and washed with hexane to afford the title compound as white crystalline solid (122 mg, 82% yield). ^1^H-NMR (400 MHz, CDCl_3_) *δ* = 4.14 (br.s, 2H), 3.73–3.64 (m, 4H), 3.64–3.56 (m, 2H), 3.55–3.44 (m, 2H), 2.85–2.65 (m, 2H), 2.64–2.53 (m, 1H), 1.83–1.58 (m, 4H), 1.44 (s, 9H). ^13^C-NMR (100.6 MHz, CDCl_3_) *δ* = 173.2, 154.8, 79.7, 67.1, 66.9, 46.1, 43.3, 42.2, 38.4, 28.6, 28.5. HRMS (ESI) *m/z* calcd for C_15_H_26_N_2_O_4_Na^+^ [M+Na]^+^ 321.1785, found 321.1783.

#### 3.3.9. *N-(2-*Hydroxy*-2-*methylpropyl*)-3-*phenylpropanamide (**16**)

A solution of DIPEA (194 mg, 1.5 mmol, 3 equiv.) and 1-amino-2-methylpropan-2-ol (45 mg, 0.5 mmol, 1.0 equiv.) in CHCl_3_ (1 mL) was added to the solution of mixed anhydride, generated from cyclopropanol **1a** and reagent **2e** in CHCl_3_. Stirring was continued overnight (12 h). The organic phase was washed with 2M aq. HCl (2 mL), water (2 mL), sat. aq. NaCl (1 mL) and the organic solvent was evaporated under reduced pressure. To remove 2,4,6-trichlorobenzoic acid, the residue was treated with 2M aq. KOH (1 mL), then extracted with CH_2_Cl_2_ (3 × 2 mL) and dried (Na_2_SO_4_). Solvents were evaporated and the obtained residue was triturated with hexane, filtered and washed with hexane to afford the title compound as white crystalline solid (90 mg, 81% yield). ^1^H-NMR (400 MHz, CDCl_3_) *δ* = 7.32–7.23 (m, 2H), 7.24–7.15 (m, 3H), 5.85 (br.s, 1H), 3.21 (d, *J* = 6.0 Hz, 2H), 2.98 (t, *J* = 7.6 Hz, 2H), 2.53 (t, *J* = 7.6 Hz, 2H), 2.35 (br.s, 1H), 1.13 (s, 6H). ^13^C-NMR (100.6 MHz, CDCl_3_) *δ* = 173.2, 140.8, 128.7, 128.5, 126.5, 70.9, 50.4, 38.6, 31.9, 27.3. HRMS (ESI) *m/z* calcd for C_13_H_20_NO_2_^+^ [M+H]^+^ 222.1489, found 222.1484. NMR data correspond to those published previously [83].

#### 3.3.10. Ethyl *4-(3-*phenylpropanamido)benzoate (**17**)

A solution of *N*-methylimidazole (123 mg, 1.5 mmol, 3 equiv.) and ethyl 4-aminobenzoate (83 mg, 0.5 mmol, 1.0 equiv.) in CHCl_3_ (1 mL) was added to the solution of mixed anhydride, generated from cyclopropanol **1a** and reagent **2e** in CHCl_3_. Stirring was continued overnight (12 h). The reaction was quenched with 2M aq. HCl (2 mL), then extracted with CHCl_3_ (3 × 5 mL). The combined organic layers were dried (MgSO_4_) and filtered. After evaporation of the solvent, the product was isolated by silica gel column chromatography (PE/EtOAc). White crystalline solid (125 mg, 84% yield). ^1^H-NMR (400 MHz, CDCl_3_) *δ* = 8.00–7.95 (m, 2H), 7.51 (d, *J* = 8.6 Hz, 2H), 7.35–7.27 (m, 3H), 7.25–7.19 (m, 3H), 4.35 (q, *J* = 7.1 Hz, 2H), 3.05 (t, *J* = 7.6 Hz, 2H), 2.69 (t, *J* = 7.6 Hz, 2H), 1.38 (t, *J* = 7.1 Hz, 3H). ^13^C-NMR (100.6 MHz, CDCl_3_) *δ* = 170.7, 166.3, 142.0, 140.5, 130.9, 128.8, 128.5, 126.6, 126.1, 118.9, 61.0, 39.7, 31.5, 14.5. HRMS (ESI) *m/z* calcd for C_18_H_20_NO_3_^+^ [M+H]^+^ 298.1438, found 298.1437.

#### 3.3.11. (*E*)-*N*-Benzyl-12-(benzyloxy)dodec-6-enamide (**18**)

Benzylamine (160 mg, 1.5 mmol, 3 equiv.) was added to the solution of mixed anhydride, generated from *exo*-7-(5-(benzyloxy)pentyl)bicyclo[4.1.0]heptan-1-ol (**1f**) and reagent **2e** in CHCl_3_. Stirring was continued overnight (12 h). The reaction was quenched with 10% HCl solution, then extracted with CHCl_3_ (3 × 5 mL). The combined organic layers were dried (Na_2_SO_4_) and filtered. After evaporation of the solvent, the white product (118 mg, 60%) was isolated by silica gel column chromatography (PE/EtOAc). IR (CCl_4_) cm^−1^ 3453, 3090, 3068, 3032, 1683. ^1^H-NMR (400 MHz, CDCl_3_) *δ* = 7.39–7.23 (m, 10H), 5.71 (br.s, 1H), 5.45–5.30 (m, 2H), 4.49 (s, 2H), 4.44 (d, *J* = 5.6 Hz, 2H), 3.45 (t, *J* = 6.6 Hz, 2H), 2.25–2.14 (m, 2H), 2.07–1.92 (m, 4H), 1.73–1.53 (m, 6H), 1.46–1.29 (m, 4H). ^13^C-NMR (100.6 MHz, CDCl_3_) *δ* = 173.0, 138.9, 138.6, 130.9, 130.0, 128.9, 128.5, 128.0, 127.8, 127.7, 127.6, 73.0, 70.6, 43.8, 36.8, 32.6, 32.4, 29.8, 29.5, 29.4, 25.9, 25.4. Found: C, 79.29; H, 8.95%. C_26_H_35_NO_2_ requires C, 79.35; H, 8.96%.

#### 3.3.12. 6-((3S,9S,14aR)-9-Benzyl-6,6-dimethyl-1,4,7,10-tetraoxotetradecahydropyrrolo[1,2-a][1,4,7,10]tetraazacyclododecin-3-yl)-*N*-(benzyloxy)hexanamide (cyclopeptide **19**)

Preparation of this compound and its characterization has been described by us previously [39].

#### 3.3.13. Benzyl nonanoate (**20**)

Benzyl alcohol (54 mg, 0.5 mmol, 1.0 equiv.) and DMAP (122 mg, 1.0 mmol, 2.0 equiv.) were added to the solution of mixed anhydride, generated from cyclopropanol **1b** and reagent **2g** in CH_2_Cl_2_. Stirring was continued overnight (12 h). The reaction was quenched with 10% HCl solution, then extracted with CH_2_Cl_2_ (3 × 5 mL). The combined organic layers were dried (Na_2_SO_4_) and filtered. After evaporation of the solvent, the product was isolated by silica gel column chromatography (PE/EtOAc). Colorless oil (114 mg, 92% yield). IR (liquid film) cm^−1^ 3091, 3067, 3035, 1739. ^1^H-NMR (400 MHz, CDCl_3_) *δ* = 7.50–7.28 (m, 5H), 5.12 (s, 2H), 2.36 (t, *J* = 7.5 Hz, 2H), 1.65 (quint., *J* = 7.5 Hz, 2H), 1.40–1.20 (m, 10H), 0.89 (t, *J* = 6.9 Hz, 3H). ^13^C-NMR (100.6 MHz, CDCl_3_) *δ* = 173.8, 136.3, 128.7, 128.3, 66.2, 34.5, 31.9, 29.3, 29.3, 29.2, 25.1, 22.8, 14.2.

#### 3.3.14. Phenyl 3-phenylpropanoate (**22**)

Method A: A solution of DIPEA (194 mg, 1.5 mmol, 3 equiv.) and phenol (52 mg, 0.55 mmol, 1.1 equiv.) in CHCl_3_ (1 mL) was added to the solution of mixed anhydride, generated from cyclopropanol **1a** and reagent **2e** in CHCl_3_. Stirring was continued overnight (12 h). The reaction was quenched with 2M aq. HCl (2 mL), then extracted with CHCl_3_ (3 × 5 mL). The combined organic layers were dried (MgSO_4_) and filtered. After evaporation of the solvent, the product was isolated by silica gel column chromatography (PE/EtOAc). Colorless oil (86 mg, 76% yield).

Method B [65]: A solution of phenol (47 mg, 0.5 mmol, 1.0 equiv.) in CHCl_3_ (0.5 mL) was added to the solution of mixed TFA anhydride, generated from cyclopropanol **1a** and reagent **2b** (PIFA) in CHCl_3_. Stirring was continued overnight (12 h). The reaction was quenched with sat. aq. NaHCO_3_ solution, then extracted with CHCl_3_ (3 × 3 mL). The combined organic layers were dried (MgSO_4_) and filtered. After evaporation of the solvent, the product was isolated by silica gel column chromatography (PE/EtOAc). Colorless oil (79 mg, 70% yield). ^1^H-NMR (400 MHz, CDCl_3_) *δ* = 7.37–7.28 (m, 4H), 7.27–7.17 (m, 4H), 7.01–6.99 (m, 1H), 6.99–6.97 (m, 1H), 3.06 (t, *J* = 7.7 Hz, 2H), 2.90–2.84 (m, 2H). ^13^C-NMR (100.6 MHz, CDCl_3_) *δ* = 171.5, 150.8, 140.3, 129.5, 128.7, 128.5, 126.6, 125.9, 121.7, 36.1, 31.1. HRMS (ESI) *m/z* calcd for C_15_H_14_O_2_Na^+^ [M+Na]^+^ 249.0886, found 249.0888. NMR data correspond to those published previously [84].

#### 3.3.15. Pentafluorophenyl 3-phenylpropanoate (**23**)

A solution of DIPEA (194 mg, 1.5 mmol, 3 equiv.) and pentafluorophenol (101 mg, 0.55 mmol, 1.1 equiv.) in CHCl_3_ (1 mL) was added to the solution of mixed anhydride, generated from cyclopropanol **1a** and reagent **2e** in CHCl_3_. Stirring was continued overnight (12 h). The reaction was quenched with 2M aq. HCl (2 mL), then extracted with CHCl_3_ (3 × 5 mL). The combined organic layers were dried (MgSO_4_) and filtered. After evaporation of the solvent, the product was isolated by silica gel column chromatography (PE/EtOAc). Colorless oil (137 mg, 87% yield). ^1^H-NMR (400 MHz, CDCl_3_) *δ* = 7.37–7.31 (m, 2H), 7.28–7.23 (m, 3H), 3.15–3.05 (m, 2H), 3.03–2.97 (m, 2H). ^13^C-NMR (100.6 MHz, CDCl_3_) *δ* = 168.9, 139.4, 128.9, 128.4, 126.9, 35.1, 30.8. Note: the signals of carbon atoms from the C_6_F_5_ fragment has been accumulated with low S/N ratio due to coupling on fluorine atoms, and therefore cannot be reliably described. HRMS (ESI) *m/z* calcd for C_15_H_9_F_5_O_2_Na^+^ [M+Na]^+^ 339.0415, found 339.0420.

#### 3.3.16. *S*-(4-Methoxybenzyl) 3-phenylpropanethioate (**24**)

A solution of DIPEA (194 mg, 1.5 mmol, 3 equiv.) and (4-methoxyphenyl)methanethiol (85 mg, 0.55 mmol, 1.1 equiv.) in CHCl_3_ (1 mL) was added to the solution of mixed anhydride, generated from cyclopropanol **1a** and reagent **2e** in CHCl_3_. Stirring was continued overnight (12 h). The reaction was quenched with 2M aq. HCl (2 mL), then extracted with CHCl_3_ (3 × 5 mL). The combined organic layers were dried (MgSO_4_) and filtered. After evaporation of the solvent, the product was isolated by silica gel column chromatography (PE/EtOAc). Colorless oil (92 mg, 64% yield). ^1^H-NMR (400 MHz, CDCl_3_) *δ* = 7.32–7.25 (m, 2H), 7.25–7.14 (m, 5H), 6.86–6.80 (m, 2H), 4.09 (s, 2H), 3.79 (s, 3H), 3.04–2.95 (m, 2H), 2.91–2.84 (m, 2H). ^13^C-NMR (100.6 MHz, CDCl_3_) *δ* = 198.5, 159.3, 140.5, 130.4, 130.0, 129.0, 128.8, 126.8, 114.5, 55.8, 45.7, 33.2, 31.9. HRMS (ESI) *m/z* calcd for C_17_H_18_O_2_SNa^+^ [M+Na]^+^ 309.0920, found 309.0915.

### 3.4. General Procedure for the Synthesis of Macrolactones ***25a**,**b***

To a solution of reagent **2e** (430 mg, 0.66 mmol, 2 equiv.) in anhydrous CH_2_Cl_2_ (140 mL, 0.005 M) a solution of scandium(III) triflate (32 mg, 0.066 mmol, 0.2 equiv.) in anhydrous CH_3_CN (0.7 mL) was added dropwise with stirring under inert (argon) atmosphere. To the resulting mixture a solution of 1-(11-hydroxyundecyl)cyclopropan-1-ol **1h** (75 mg, 0.33 mmol) in anhydrous CH_2_Cl_2_ (9 mL) was added dropwise over 19 h under reflux conditions and stirring. Upon completion of the addition, the mixture was refluxed for 7 days, cooled to room temperature, and treated with saturated aqueous NaHCO_3_ solution (30 mL) until pH~8. The aqueous phase was separated and extracted with diethyl ether (2 × 30 mL). The combined organic extracts were washed with brine (2 × 15 mL) and dried with Na_2_SO_4_. After evaporation of solvents under reduced pressure and purification of the residue by silica gel column chromatography (eluent: PE, then PE/EtOAc), oxacyclotridecan-2-one **25b** (59 mg, 91% yield) and 1,14-dioxacyclohexacosane-2,15-dione **26b** (5 mg, 8% yield) were obtained.

Analogously oxacycloundecan-2-one **25a** (35 mg, 62% yield) and 1,12-dioxacyclodocosane-2,13-dione **26a** (8 mg, 14% yield) were obtained from 1-(9-hydroxynonyl)cyclopropan-1-ol **1g** with reagent **2g**. The reactions were carried out for a total of 17 h (addition of substrate during 11 h following with heating of the reaction mixture under reflux conditions during 6 h).

#### 3.4.1. Oxacycloundecan-2-one (**25a**)

Colorless oil. IR (liquid film) cm^−1^ 1731. ^1^H-NMR (400 MHz, CDCl_3_) *δ* = 4.20–4.14 (m, 2H), 2.36–2.31 (m, 2H), 1.79–1.67 (m, 4H), 1.56–1.47 (m, 2H), 1.47–1.22 (m, 8H). ^13^C-NMR (100.6 MHz, CDCl_3_) *δ* = 174.3, 64.9, 35.4, 26.4, 25.6, 25.5, 24.9, 24.3, 22.7, 21.6. HRMS (ESI) calcd for C_10_H_19_O_2_^+^ [M+H]^+^ 171.1380, found 171.1392. NMR data correspond to those published previously [85].

#### 3.4.2. Oxacyclotridecan-2-one (**25b**)

Colorless oil. IR (liquid film) cm^−1^ 1734. ^1^H-NMR (400 MHz, CDCl_3_) *δ* = 4.18–4.12 (m, 2H), 2.39–2.32 (m, 2H), 1.74–1.59 (m, 4H), 1.47–1.27 (m, 14H). ^13^C-NMR (100.6 MHz, CDCl_3_) *δ* = 174.3, 64.7, 34.8, 27.6, 26.8, 26.6, 25.6, 25.6, 25.2, 24.7, 24.4. HRMS (ESI) calcd for C_12_H_23_O_2_^+^ [M+H]^+^ 199.1693, found 199.1690. NMR data correspond to those published previously [86].

### 3.5. Preparation of (E)-oxacyclotridec-7-en-2-one (***28***)

To a solution of reagent **2e** (181 mg, 0.277 mmol, 1.08 equiv.) in anhydrous CH_2_Cl_2_ (61 mL, 0.005 M) a solution of scandium(III) triflate (25 mg, 0.051 mmol, 0.2 equiv.) in anhydrous CH_3_CN (0.3 mL) was added dropwise with stirring under inert (argon) atmosphere. To the resulting mixture a solution of *exo*-7-(5-hydroxypentyl)bicyclo[4.1.0]heptan-1-ol (**1i**, 51 mg, 0.258 mmol, 1.0 equiv.) in anhydrous CH_2_Cl_2_ (9 mL) was added dropwise over 19 h under reflux conditions and stirring under inert atmosphere. Upon completion of the addition, the mixture was refluxed for 26 h, cooled to room temperature, and treated with saturated aqueous NaHCO_3_ solution (35 mL) until pH~8. The aqueous phase was separated and extracted with CHCl_3_ (2 × 15 mL). The combined organic extracts were washed with brine (1 × 10 mL) and dried with MgSO_4_. After evaporation of solvents under reduced pressure, the product was isolated by silica gel column chromatography (eluent: PE, then PE/EtOAc). Colorless oil (15 mg, 30% yield). Isolated product was contaminated with about 13% of inseparable by column chromatography impurity, likely diolide. IR (CCl_4_) cm^−1^ 1741, 1580, 976. ^1^H-NMR (500 MHz, CDCl_3_) *δ* = 5.47–5.38 (m, 1H), 5.29–5.20 (m, 1H), 4.11 (t, *J* = 5.0 Hz, 2H), 2.30–2.24 (m, 2H), 2.06–1.99 (m, 2H), 1.98–1.92 (m, 2H), 1.73–1.65 (m, 2H), 1.56–1.49 (m, 2H), 1.49–1.37 (m, 6H). ^13^C-NMR (125 MHz, CDCl_3_) *δ* = 173.7, 132.4, 130.5, 63.3, 35.4, 31.0, 30.7, 29.2, 27.1, 25.9, 23.2, 22.2.

## 4. Conclusions

In conclusion, we have demonstrated that cyclopropanols can serve as useful precursors of versatile mixed anhydrides, which can be generated by oxidative fragmentation of the cyclopropane ring with phenyliodine(III) dicarboxylates in inert solvents, such as chloroform or dichloromethane. The reaction is especially facile and affords high 95–98% yields of the corresponding mixed anhydrides with phenyliodine(III) dicarboxylates bearing electron-withdrawing carboxylate ligands, e.g., trifluoroacetate, 3-nitro- or 2,4,6-trichlorobenzoate. Mixed anhydrides generated by this approach are suitable to perform the most common acylation reactions, delivering a range of amides, thioester, esters, 11- and 13-membered macrocyclic lactones in good to excellent yields. The reaction conditions are mild and suitable for the last-stage installation of amide functionality into the peptide scaffold. The developed protocol can be used in step-economical syntheses of natural products based on cyclopropanol-ring cleavage strategies, along with generation of molecular libraries from the single cyclopropanol precursor in the framework of diversity-oriented synthesis paradigm. Furthermore, in view of general importance of acyl-transfer reactions from mixed anhydrides [49,87,88], the new method of their generation could serve as a beneficial supplement to the existing approaches.

## Figures and Tables

**Table 1:** Oxidation fragmentation of cyclopropanol **1a** with phenyliodine(III) reagents. ^a^

Entry	Reagent	Solvent	Additive or Catalyst	Reaction Time, Min ^b^	Main Product	Yield % ^c^	Yield of 5, % ^c^
1	**2a**	CD_3_OD	–	<15	**4**	93	–
2	**2a**	CDCl_3_	BnNH_2_ (3 equiv.)	300	no reaction		
3	**2a**	CDCl_3_	–	600 ^d^	**3a**	50	12
4	**2a**	CDCl_3_	TfOH (1 mol%)	<5	**3a**	90	10 ^e^
5	**2b**	CDCl_3_	–	7	**3b**	**95**	Trace ^f^
6	**2c**	CDCl_3_	–	1500	– ^g^	– ^g^	– ^g^
7	**2c**	CDCl_3_	TfOH (1 mol%)	1500	– ^g^	– ^g^	– ^g^
8	**2d**	CDCl_3_	–	500 ^d^	**3d**	46	8
9	**2e**	CDCl_3_	–	100	**3e**	**98**	2
10	**2e**	CDCl_3_	TfOH (1 mol%)	<5	**3e**	70	30 ^e^
11	**2e**	CDCl_3_	MsOH (1 mol%)	30	**3e**	95	4 ^e^
12	**2e**	CDCl_3_	CF_3_CO_2_H (1 mol%)	90	**3e**	94	5 ^e^
13	**2e**	toluene-*d*_8_	–	<15	**3e**	90	4
14	**7**	CD_3_ODor CDCl_3_	–	1500	no reaction		

^a^ Reactions were run in NMR tubes with approx. 0.0375 mmol of **1a** and **2a–d** in 0.75 mL of the respective solvent (0.05 M). Progress was monitored by ^1^H-NMR spectroscopy. ^b^ Time required for full conversion of **1a**. ^c^ Yields determined by ^1^H- NMR. ^d^ Time required for ~90% conversion of **1a**, full conversion was achieved within 24 h. ^e^ Yield of **6**. ^f^ 1-Phenylpentan-3-one was formed in ~4% yield. ^g^ Not determined, complex mixture of products.

**Table 2:**
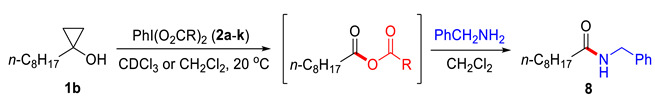
Acylation of benzylamine with mixed anhydrides generated from 1-octylcyclopropanol **1b** and phenyliodine(III) dicarboxylates **2a**-**k**.^a.^

Entry	Reagent 2a-k	R	Time, min ^b^	Yield of 8, % ^c^
1	**2a**	Me	120	22 ^e,d^
2	**2h**	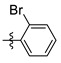	95	91
3	**2e**	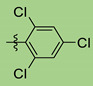	30	94
4	**2i**	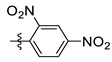	<14	88
5	**2g**	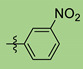	90	95
6	**2j**	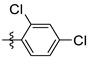	– ^f^	58
7	**2k**	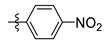	– ^f^	– ^f^

^a^ Preparative reaction were run with 1 mmol of **1b** and 1.07 mmol of **2a**-**k** in CH_2_Cl_2_. After the full consumption of cyclopropanol **1b** (TLC control), benzylamine (3 mmol, 3 equiv.) was added. ^b^ The reaction progress was monitored by ^1^H-NMR in CDCl_3_ as a separate experiment (~0.03 M **1b**). ^c^ Yield of isolated product. ^d^ After the aqueous work-up, 8-oxoundecyl acetate by-product was isolated in 12% yield. ^e^ *N*-Benzylacetamide was formed as major product in 44% yield. ^f^ Not determined due to low solubility of **2k** and **2j** and slow reaction rates.

**Table 3:**
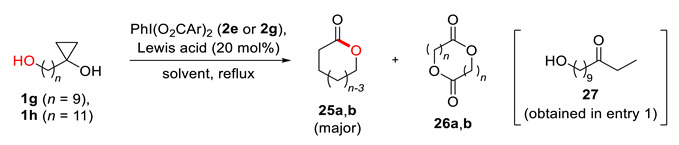
Intramolecular *O*-acylation: synthesis of macrolactones **25a**,**b**.

Entry	Substrate	Reagent	Catalyst	Solvent	Time, h	Yield of 25, % ^a^	Yield of 26, % ^a^
1	**1g** (*n* = 9)	**2g**	Sc(OTf)_3_	CH_3_CN/THF	20	0 ^b^	0
2		**2g**	Sc(OTf)_3_	CH_2_Cl_2_/CH_3_CN ^c^	17	62	14
3	**1h** (*n* = 11)	**2g**	Sc(OTf)_3_	CH_2_Cl_2_/CH_3_CN	189	82	10
4		**2e**	Sc(OTf)_3_	CH_2_Cl_2_/CH_3_CN	187	91	8
5		**2g**	Hf(OTf)_4_	CH_2_Cl_2_/CH_3_CN	178	93	>7

^a^ Yields of isolated products. ^b^ 12-Hydroxydodecan-3-one **27** was isolated in 81% yield. ^c^ Acetonitrile was used as a solubilizing additive for Sc(OTf)_3_, ca. 1 mL per 150 mL of CH_2_Cl_2_.

## Data Availability

The data presented in this study are available in the article and supplementary material.

## Supplementary Materials

Additional experimental protocols, characterization of mixed anhydrides **3a** and **3e**, characterization of by-products, preparation of cyclopropanols **1g** and **1h**, copies of ^1^H and ^13^C-NMR spectra.
