# Supplementary material for: Generation of Mixed Anhydrides via Oxidative Fragmentation of Tertiary Cyclopropanols with Phenyliodine(III) Dicarboxylates
Source: Molecules. 2020 Dec 30;26(1):140. doi: 10.3390/molecules26010140 (PMC7794720; doi:10.3390/molecules26010140)
Supplement: Supplementary file 1 [file molecules-26-00140-s001.pdf]

*Supplementary Material*

# Generation of Mixed Anhydrides via Oxidative Fragmentation of Tertiary Cyclopropanols with Phenyliodine(III) Dicarboxylates

**Dzmitry M. Zubrytski [1], Gábor Zoltán Elek [2], Margus Lopp [2] and Dzmitry G. Kananovich [2,*]**

[1] Belarusian State University, Department of Organic Chemistry, Leningradskaya 14, Minsk 220050, Belarus; zubrytski_dzmitry@rambler.ru (D.M.Z.)

[2] Tallinn University of Technology, School of Science, Department of Chemistry and Biotechnology, Akadeemia tee 15, Tallinn 12618, Estonia; gabor.elek@taltech.ee (G.Z.E.); margus.lopp@taltech.ee (M.L.)

* Correspondence: dzmitry.kananovich@taltech.ee; Tel.: +372-6204382

## Table of Content

1. Oxidative fragmentation of cyclopropanols with phenyliodine(III) dicarboxylates: characterization of mixed anhydrides S2
2. Characterization of by-products S6
3. Preparation of cyclopropanols S7
4. Copies of $^{1}H$ and $^{13}C$ NMR spectra S9
5. References S31

## 1. Oxidative fragmentation of cyclopropanols with phenyliodine(III) dicarboxylates: characterization of mixed anhydrides

Selected experiments from **Table 1**:

**Table 1, Entry 3**: Reaction of **1a** with PIDA reagent (**2a**) in $CDCl_3$.

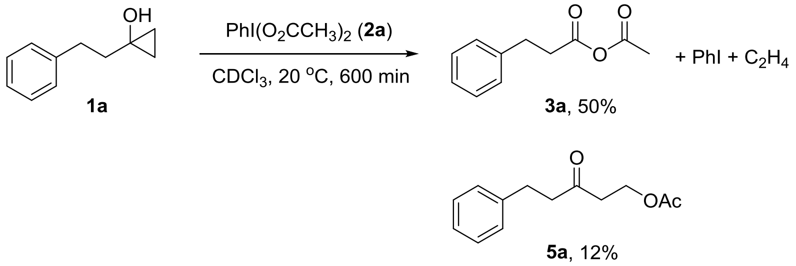


A solution of cyclopropanol **1a** (6.1 mg, 0.0375 mmol) in anhydrous $CDCl_3$ (0.37 mL) was mixed with solution of PIDA reagent **2a** (12.1 mg, 0.0375 mmol, 1.05 equiv.) in anhydrous $CDCl_3$ (0.37 mL) and progress of the reaction was monitored by $^1H$ NMR spectroscopy. Initial concentrations of both reactants nearly equal to 0.05 M. Conversion of starting materials **1a**, **2a** and accumulation of product **3a** were calculated from the integral intensities of the corresponding signals. Thus, consumption of **1a** was followed by its characteristic signals at 1.88, 0.77, 0.46 ppm, consumption of **2a** – by the signal at 8.09 ppm, accumulation of product **3a** – by its signal at 2.19 ppm. Approximately 90% consumption of the starting materials was observed after 600 min.

Characteristic NMR resonances of mixed acetic acid anhydride **3a**: $^1H$ NMR (400 MHz, $CDCl_3$) δ = 2.99 (t, *J* = 7.7 Hz, 2H, PhC$\underline{H}_2$), 2.78 (t, *J* = 7.7 Hz, 2H, C$\underline{H}_2$CO), 2.19 (s, 3H, C$\underline{H}_3$CO). $^{13}C$ NMR (100.6 MHz, $CDCl_3$) δ = 168.7 (CH$_2\underline{C}$O), 166.5 (CH$_3\underline{C}$O), 36.9 ($\underline{C}$H$_2$CO), 30.3 (Ph$\underline{C}$H$_2$), 22.4 ($\underline{C}$H$_3$CO). Signal assignment is based on HSQC and HMBC data. Aromatic regions of NMR spectra have not been analyzed due to overlap with PhI signals.
HRMS (ESI): calcd. for $C_{11}H_{12}O_3Na^+$ $[M+Na]^+$ 215.0679, found *m*/*z* 215.0670.

Characteristic NMR resonance of β-acetyloxyketone by-product **5a**: $^1H$ NMR (400 MHz, $CDCl_3$) δ = 4.32 (t, *J* = 6.2 Hz, 2H, C$\underline{H}_2$COAc), 2.01 (s, 3H, C$\underline{H}_3$CO$_2$). $^{13}C$ NMR (100.6 MHz, $CDCl_3$) δ = 59.4 ($\underline{C}$H$_2$COAc).
HRMS (ESI): calcd. for $C_{13}H_{16}O_3Na^+$ $[M+Na]^+$ 243.0992, found *m*/*z* 243.0983.

The corresponding β-acetyloxyketone by-product was also isolated in the oxidation of 1-octylcyclopropanol **1b** with PIDA reagent, see section 2 below.

**Table 1, Entry 4**: Reaction of cyclopropanol **1a** with PIDA reagent (**2a**) in $CDCl_3$ in the presence of catalytic amounts of TfOH (1 mol%).

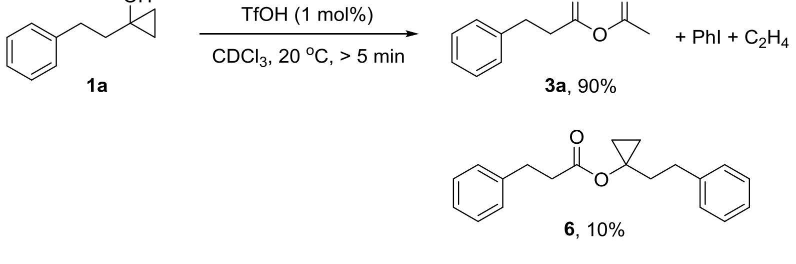

A solution of TfOH (40 μL, ca. 0.01 M in $CDCl_3$, 1 mol%) was added to solution of reagent **2a** (12.9 mg, 0.04 mmol, 1.1 equiv.) in anhydrous $CDCl_3$ (0.37 mL) and then mixed with a solution of cyclopropanol **1a** (6.1 mg, 0.0375 mmol) in anhydrous $CDCl_3$ (0.37 mL). Progress of the reaction was monitored by $^1$H NMR spectroscopy. Full consumption of the starting material **1a** was observed immediately after acquisition of the first $^1$H NMR spectrum (less than in 5 min). Yields of products **3a** and **6** were calculated from the integral intensities of the corresponding $^1$H NMR signals.

Characteristic NMR resonances of mixed acetic acid anhydride **3a**: $^1$H NMR (400 MHz, $CDCl_3$) δ = 2.99 (t, *J* = 7.7 Hz, 2H, PhC$\underline{H}_2$), 2.78 (t, *J* = 7.7 Hz, 2H, C$\underline{H}_2$CO), 2.19 (s, 3H, C$\underline{H}_3$CO). $^{13}$C NMR (100.6 MHz, $CDCl_3$) δ = 168.7 ($CH_2\underline{C}O$), 166.5 ($CH_3\underline{C}O$), 36.9 ($\underline{C}H_2CO$), 30.3 ($Ph\underline{C}H_2$), 22.4 ($\underline{C}H_3CO$). Signal assignment is based on HSQC and HMBC data. Aromatic regions of NMR spectra have not been analyzed due to overlap with PhI signals.
HRMS (ESI): calcd. for $C_{11}H_{12}O_3Na^+$ $[M+Na]^+$ 215.0679, found *m/z* 215.0670.

Characteristic NMR signals for by-product **6** (aromatic region of NMR spectra has not been analyzed): $^1$H NMR (400 MHz, $CDCl_3$) δ = 2.89 (t, *J* = 7.7 Hz, 2H), 2.67 (m, 2H), 2.49 (t, *J* = 7.7 Hz, 2H), 2.05 (m, 2H), 0.76 (m, 2H), 0.62 (m, 2H). $^{13}$C NMR (100.6 MHz, $CDCl_3$) δ = 173.0 (CO), 36.3 ($CH_2$), 36.1 ($CH_2$), 32.4 ($CH_2$), 31.1 ($CH_2$), 12.0 ($2CH_2$ cp).
HRMS (ESI): calcd. for $C_{20}H_{22}O_2Na^+$ $[M+Na]^+$ 317.1512, found *m/z* 317.1515.

**Table 1, Entry 9:** Reaction of **1a** with **2e** in $CDCl_3$.

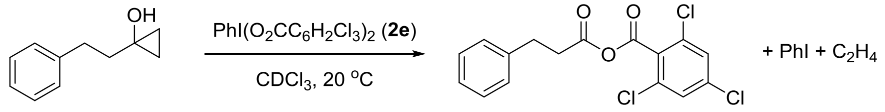


**1a** **3e**

A solution of cyclopropanol **1a** (6.1 mg, 0.0375 mmol) in anhydrous $CDCl_3$ (0.37 mL) was mixed with solution of reagent **2e** (26 mg, 0.04 mmol, 1.05 equiv.) in anhydrous $CDCl_3$ (0.37 mL) and progress of the reaction was monitored by $^1$H NMR spectroscopy. Initial concentrations of both reactants nearly equal to 0.05 M. Conversion of starting materials **1a**, **2e** and accumulation of product **3e** were calculated from the integral intensities of the corresponding signals. Thus, consumption of **1a** was followed by its characteristic signals at 1.88, 0.77, 0.46 ppm, consumption of **2e** – by the signal at 8.26 ppm, accumulation of product **3e** – by its signal at 3.04 ppm. Full consumption of the starting material **1a** was observed within 100 min.

According to $^1$H and $^{13}$C NMR analysis (see Figures S1 and S2 below), the reaction mixture after the completion of reaction contained mixed anhydride **3e**, 2,4,6-trichlorobenzoic acid, iodobenzene and ethylene as reaction products (see copies of NMR spectra below). By integration of $^1$H NMR spectrum, yield of **3e** can be estimated as 98%. The corresponding β-acyloxy ketone by-product **5e** has a characteristic signal of C$\underline{H}_2$OCO$_2$Ar protons at 4.65 ppm (t, *J* = 6.2 Hz, 1H) in $^1$H NMR spectra and its yield does not exceed 2%. Characteristic NMR resonances of mixed anhydride **3e**: $^1$H NMR (400 MHz, $CDCl_3$) δ = 7.37 (s, 2H, $C_6\underline{H}_2Cl_3$), 3.04 (m, 2H, PhC$\underline{H}_2$), 2.91 (m, 2H, C$\underline{H}_2$CO). $^{13}$C NMR (100.6 MHz, $CDCl_3$) δ = 167.2 ($CH_2\underline{C}O$), 159.2 ($Ar\underline{C}O$), 37.11 ($\underline{C}H_2CO$), 30.22 ($Ph\underline{C}H_2$). Signal assignment is based on HSQC and HMBC data (see Figure S3 below).
HRMS (ESI): calcd. for $C_{16}H_{11}Cl_3O_3Na^+$ $[M+Na]^+$ 378.9666, found *m/z* 378.9655.

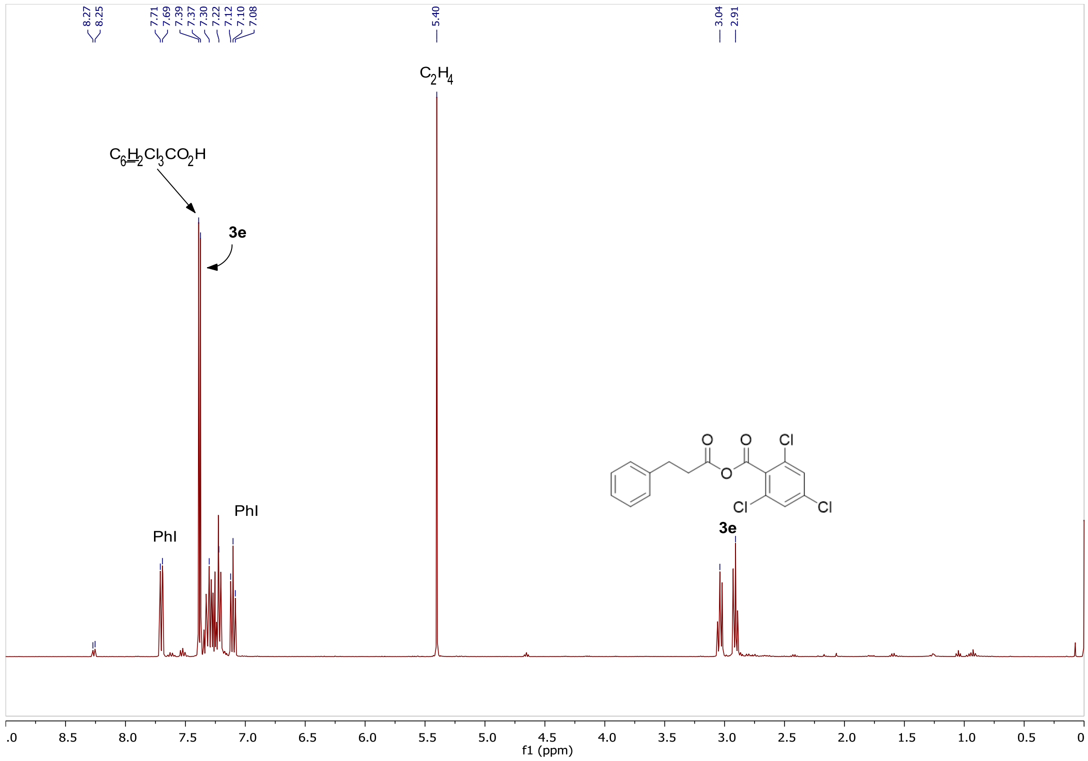


**Figure S1.** $^{1}H$ NMR of the reaction mixture after completion of the reaction with the assignment of the key signals.

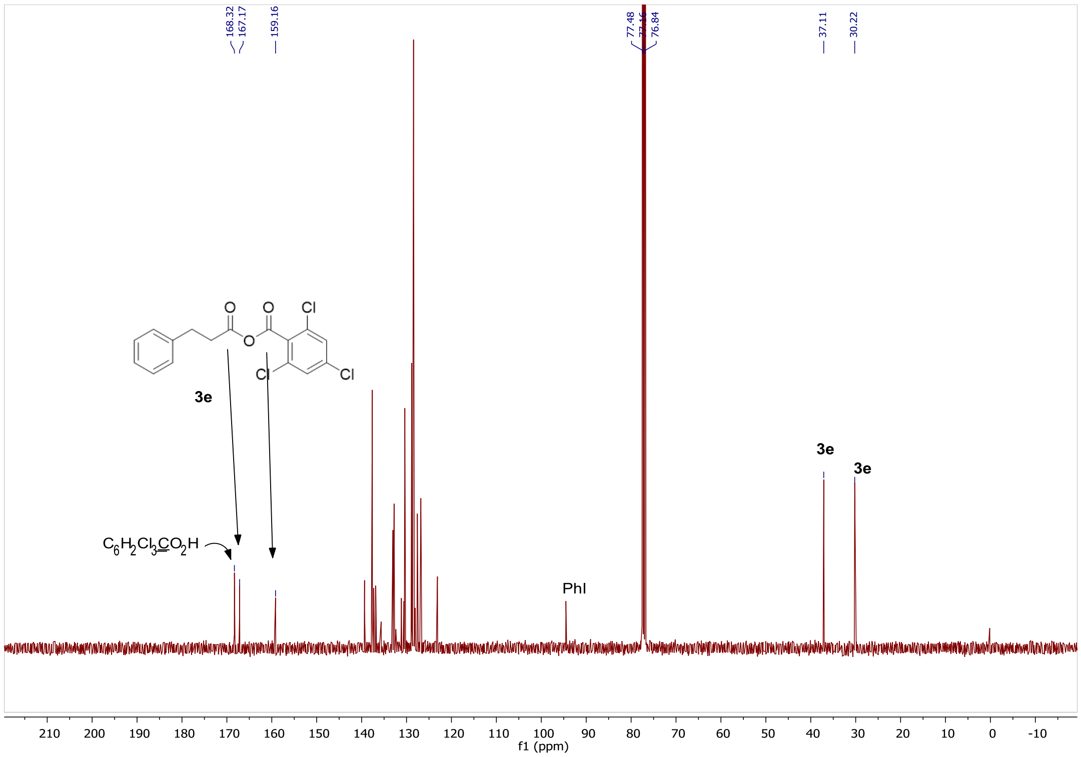


**Figure S2.** $^{13}C$ NMR of the reaction mixture after completion of the reaction with the assignment of the key signals.

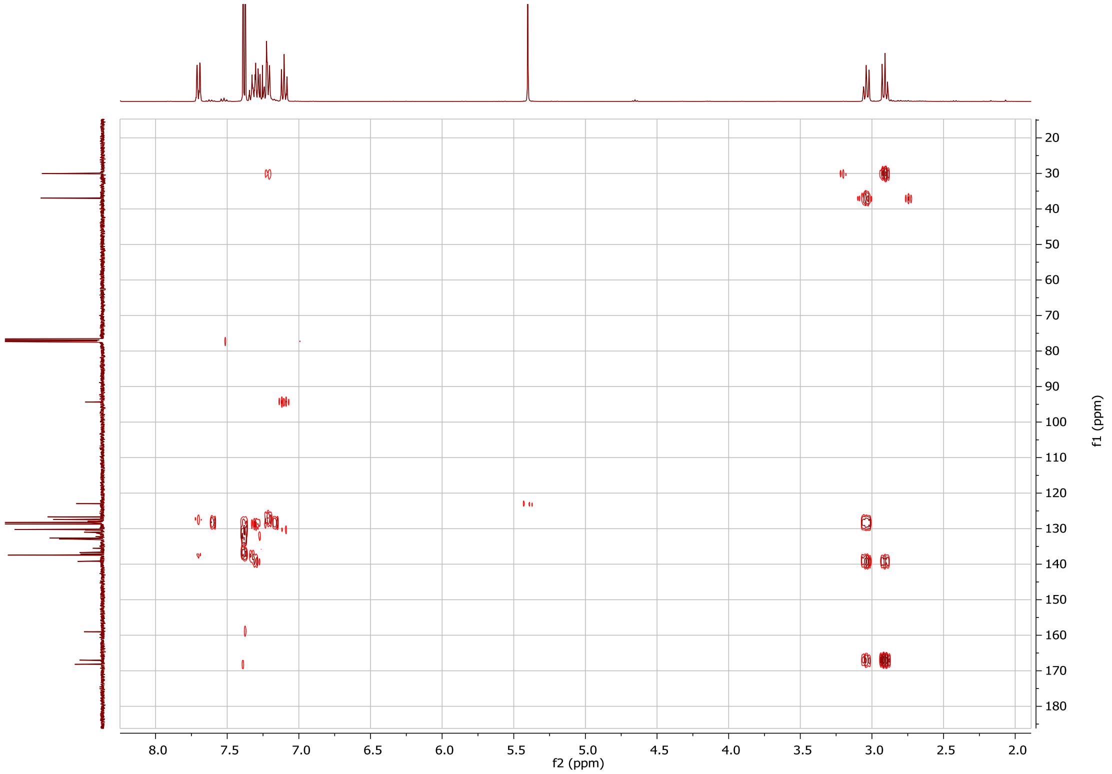


*Expansion:*

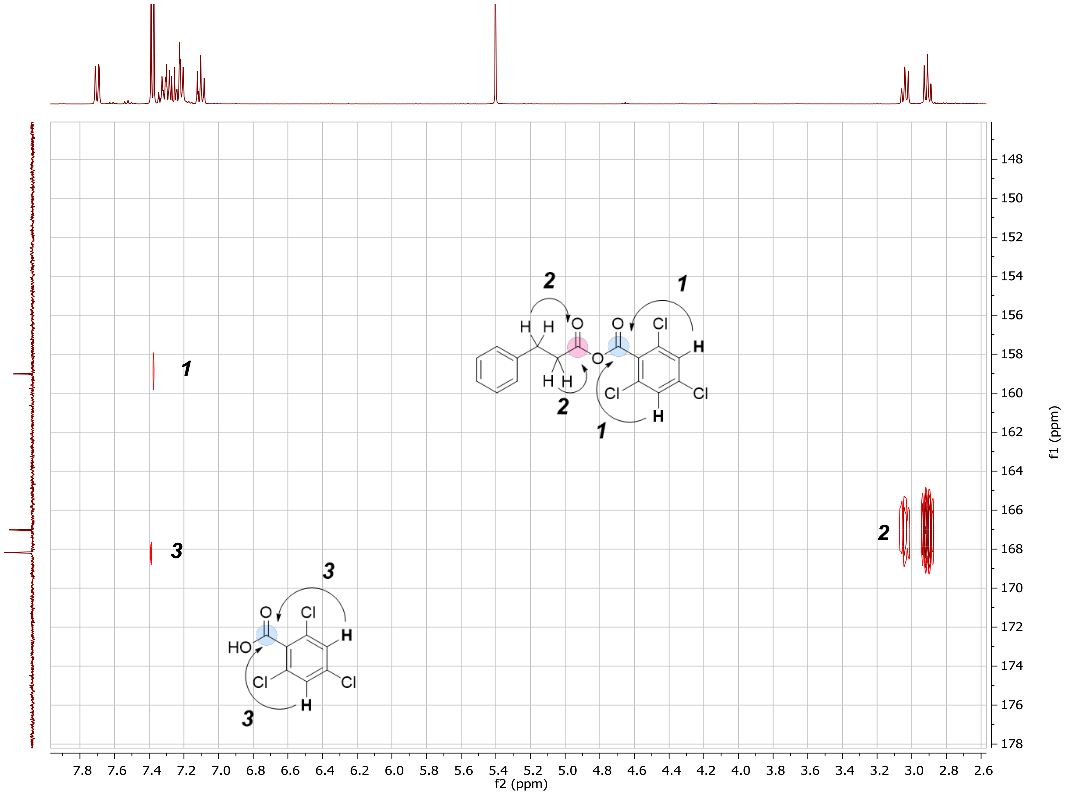


**Figure S3.** HMBC spectrum of the reaction mixture after completion of the reaction with the assignment of the key correlations.

## 2. Characterization of by-products

**3-Oxoundecyl acetate**: isolated in 12% yield in the reaction of 1-octylcyclopropanol **1b** with $PhI(OAc)_2$ without subsequent treatment of the reaction mixture with benzylamine (Table 2, Entry 1). IR (liquid film) $cm^{-1}$ 1746, 1719, 1238. $^1H$ NMR (400 MHz, $CDCl_3$) $\delta$ = 4.31 (t, $J$ = 6.3 Hz, 2H), 2.71 (t, $J$ = 6.3 Hz, 2H), 2.41 (t, $J$ = 7.4 Hz, 2H), 2.01 (s, 3H), 1.44-1.06 (m, 12H), 0.86 (t, $J$ = 6.8 Hz, 3H). $^{13}C$ NMR (100.6 MHz, $CDCl_3$) $\delta$ = 208.2, 171.0, 59.6, 43.4, 41.4, 31.9, 29.4, 29.3, 29.2, 23.7, 22.7, 20.9, 14.2.

**12-Hydroxydodecan-3-one (27):** was obtained in 81% yield in reaction of 1-(9-hydroxynonyl)cyclopropan-1-ol **1g** with scandium(III) triflate (Table 3, Entry 1) in a mixture of acetonitrile and tetrahydrofuran (17 : 1 by volume). M.p. 48.1-48.5 °C; IR (KBr) $cm^{-1}$ 3339, 3236, 1711, 1701. $^1H$ NMR (400 MHz, $CDCl_3$) $\delta$ = 3.61 (t, $J$ = 6.6 Hz, 2H), 2.44-2.33 (m, 4H), 1.68 (br.s, 1H), 1.60-1.48 (m, 4H), 1.37-1.19 (m, 10H), 1.02 (t, $J$ = 7.3 Hz, 3H). $^{13}C$ NMR (100.6 MHz, $CDCl_3$) $\delta$ = 212.0, 63.1, 42.5, 36.0, 32.9, 29.5, 29.4, 29.4, 29.4, 25.8, 24.0, 8.0. HRMS (ESI) *m*/*z* calcd for $C_{12}H_{25}O_2^+$ $[M+H]^+$ 201.1849, found 201.1881.

**1,12-Dioxacyclodocosane-2,13-dione (26a)**. White solid. IR (liquid film) $cm^{-1}$ 1735. $^1H$ NMR (400 MHz, $CDCl_3$) $\delta$ = 4.10 (t, $J$ = 5.8 Hz, 4H), 2.31 (t, $J$ = 6.9 Hz, 4H), 1.70-1.55 (m, 8H), 1.43-1.19 (m, 20H). $^{13}C$ NMR (100.6 MHz, $CDCl_3$) $\delta$ = 174.0, 64.2, 35.0, 29.5, 29.2, 29.2, 29.1, 28.8, 26.2, 25.6. HRMS (ESI) *m*/*z* calcd for $C_{20}H_{37}O_4^+$ $[M+H]^+$ 341.2686, found 341.2663. Spectral data are in accordance with those reported [1, 2].

**1,14-Dioxacyclohexacosane-2,15-dione (26b)**. White solid. IR (liquid film) $cm^{-1}$ 1735. $^1H$ NMR (400 MHz, $CDCl_3$) $\delta$ = 4.10 (t, $J$ = 5.8 Hz, 4H), 2.31 (t, $J$ = 7.1 Hz, 4H), 1.73-1.54 (m, 8H), 1.48-1.16 (m, 28H). $^{13}C$ NMR (100.6 MHz, $CDCl_3$) $\delta$ = 174.2, 64.3, 34.9, 29.7, 29.6, 29.6, 29.3, 29.1, 28.7, 26.2, 25.5. Spectral data are in accordance with those reported [1].

## 3. Preparation of cyclopropanols

Cyclopropanols **1a-d** have been prepared by Kulinkovich cyclopropanation of the corresponding carboxylic esters [3] and previously reported [4, 5]. Peptide cyclopropanol **1e** and bicyclic cyclopropanols **1f,i** were prepared by following the published protocols [6, 7]. Preparation of cyclopropanols **1g** and **1h** is described below.

**1-(9-Hydroxynonyl)cyclopropan-1-ol (1g)**

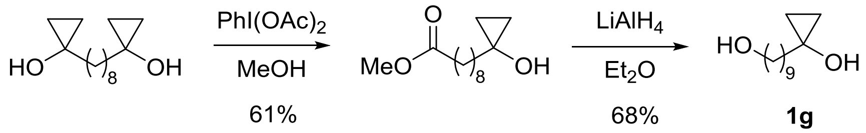


**1g**

To a solution of 1,1'-(octane-1,8-diyl)bis(cyclopropan-1-ol) (0.946 g, 4.2 mmol) in anhydrous methanol (53 mL) a solution of (diacetoxyiodo)benzene (1.348 g, 4.2 mmol) in methanol (19 mL) was added dropwise within 1 h. After reaction finished (TLC control) the reaction mixture was diluted with water (30 mL), extracted with dichloromethane (5 × 20 mL). Combined organic extracts were washed with brine (2 × 10 mL) and dried with $Na_2SO_4$. After removing the solvents under reduced pressure and purifying the residue by silica gel column chromatography (eluents: PE, then PE / EtOAc), methyl 9-(1-hydroxycyclopropyl)nonanoate was obtained as white solid (0.434 g, 61% yield, 74% conversion). M.p. 38.7-39.4 °C. IR (KBr) $cm^{-1}$ 3338, 3255, 1727. $^1H$ NMR (400 MHz, $CDCl_3$) $\delta$ = 3.66 (s, 3H), 2.29 (t, *J* = 7.5 Hz, 2H), 1.86 (br.s, 1H), 1.69-1.56 (m, 2H), 1.56-1.43 (m, 4H), 1.37-1.26 (m, 8H), 0.72 (dd, *J* = 6.2, 5.2 Hz, 2H), 0.42 (dd, *J* = 6.2, 5.2 Hz, 2H). $^{13}C$ NMR (100.6 MHz, $CDCl_3$) $\delta$ = 174.5, 56.0, 51.6, 38.4, 34.2, 29.7, 29.6, 29.3, 29.2, 26.0, 25.1, 13.6.

To a cooled to 0 °C suspension of lithium aluminium hydride (81 mg, 2.1 mmol) in anhydrous diethyl ether (7 mL) a solution of methyl 9-(1-hydroxycyclopropyl)nonanoate (433 mg, 1.9 mmol) in diethyl ether (5 mL) was added dropwise. After reaction finished (TLC control) the reaction mixture was quenched with an excess of ethyl acetate and washed with 10% sulfuric acid. The aqueous layer was extracted with diethyl ether (3 × 15 mL). The combined organic extracts were washed successively with saturated aqueous solutions of $NaHCO_3$, NaCl and dried with $Na_2SO_4$. After removal of the solvents under reduced pressure and purification of the residue by silica gel column chromatography, the target product was obtained as white solid (260 mg, 68% yield). M.p. 55.5-56.1 °C. IR (KBr) $cm^{-1}$ 3332, 3219. $^1H$ NMR (400 MHz, $CDCl_3$) $\delta$ = 3.63 (t, *J* = 6.6 Hz, 2H), 1.62-1.43 (m, 7H), 1.39-1.25 (m, 11H), 0.72 (dd, *J* = 6.4, 5.0 Hz, 2H), 0.43 (dd, *J* = 6.4, 5.0 Hz, 2H). $^{13}C$ NMR (100.6 MHz, $CDCl_3$) $\delta$ = 63.2, 56.0, 38.4, 32.9, 29.8, 29.7, 29.7, 29.5, 26.0, 25.9, 13.6.

**1-(11-Hydroxyundecyl)cyclopropan-1-ol (1h)**

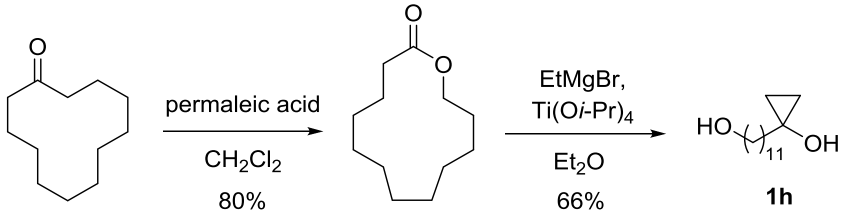


**1h**

The title compound was prepared in two steps: 1) oxidation of cyclododecanone with permaleic acid by the Baeyer-Villiger reaction to oxacyclotridecan-2-one (80% yield) according to the literature procedure [8]; 2) Kulinkovich hydroxycyclopropanation of oxacyclotridecan-2-one. The procedure of the Kulinkovich hydroxycyclopropanation is as follows: to a solution of oxacyclotridecan-2-one (2.23 g, 11.3 mmol) and titanium (IV) isopropoxide (3.20 g, 11.3 mmol) in anhydrous tetrahydrofuran (13 ml) a solution of

ethylmagnesium bromide (32.4 ml, 1.5 M) was added dropwise at room temperature. After completion of the reaction (TLC control), the mixture was treated with 10% sulfuric acid, the aqueous layer was separated and extracted with diethyl ether (3 × 30 ml). The combined organic extracts were washed sequentially with saturated aqueous $NaHCO_3$, NaCl and dried over $Na_2SO_4$. After evaporation of the solvents under reduced pressure and purification of the residue by recrystallization from benzene target product (1.69 g, 66% yield) was obtained. White solid, m.p. 66.7-67.6°C (benzene). IR (KBr) $cm^{-1}$ 3326, 3219. $^1H$ NMR (400 MHz, $CDCl_3$) $\delta$ = 3.62 (t, $J$ = 6.3 Hz, 2H), 1.64-1.42 (m, 7H), 1.40-1.18 (m, 15H), 0.78-0.63 (m, 2H), 0.47-0.37 (m, 2H). $^{13}C$ NMR (100.6 MHz, $CDCl_3$) $\delta$ = 63.2, 55.9, 38.4, 32.9, 29.8, 29.7, 29.7, 29.5, 26.0, 25.8, 13.6.

# 4. Copies of $^1$H and $^{13}$C NMR spectra

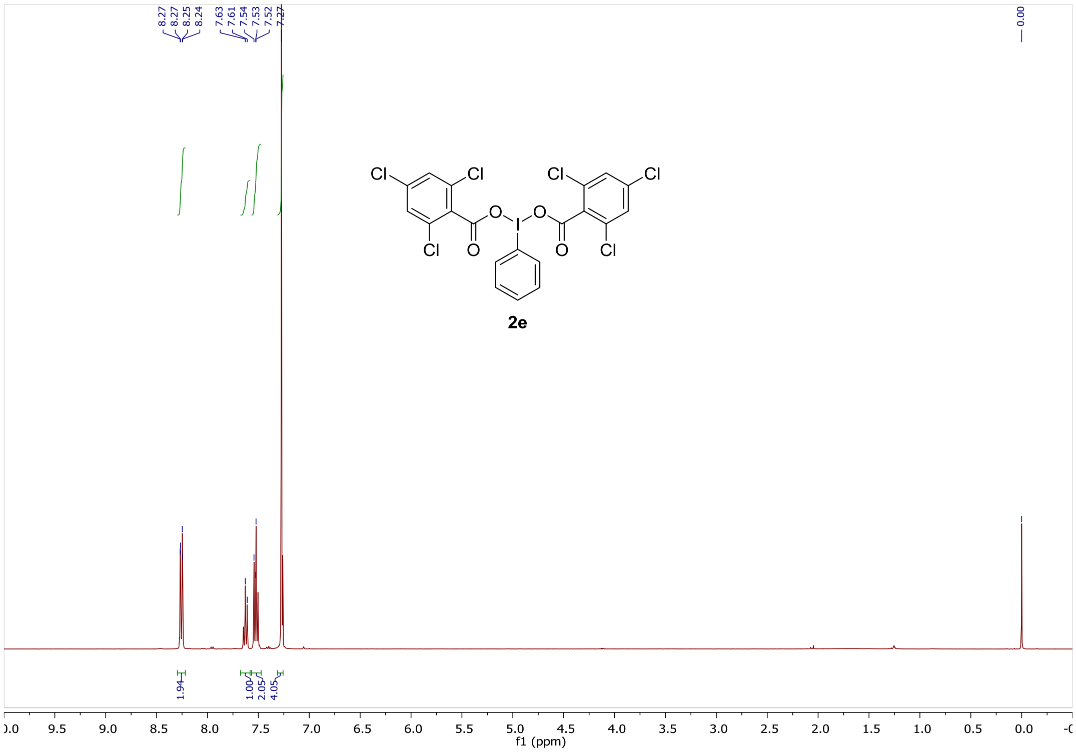


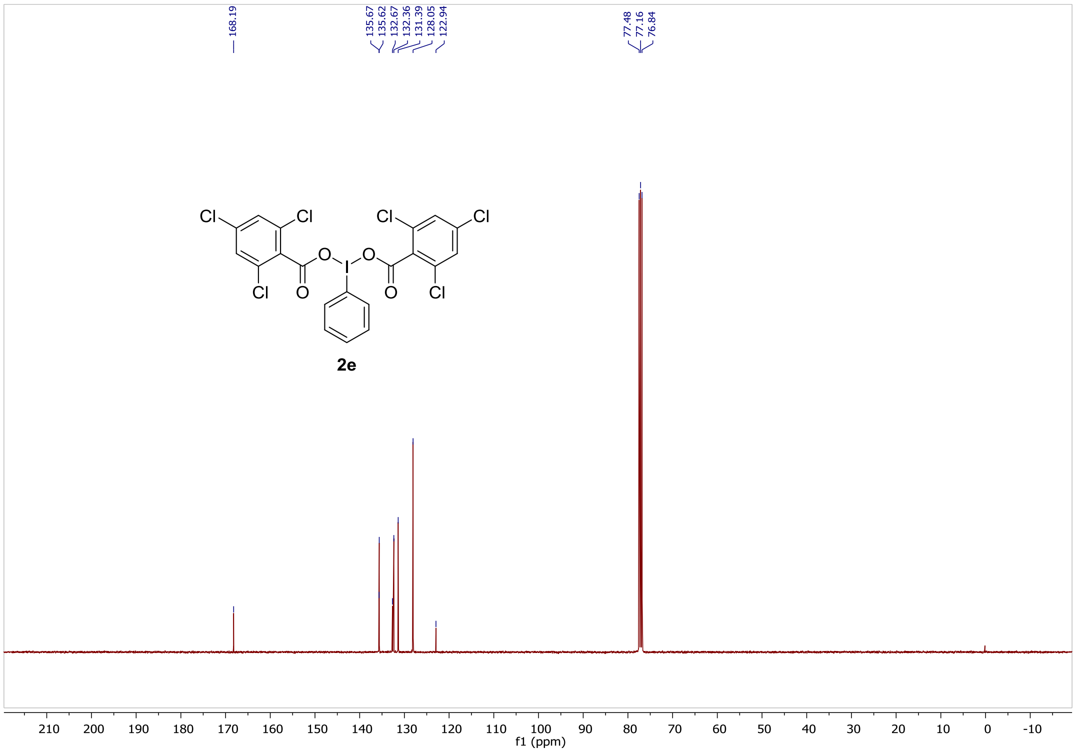

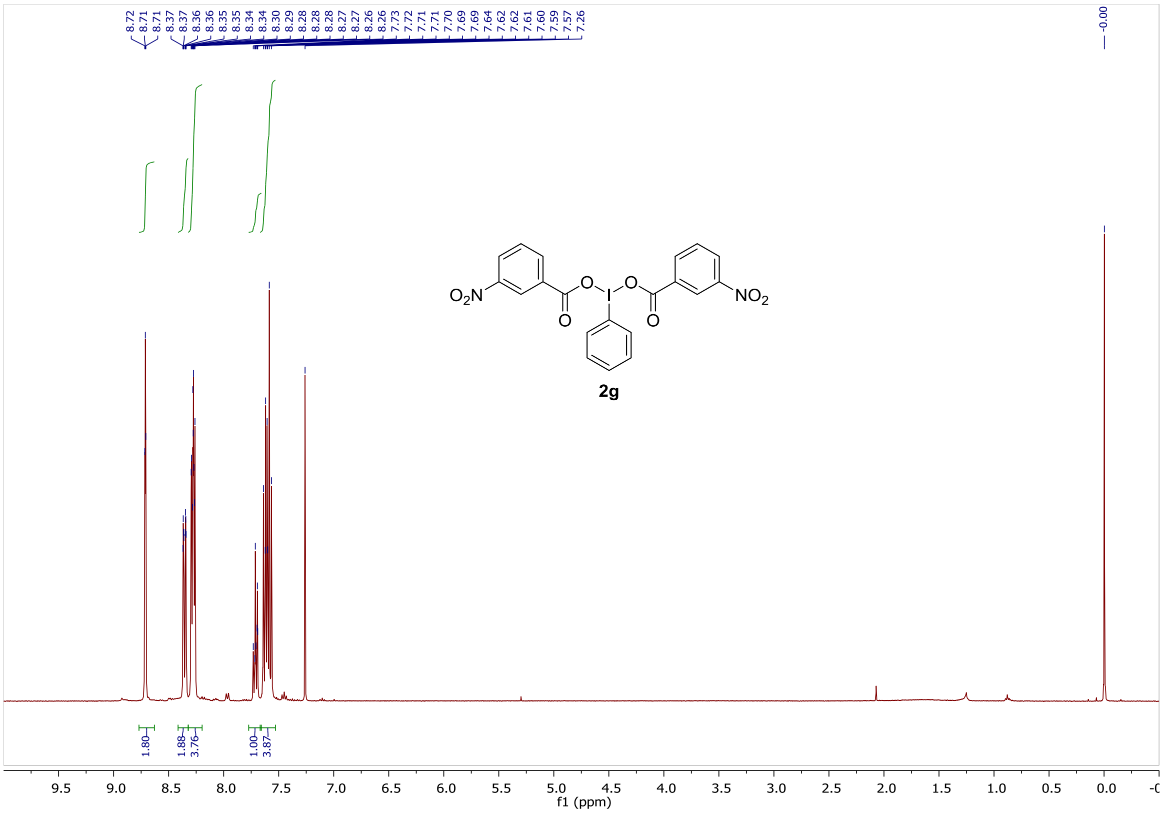
8.72
8.71
8.71
8.37
8.36
8.36
8.35
8.35
8.34
8.34
8.30
8.29
8.28
8.28
8.27
8.26
8.26
7.73
7.72
7.71
7.71
7.70
7.69
7.69
7.64
7.62
7.62
7.61
7.60
7.59
7.57
7.26
-0.00
$O_2N$
O
I
O
$NO_2$
O
O
2g
1.80
1.88
3.76
1.00
3.87
f1 (ppm)

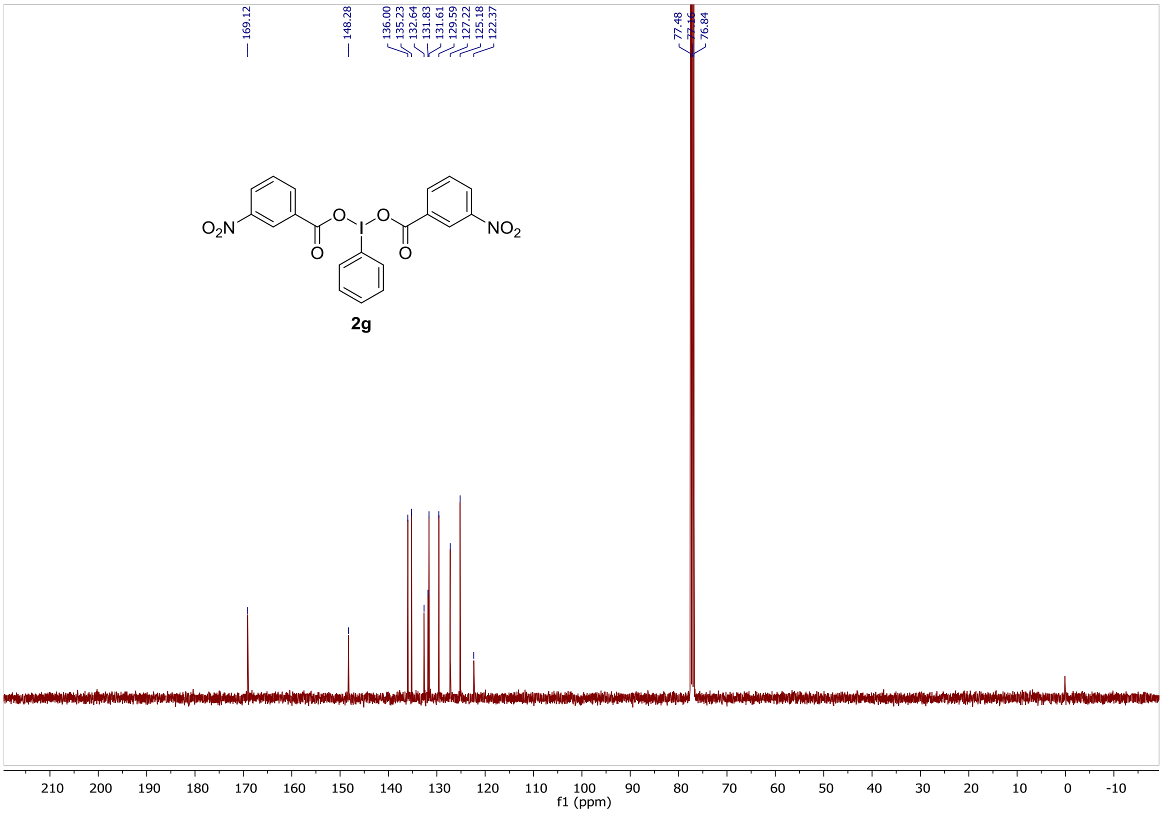
169.12
148.28
136.00
135.23
132.64
131.83
131.61
129.59
127.22
125.18
122.37
77.48
77.16
76.84
$O_2N$
O
I
O
$NO_2$
O
O
2g
f1 (ppm)

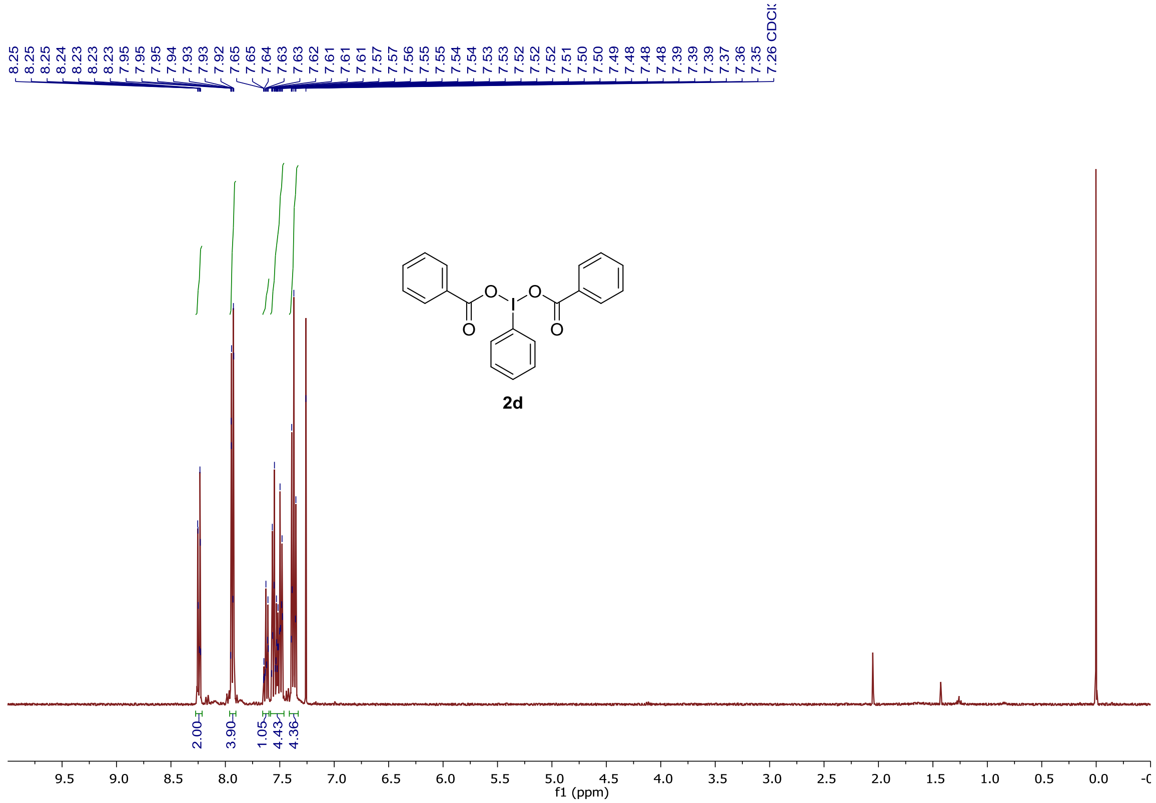
8.25
8.25
8.25
8.24
8.23
8.23
8.23
7.95
7.95
7.95
7.94
7.93
7.93
7.92
7.65
7.65
7.64
7.63
7.63
7.62
7.61
7.61
7.61
7.57
7.57
7.56
7.55
7.55
7.54
7.54
7.53
7.53
7.52
7.52
7.51
7.50
7.50
7.49
7.48
7.48
7.48
7.39
7.39
7.39
7.37
7.36
7.35
7.26 CDCl3
2d
2.00
3.90
1.05
4.43
4.36
9.5
9.0
8.5
8.0
7.5
7.0
6.5
6.0
5.5
5.0
4.5
4.0
3.5
3.0
2.5
2.0
1.5
1.0
0.5
0.0
f1 (ppm)

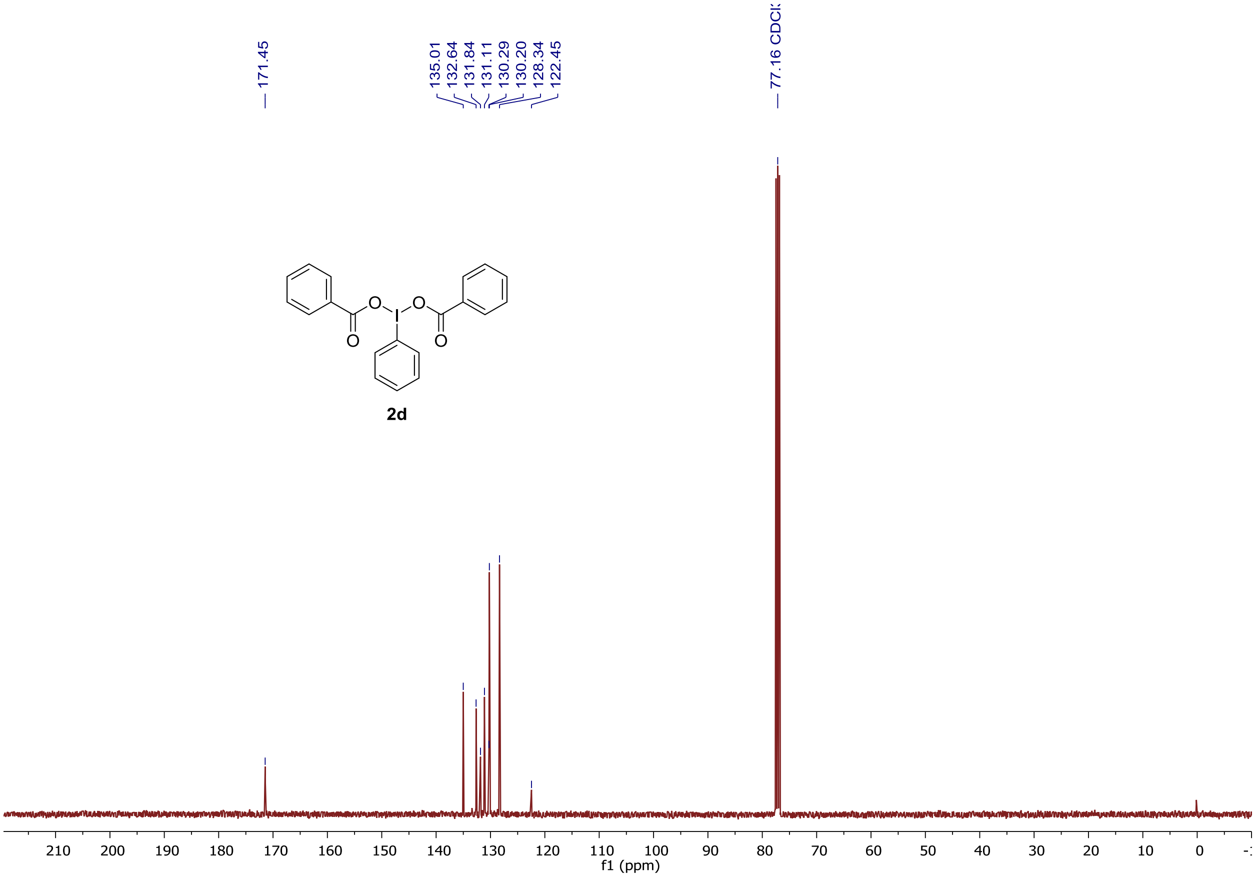
171.45
135.01
132.64
131.84
131.11
130.29
130.20
128.34
122.45
77.16 CDCl3
2d
210
200
190
180
170
160
150
140
130
120
110
100
90
80
70
60
50
40
30
20
10
0
f1 (ppm)

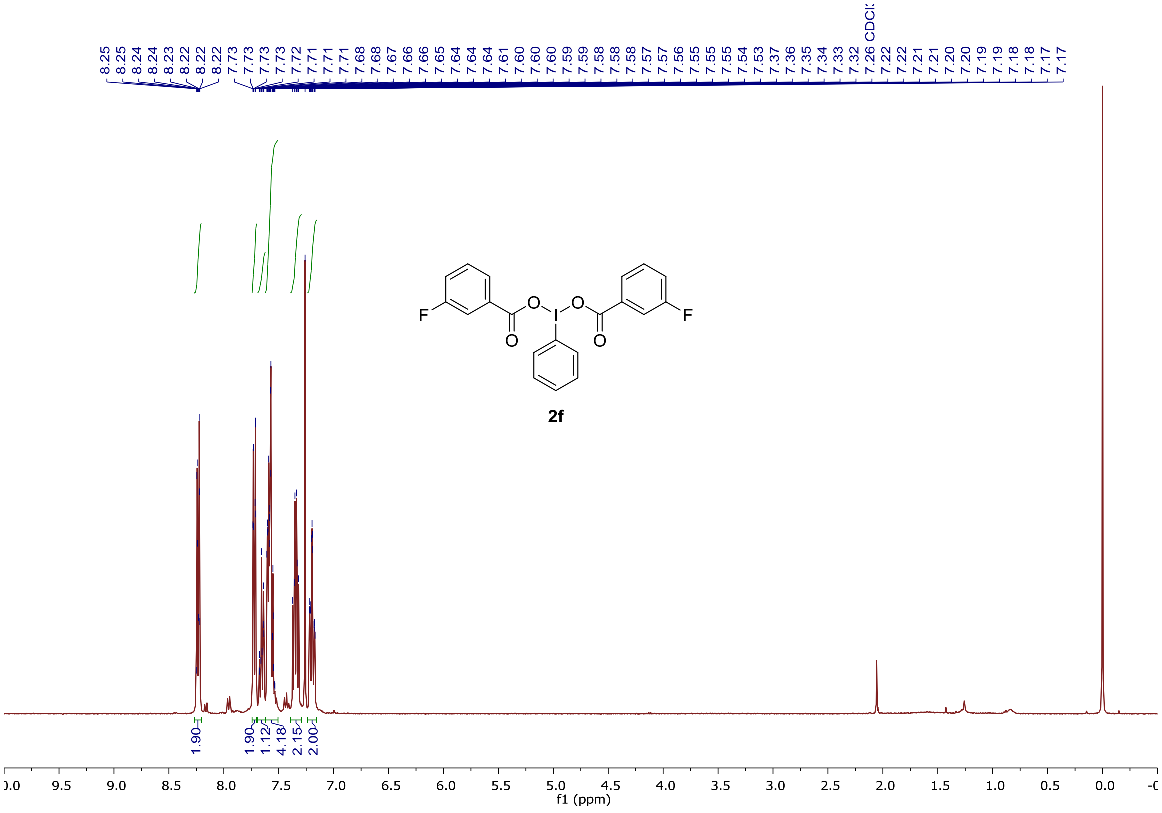
8.25 8.25 8.24 8.24 8.23 8.22 8.22 8.22 7.73 7.73 7.73 7.73 7.72 7.71 7.71 7.71 7.68 7.68 7.67 7.66 7.66 7.65 7.64 7.64 7.64 7.61 7.60 7.60 7.59 7.59 7.58 7.58 7.58 7.57 7.57 7.56 7.55 7.55 7.55 7.54 7.53 7.37 7.36 7.35 7.34 7.33 7.32 7.26 CDCl3 7.22 7.22 7.21 7.21 7.20 7.19 7.19 7.18 7.17 7.17
F
O
I
O
F
O
O
2f
1.90
1.90
1.12
4.18
2.15
2.00
f1 (ppm)

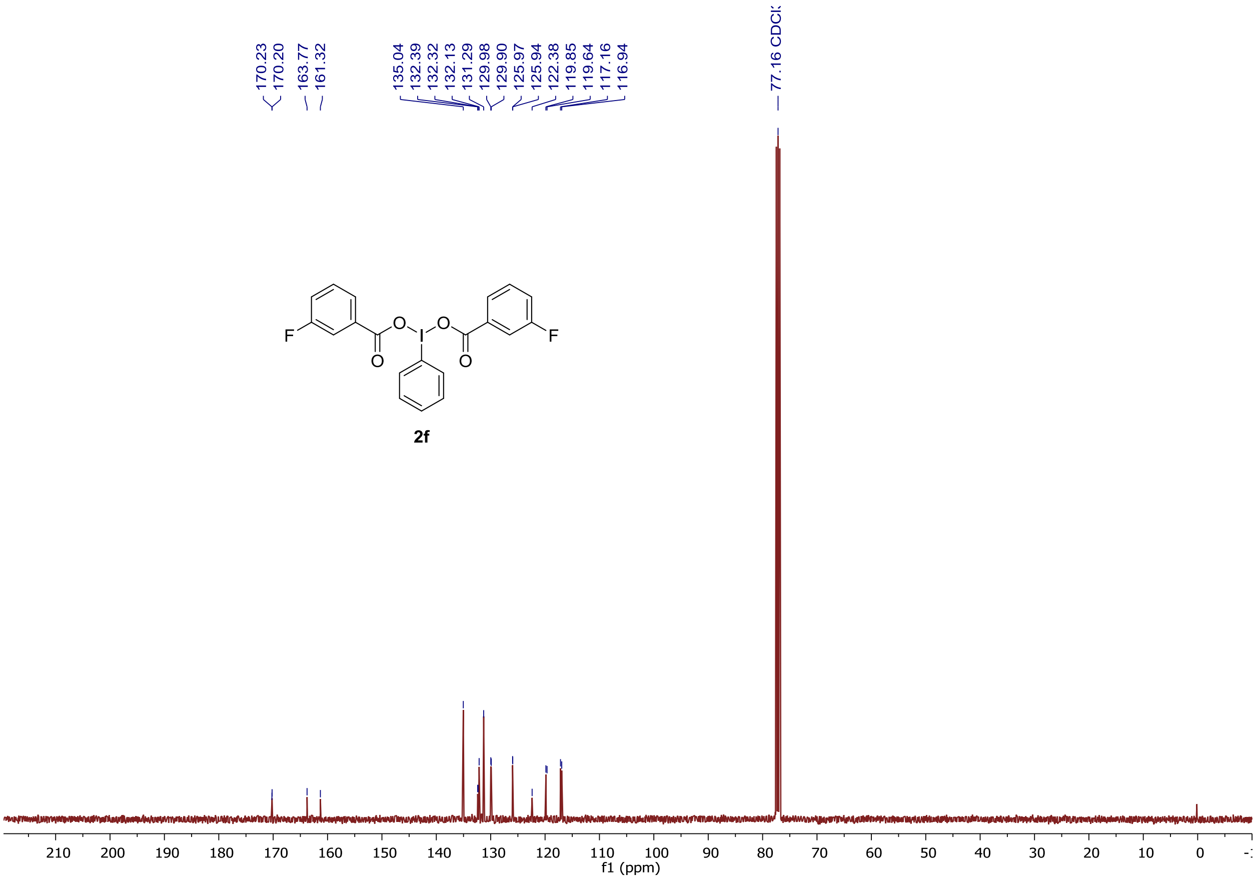
170.23 170.20 163.77 161.32
135.04 132.39 132.32 132.13 131.29 129.98 129.90 125.97 125.94 122.38 119.85 119.64 117.16 116.94
77.16 CDCl3
F
O
I
O
F
O
O
2f
f1 (ppm)

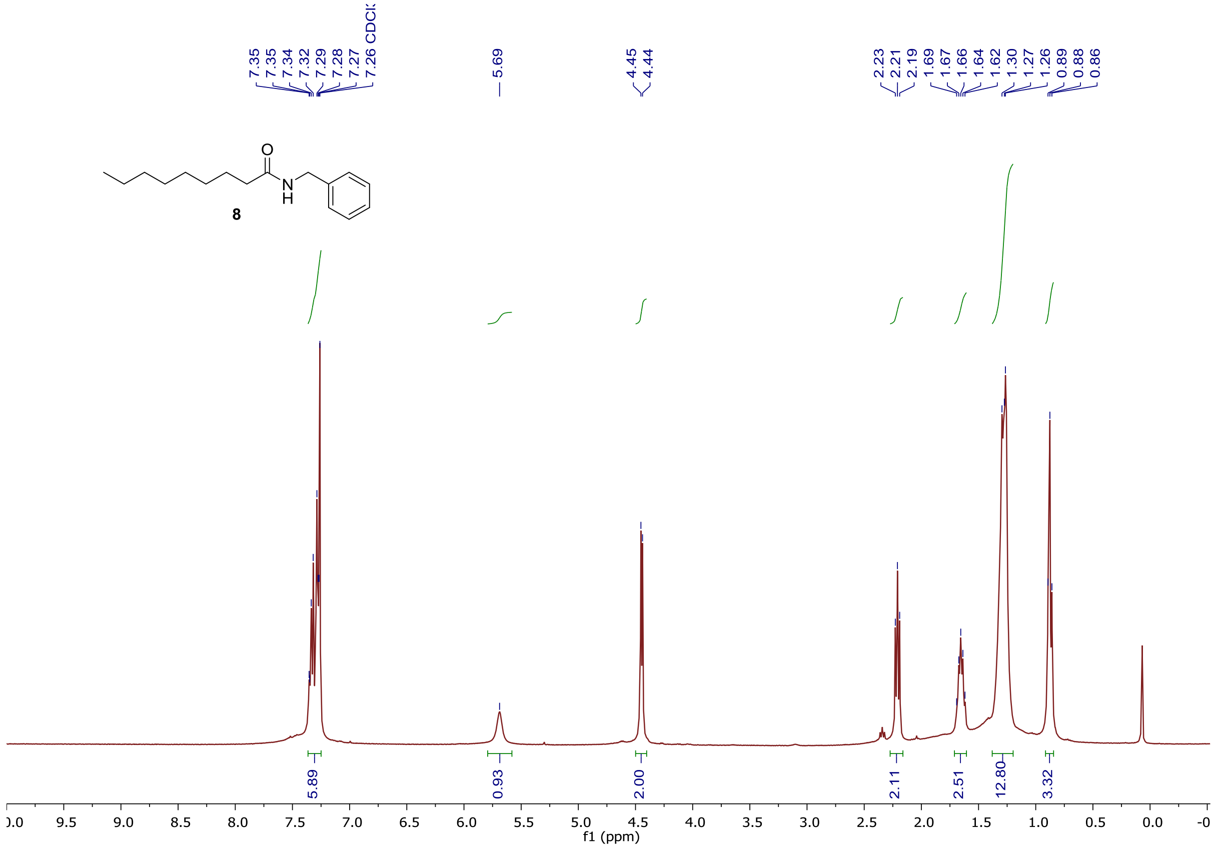
7.35
7.35
7.34
7.32
7.29
7.28
7.27
7.26 CDCl3
5.69
4.45
4.44
2.23
2.21
2.19
1.69
1.67
1.66
1.64
1.62
1.30
1.27
1.26
0.89
0.88
0.86
8
5.89
0.93
2.00
2.11
2.51
12.80
3.32
f1 (ppm)

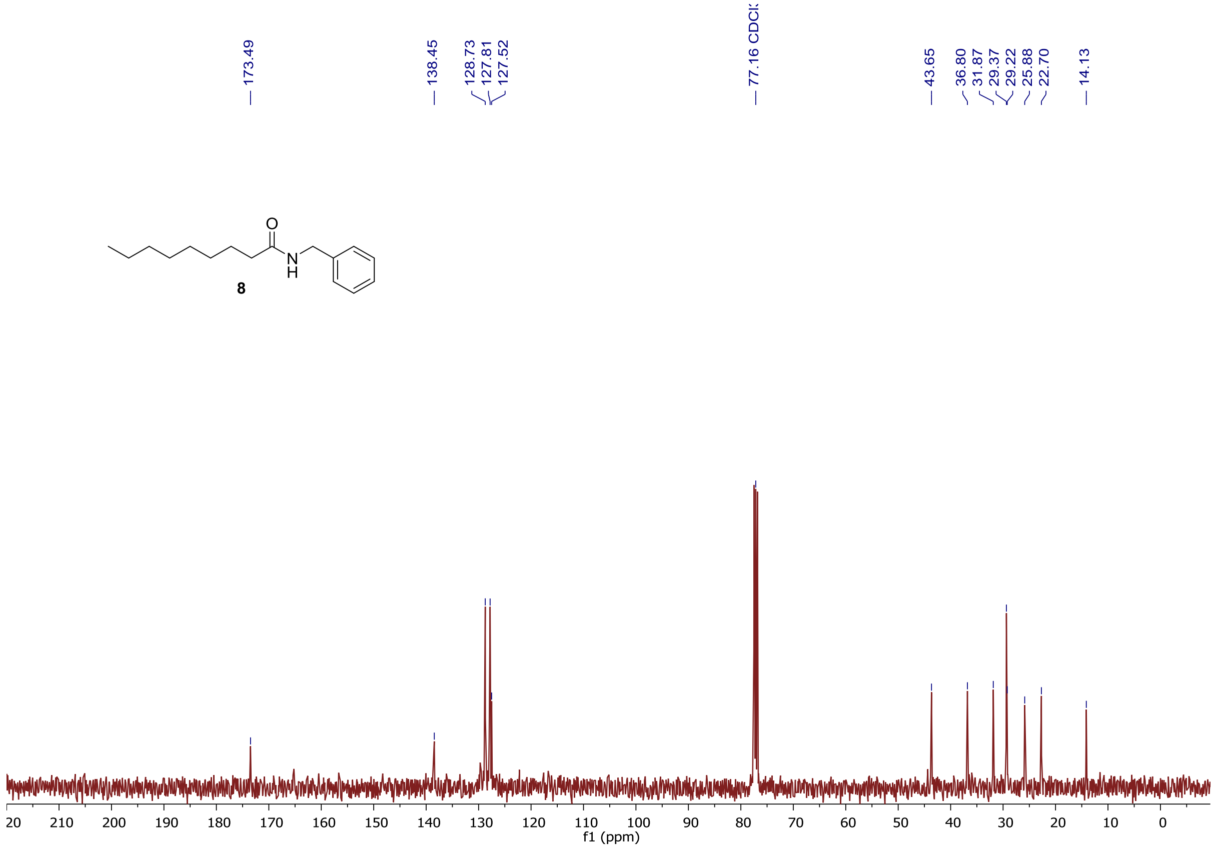
173.49
138.45
128.73
127.81
127.52
77.16 CDCl3
43.65
36.80
31.87
29.37
29.22
25.88
22.70
14.13
8
f1 (ppm)

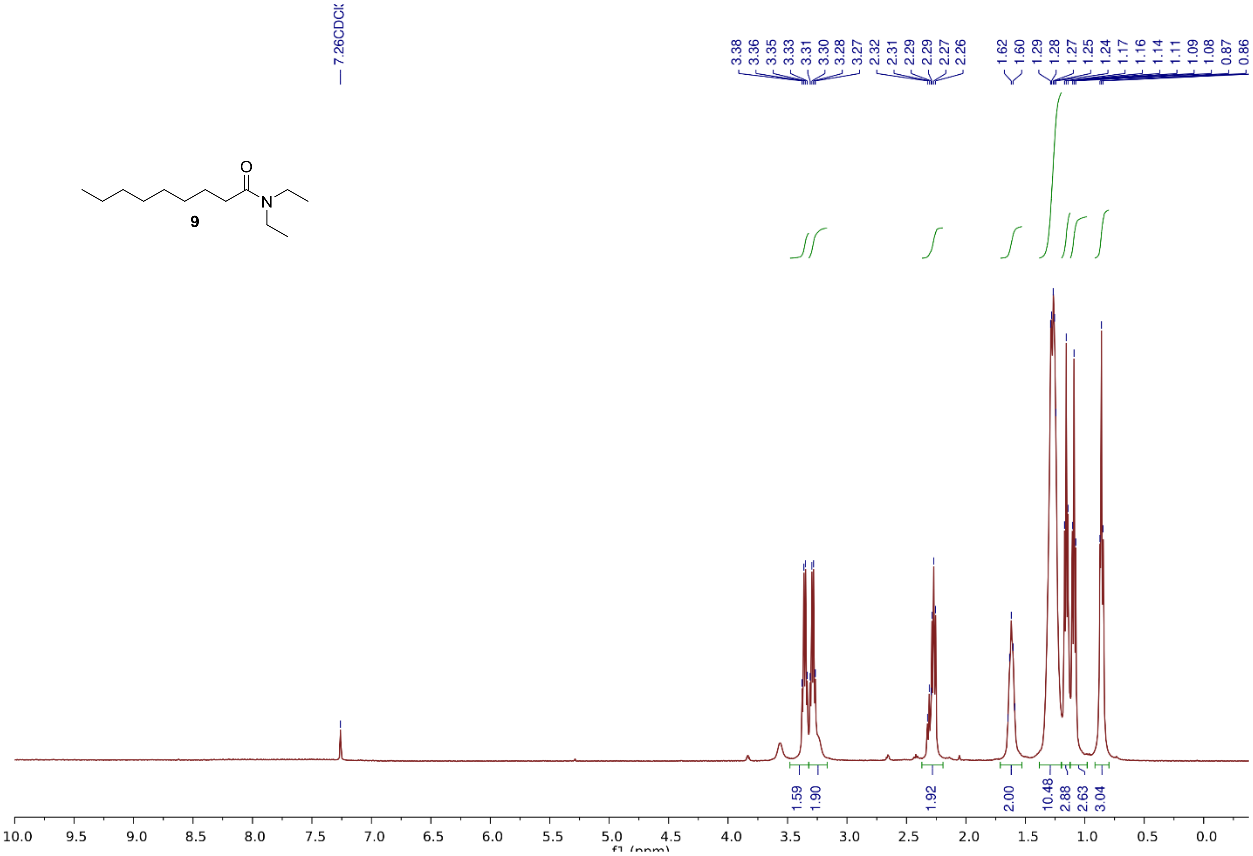
9
f1 (ppm)

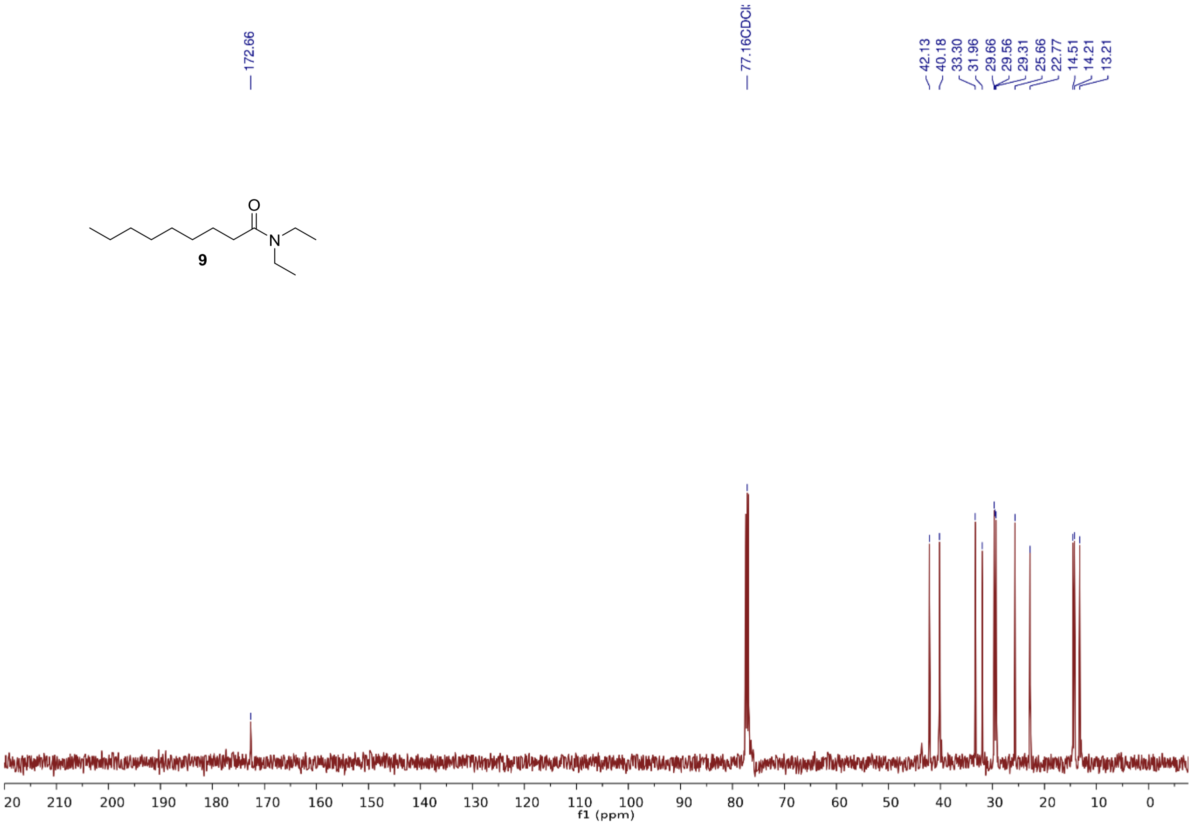
9
f1 (ppm)

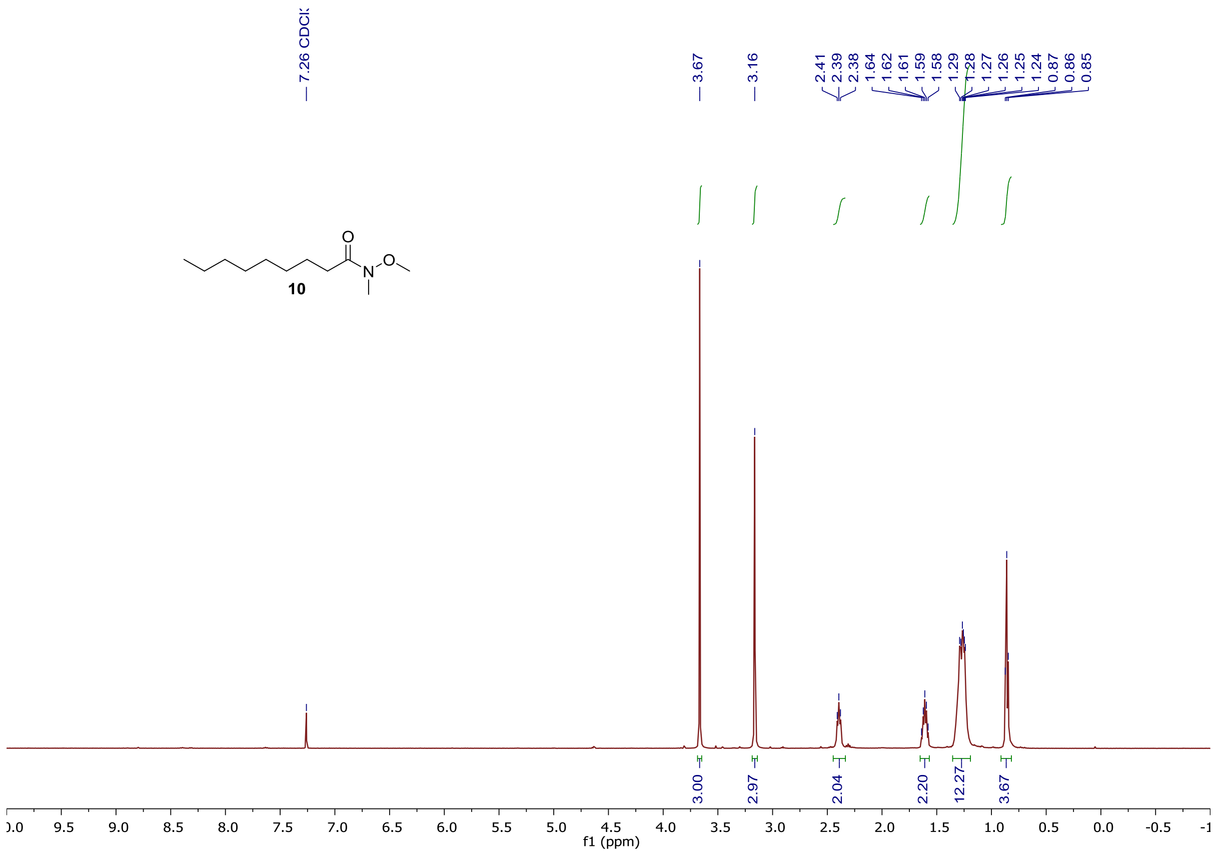
7.26 CDCl3
3.67
3.16
2.41
2.39
2.38
1.64
1.62
1.61
1.59
1.58
1.29
1.28
1.27
1.26
1.25
1.24
0.87
0.86
0.85
10
3.00
2.97
2.04
2.20
12.27
3.67
f1 (ppm)

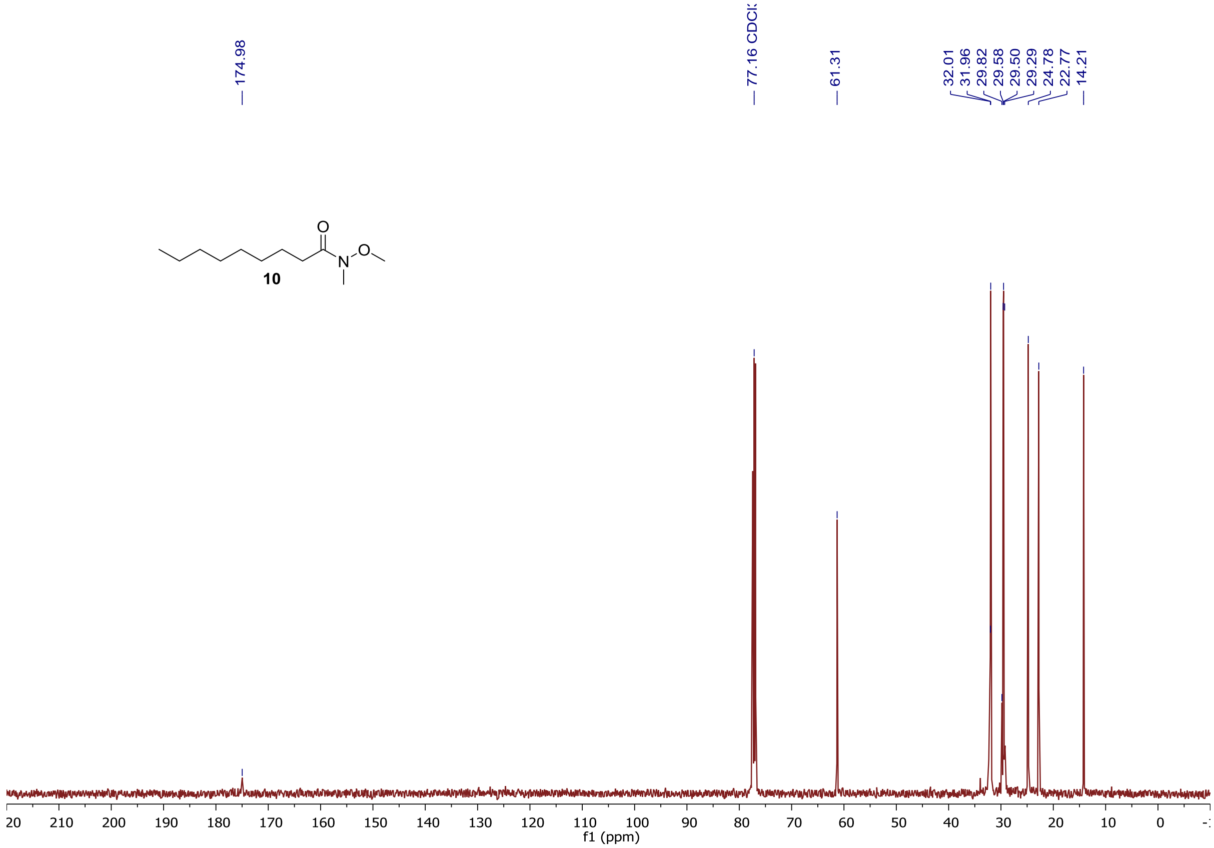
174.98
77.16 CDCl3
61.31
32.01
31.96
29.82
29.58
29.50
29.29
24.78
22.77
14.21
10
f1 (ppm)

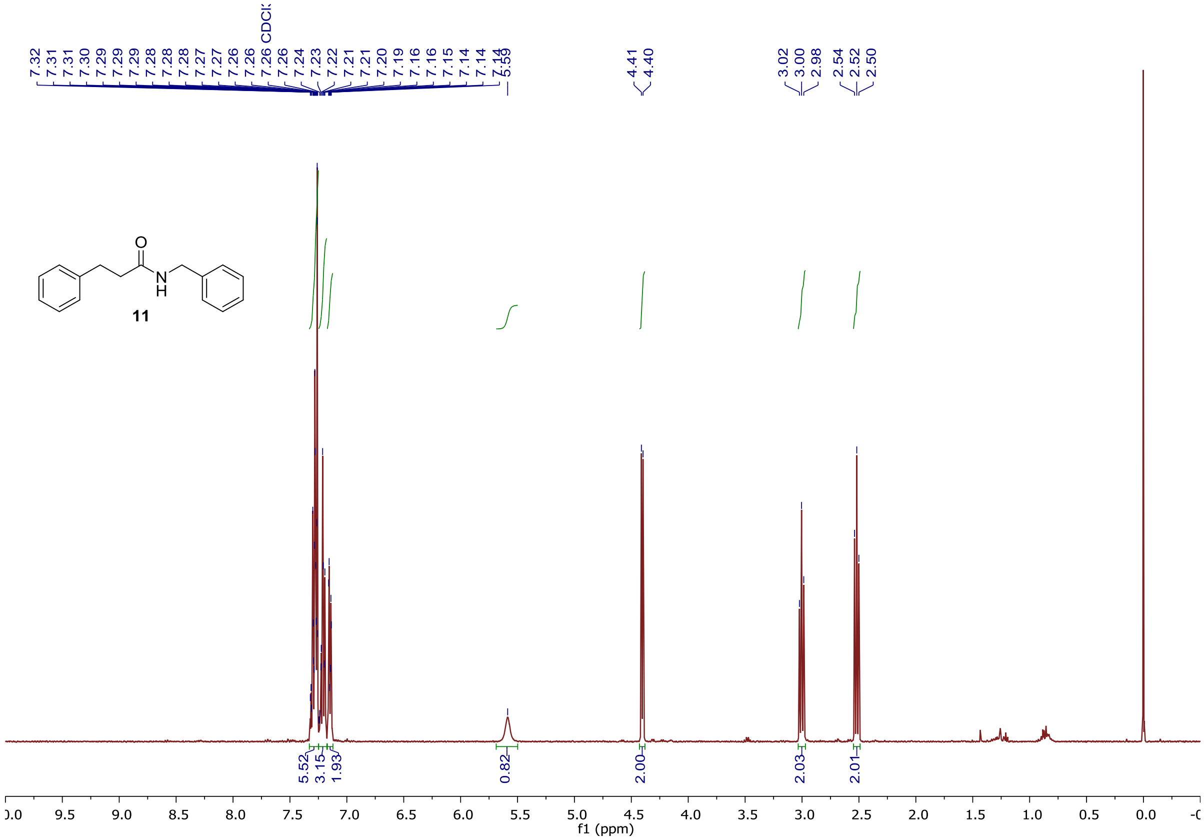
7.32
7.31
7.30
7.29
7.29
7.28
7.28
7.28
7.27
7.27
7.26
7.26
7.26 CDCl3
7.26
7.24
7.23
7.22
7.21
7.21
7.20
7.19
7.16
7.16
7.15
7.14
7.14
7.14
5.59
4.41
4.40
3.02
3.00
2.98
2.54
2.52
2.50
O
N
H
11
5.52
3.15
1.93
0.82
2.00
2.03
2.01
f1 (ppm)

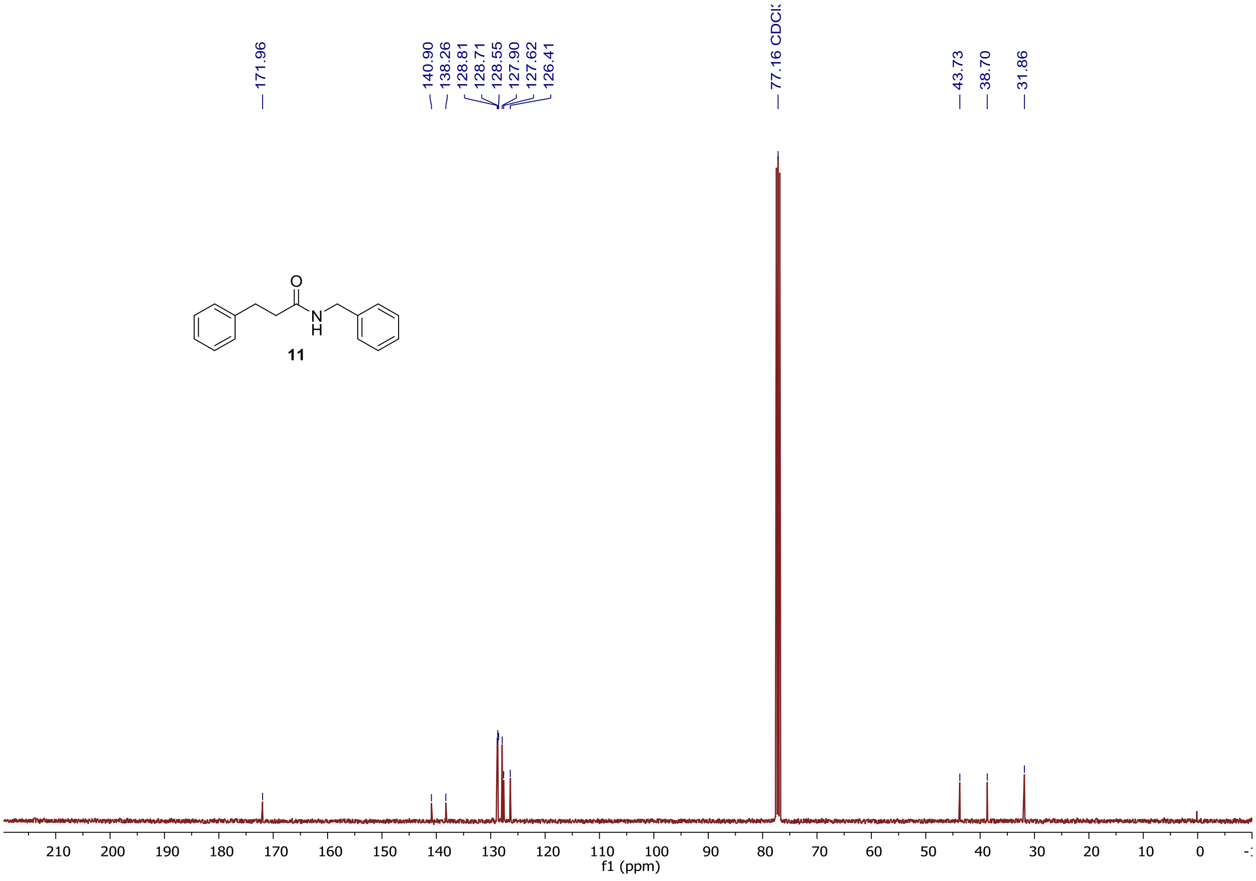
171.96
140.90
138.26
128.81
128.71
128.55
127.90
127.62
126.41
77.16 CDCl3
43.73
38.70
31.86
O
N
H
11
f1 (ppm)

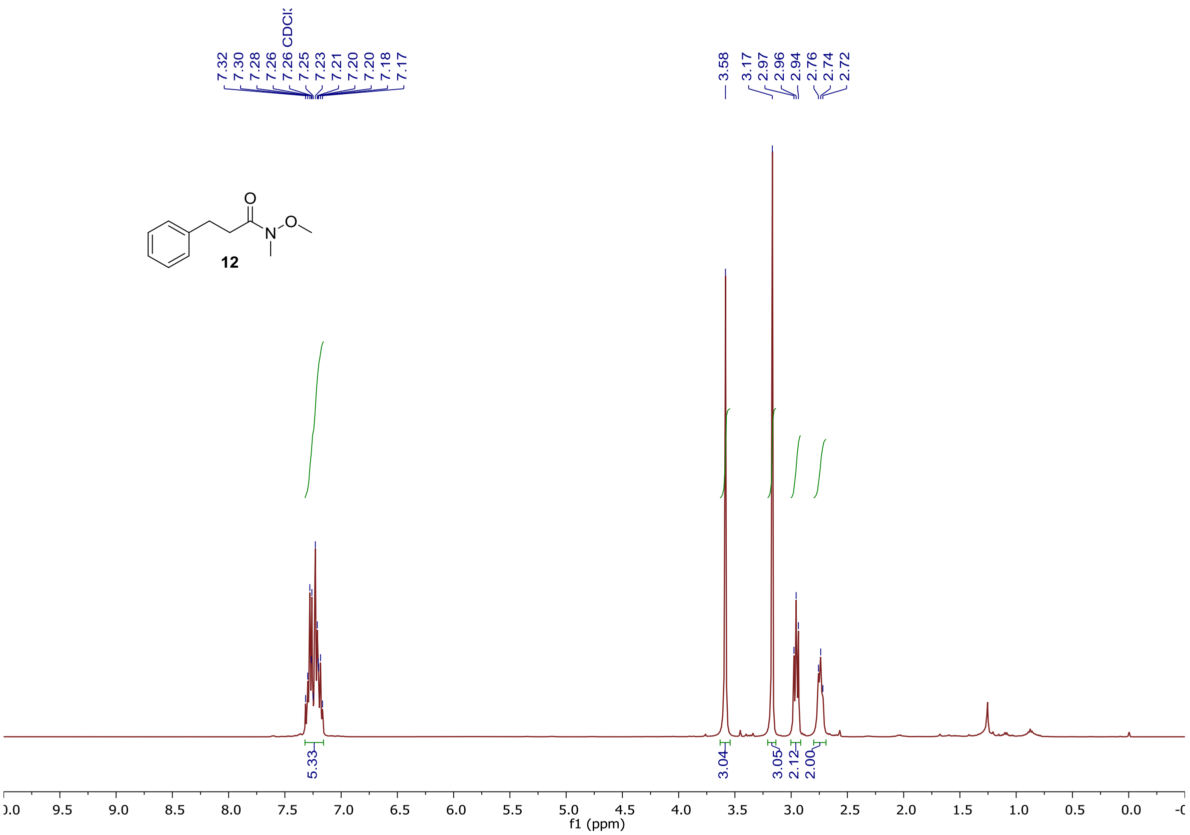
7.32
7.30
7.28
7.26
7.26 CDCl3
7.25
7.23
7.21
7.20
7.20
7.18
7.17
3.58
3.17
2.97
2.96
2.94
2.76
2.74
2.72
12
5.33
3.04
3.05
2.12
2.00
f1 (ppm)

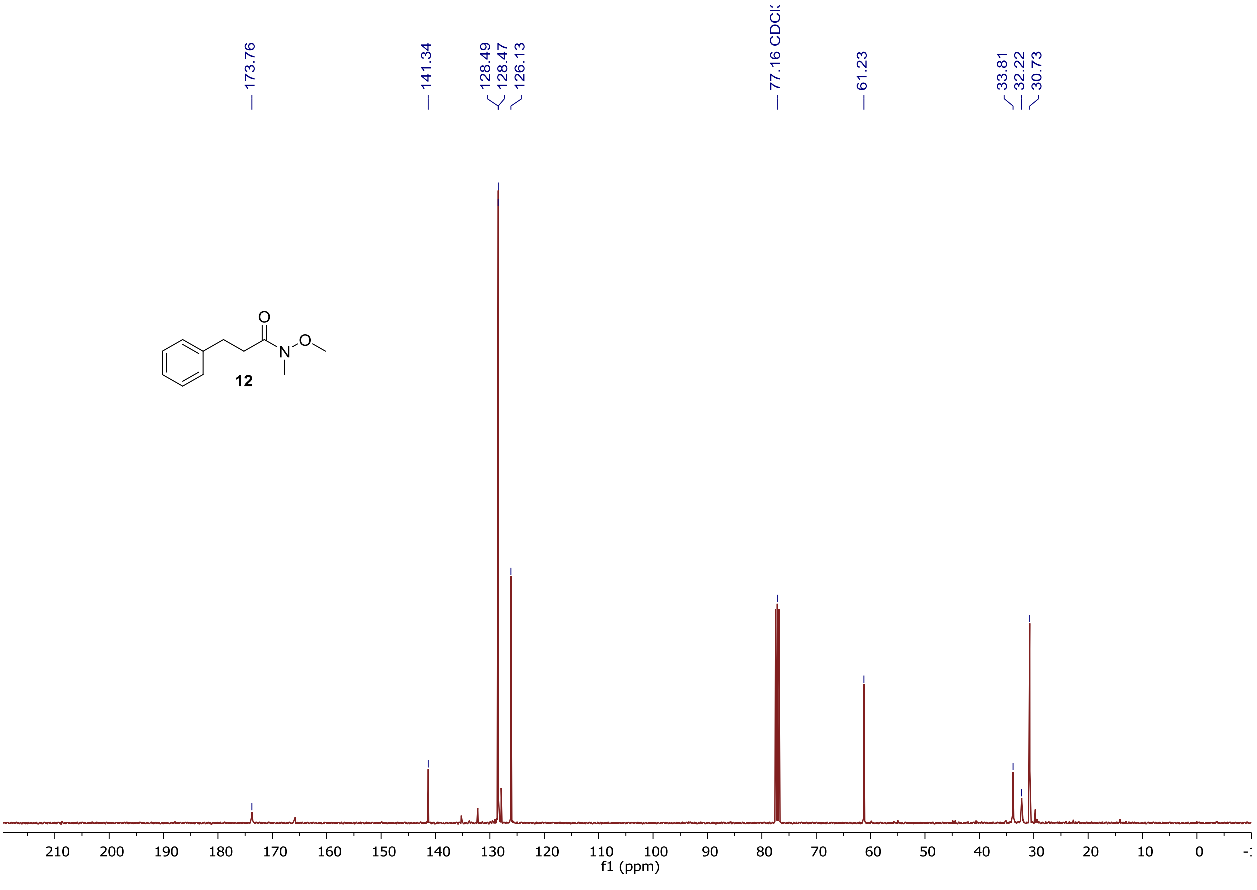
173.76
141.34
128.49
128.47
126.13
77.16 CDCl3
61.23
33.81
32.22
30.73
12
f1 (ppm)

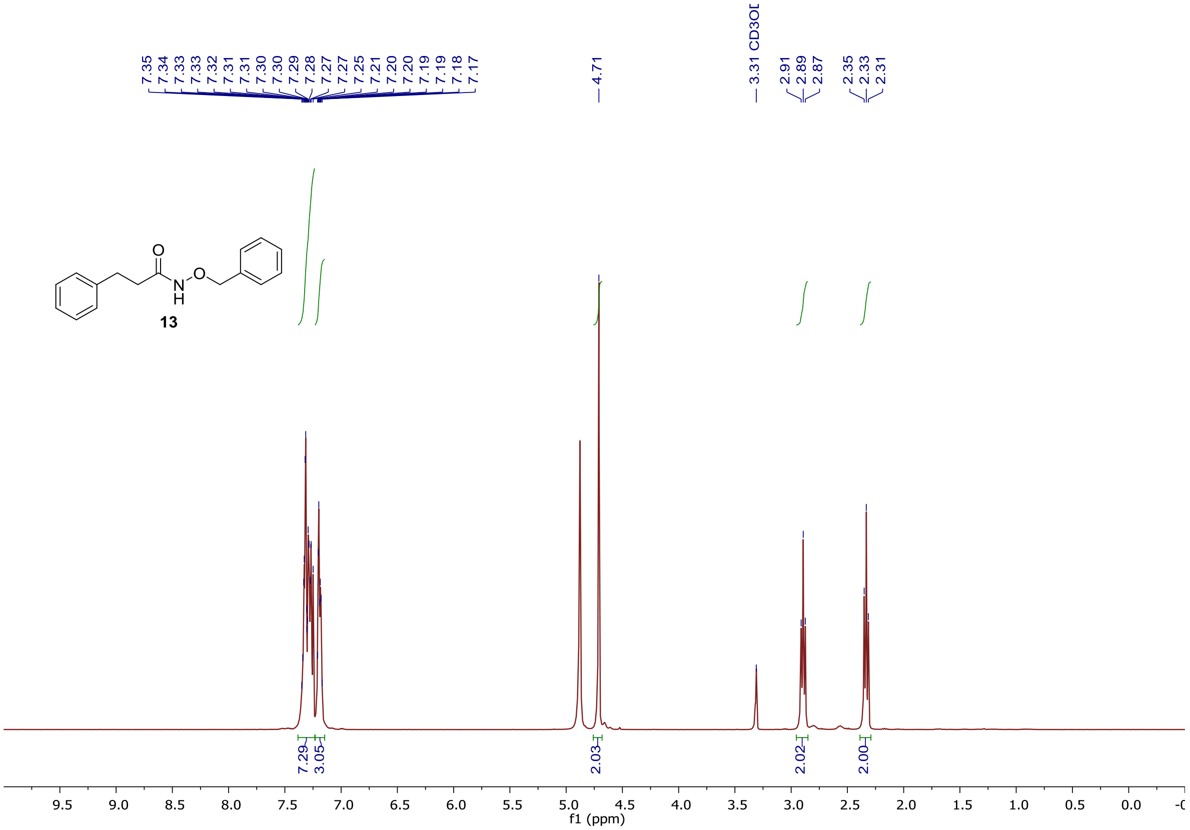
7.35
7.34
7.33
7.33
7.32
7.31
7.31
7.30
7.30
7.29
7.28
7.27
7.27
7.25
7.21
7.20
7.20
7.19
7.19
7.18
7.17
4.71
3.31 CD3OD
2.91
2.89
2.87
2.35
2.33
2.31
13
7.29
3.05
2.03
2.02
2.00
9.5 9.0 8.5 8.0 7.5 7.0 6.5 6.0 5.5 5.0 4.5 4.0 3.5 3.0 2.5 2.0 1.5 1.0 0.5 0.0
f1 (ppm)

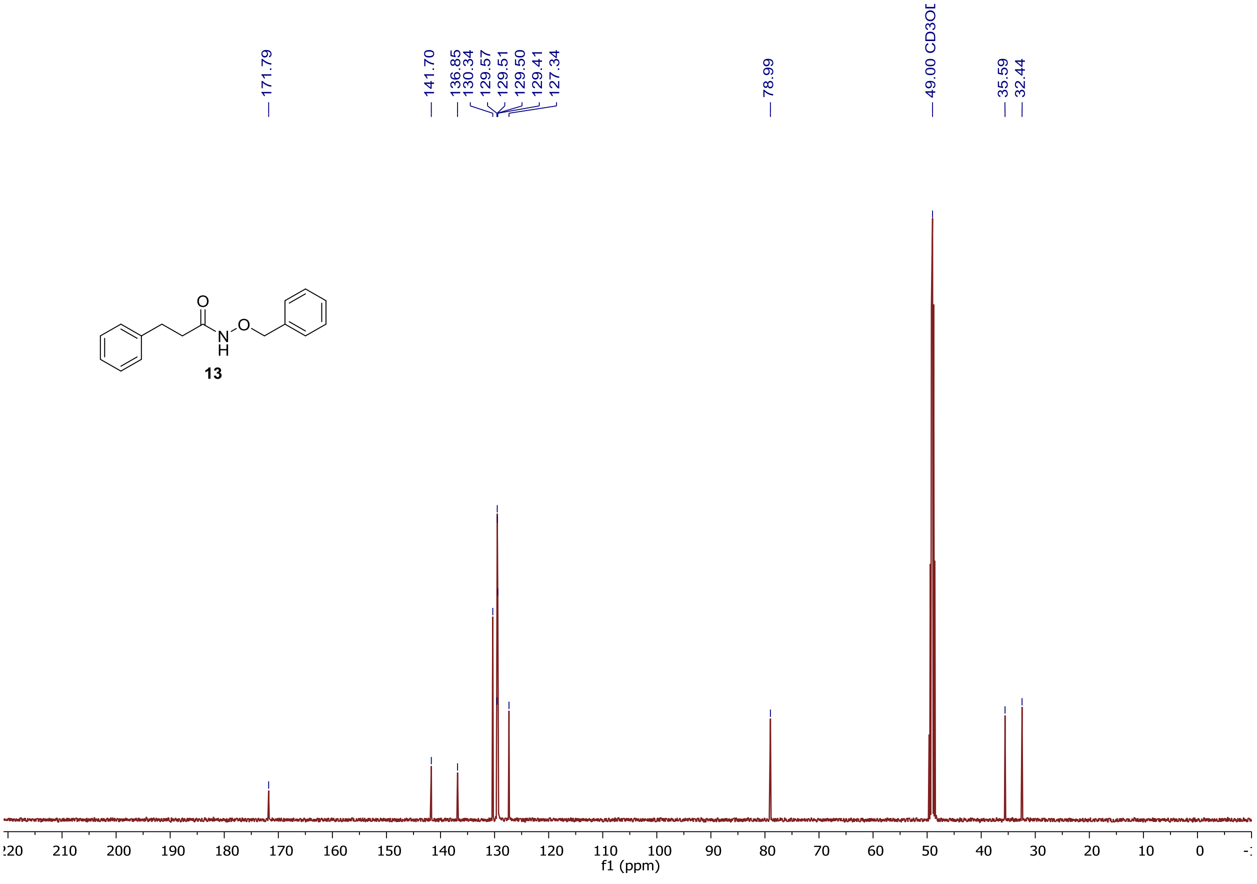
171.79
141.70
136.85
130.34
129.57
129.51
129.50
129.41
127.34
78.99
49.00 CD3OD
35.59
32.44
13
210 200 190 180 170 160 150 140 130 120 110 100 90 80 70 60 50 40 30 20 10 0
f1 (ppm)

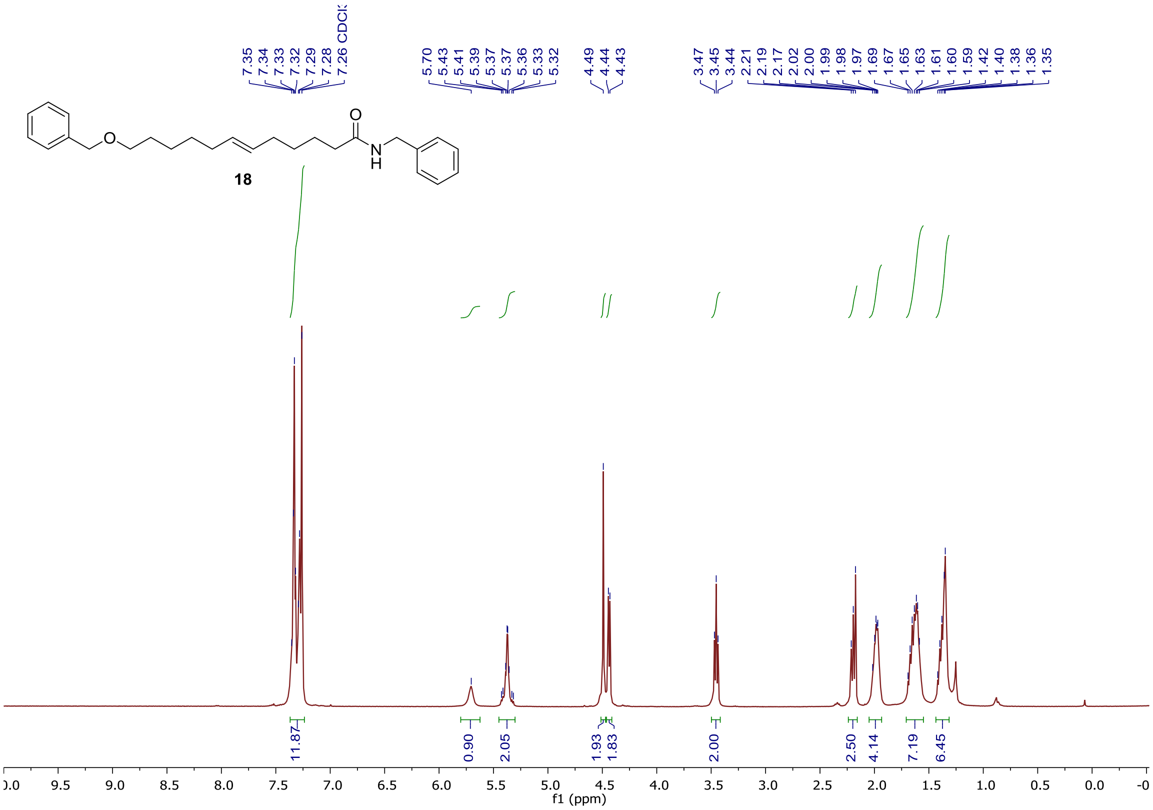
18
11.87
0.90
2.05
1.93
1.83
2.00
2.50
4.14
7.19
6.45
f1 (ppm)

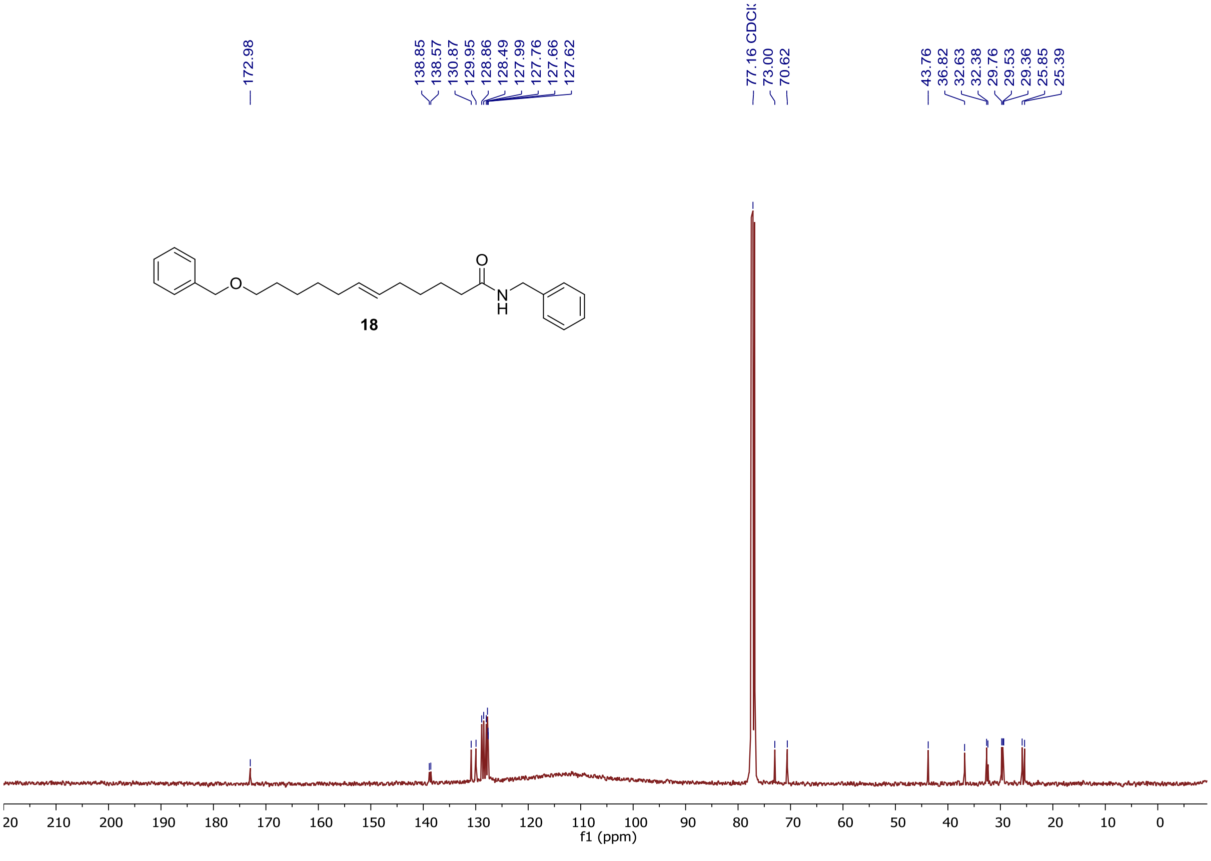
18
172.98
77.16 CDCl3
73.00
70.62
43.76
36.82
f1 (ppm)

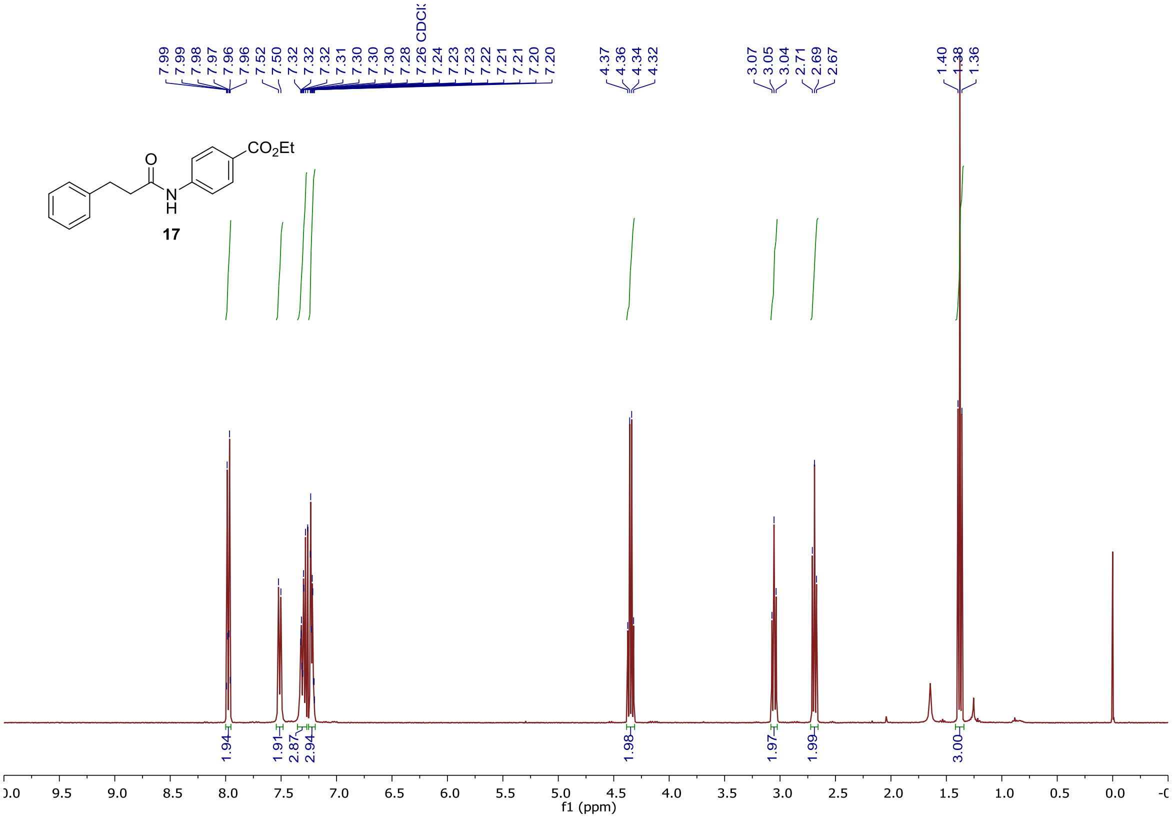
7.99 7.99 7.98 7.97 7.96 7.96 7.52 7.50 7.32 7.32 7.32 7.32 7.31 7.30 7.30 7.30 7.28 7.26 CDCl3 7.24 7.23 7.23 7.22 7.21 7.21 7.20 7.20
4.37 4.36 4.34 4.32
3.07 3.05 3.04 2.71 2.69 2.67
1.40 1.38 1.36
CO2Et
O
N
H
17
1.94
1.91
2.87
2.94
1.98
1.97
1.99
3.00
f1 (ppm)

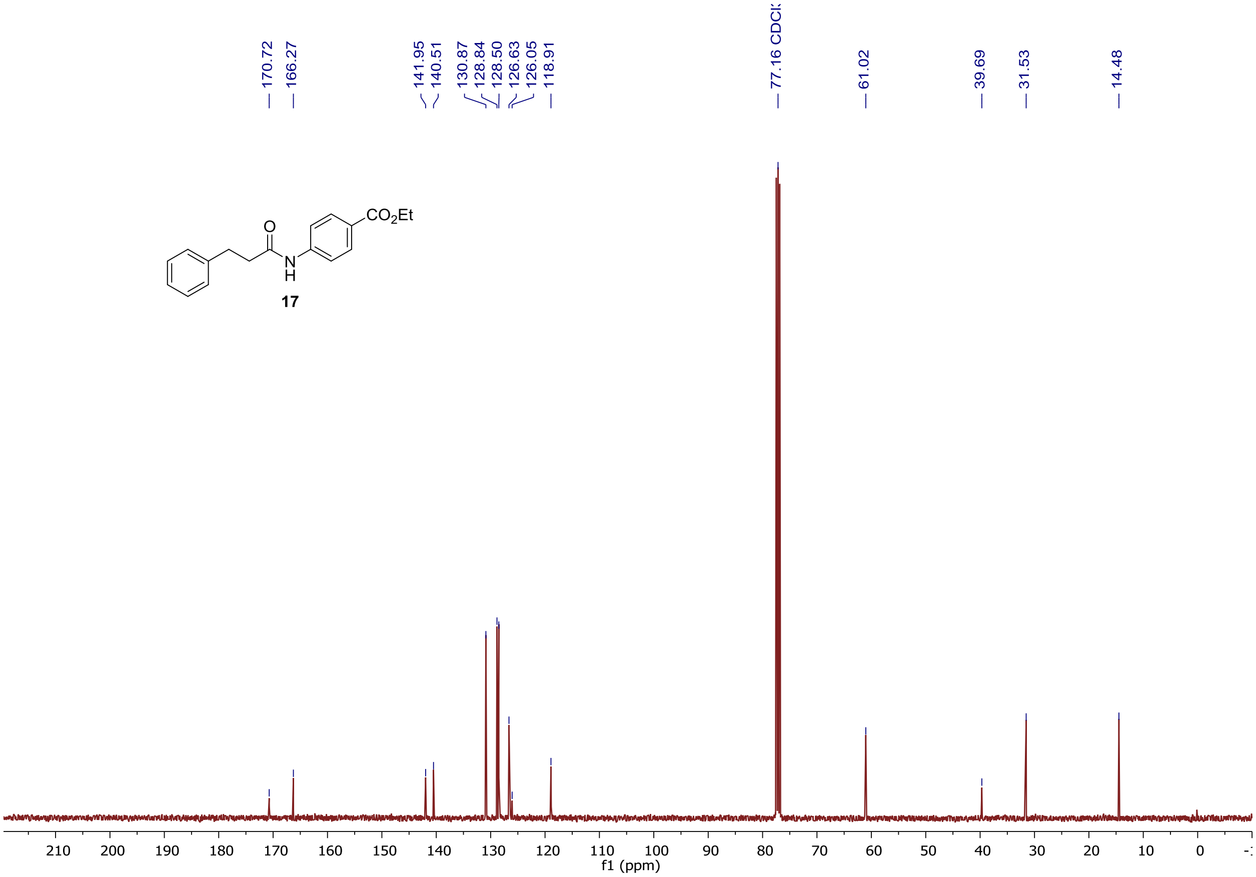
170.72
166.27
141.95
140.51
130.87
128.84
128.50
126.63
126.05
118.91
77.16 CDCl3
61.02
39.69
31.53
14.48
CO2Et
O
N
H
17
f1 (ppm)

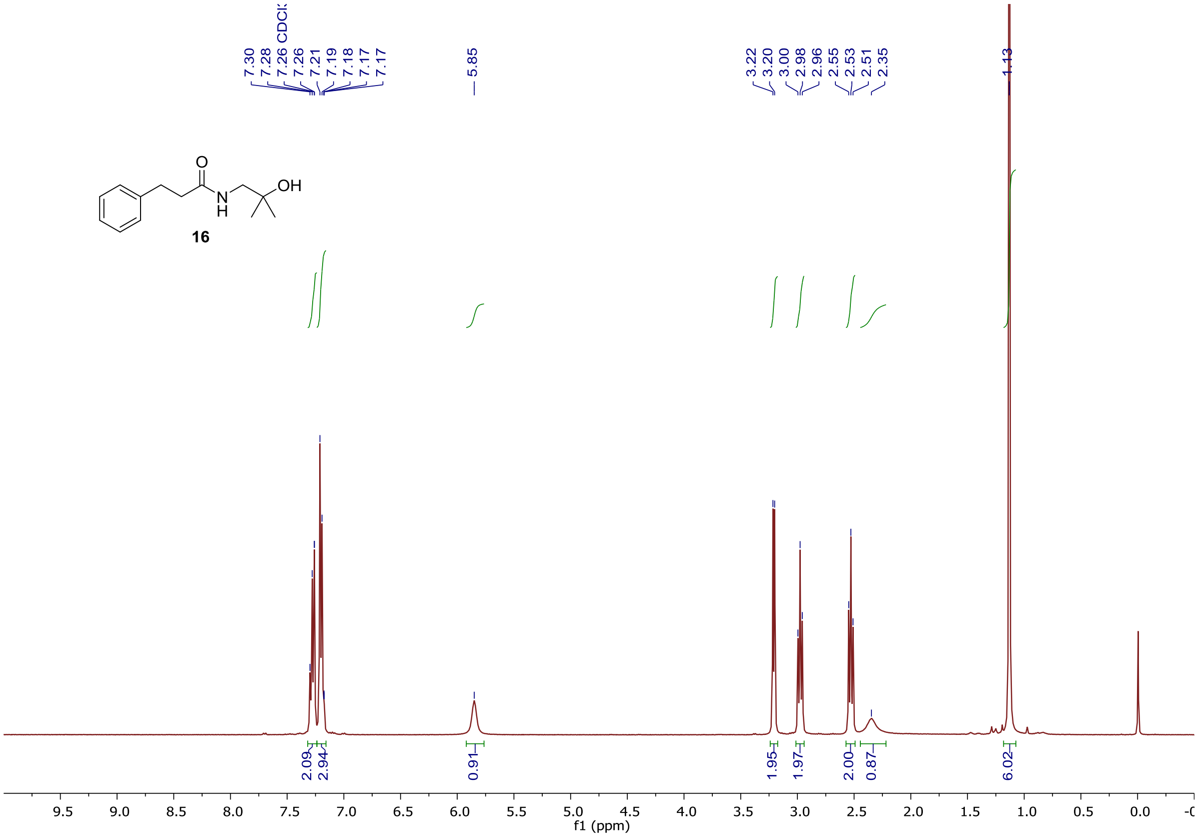
7.30
7.28
7.26 CDCl3
7.26
7.21
7.19
7.18
7.17
7.17
5.85
3.22
3.20
3.00
2.98
2.96
2.55
2.53
2.51
2.35
1.13
16
2.09
2.94
0.91
1.95
1.97
2.00
0.87
6.02
f1 (ppm)

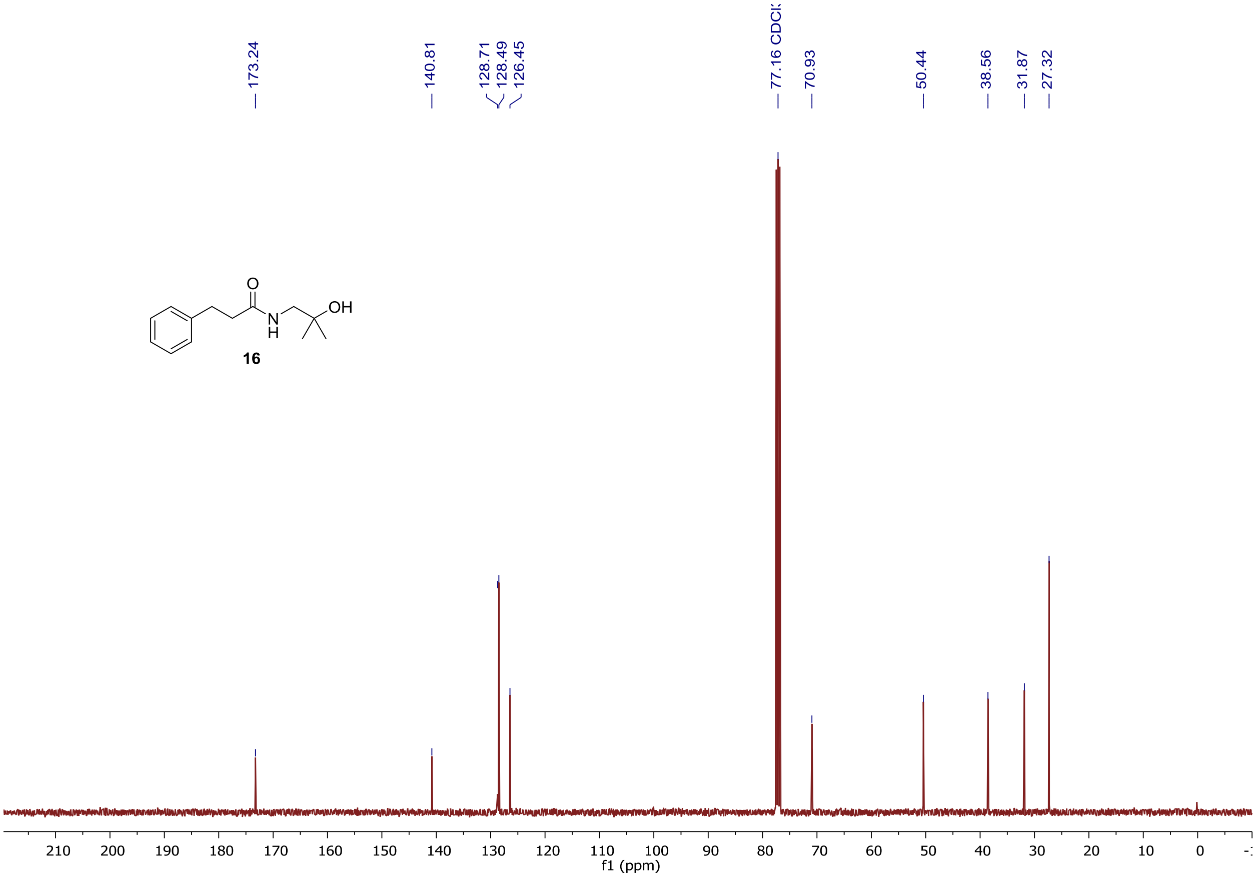
173.24
140.81
128.71
128.49
126.45
77.16 CDCl3
70.93
50.44
38.56
31.87
27.32
16
f1 (ppm)

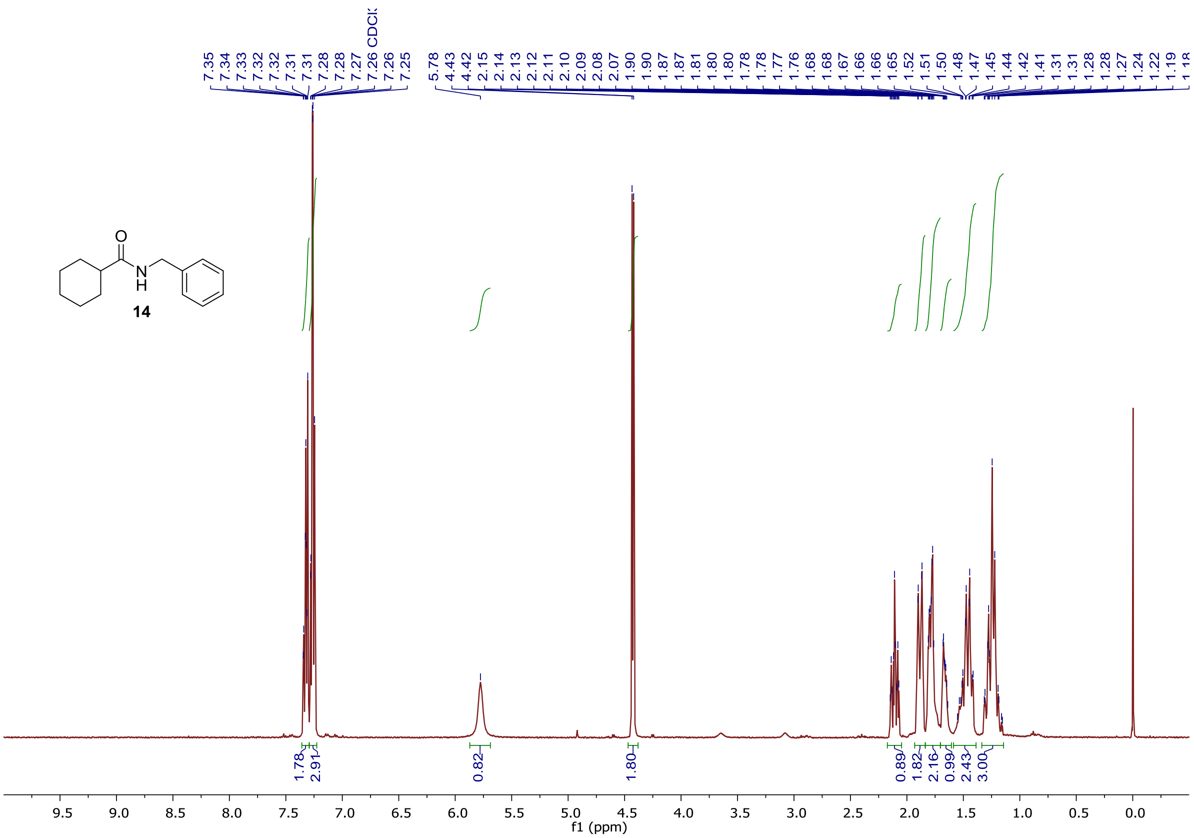

7.35
7.34
7.33
7.32
7.32
7.31
7.31
7.28
7.28
7.27
7.26 CDCl
7.26
7.25
5.78
4.43
4.42
2.15
2.14
2.13
2.12
2.11
2.10
2.09
2.08
2.07
1.90
1.90
1.87
1.87
1.81
1.80
1.80
1.78
1.78
1.77
1.76
1.68
1.68
1.67
1.66
1.66
1.65
1.52
1.51
1.50
1.48
1.47
1.45
1.44
1.42
1.41
1.31
1.28
1.28
1.27
1.24
1.22
1.19
[illegible]
O
N
H
14
1.78
2.91
0.82
1.80
0.89
1.82
2.16
0.99
2.43
3.00
9.5 9.0 8.5 8.0 7.5 7.0 6.5 6.0 5.5 5.0 4.5 4.0 3.5 3.0 2.5 2.0 1.5 1.0 0.5 0.0
f1 (ppm)


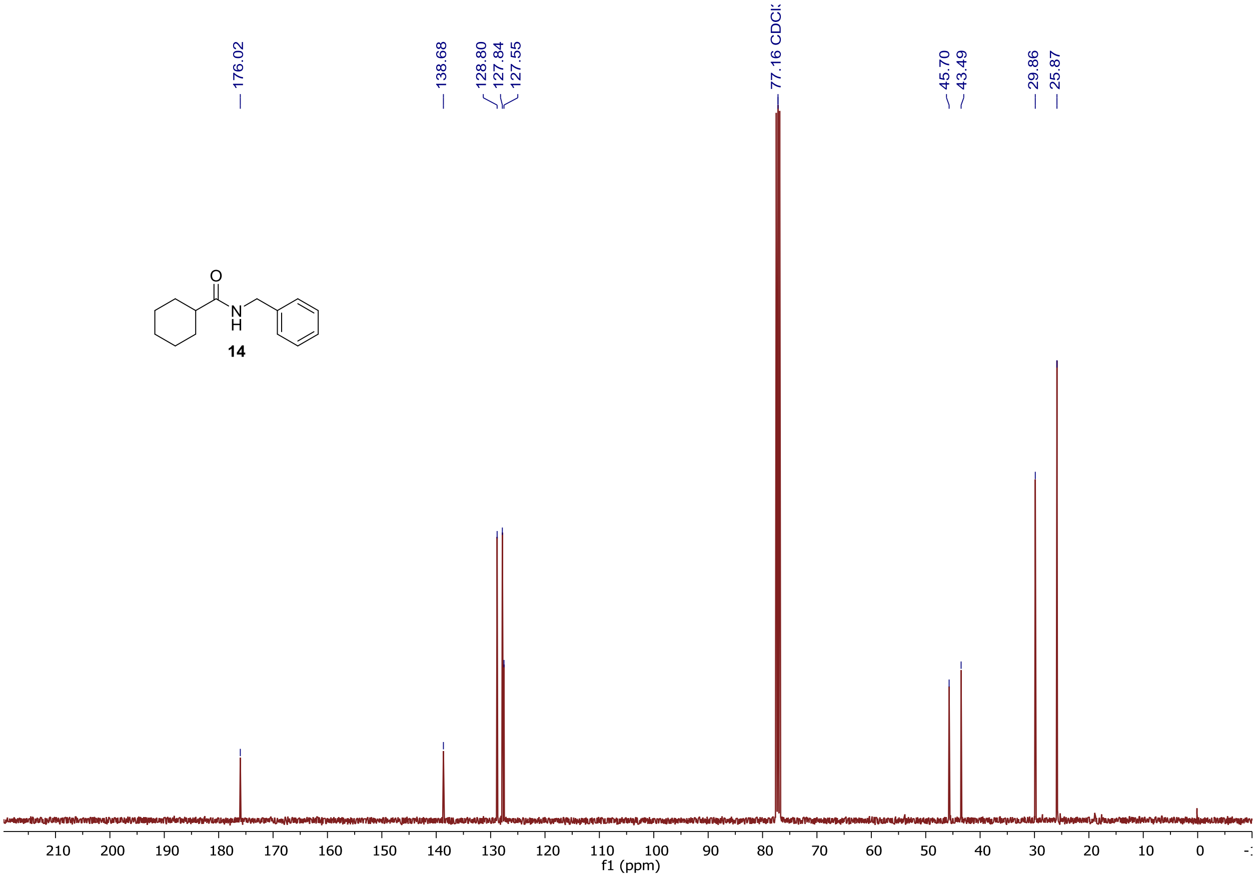

176.02
138.68
128.80
127.84
127.55
77.16 CDCl
45.70
43.49
29.86
25.87
O
N
H
14
210 200 190 180 170 160 150 140 130 120 110 100 90 80 70 60 50 40 30 20 10 0
f1 (ppm)

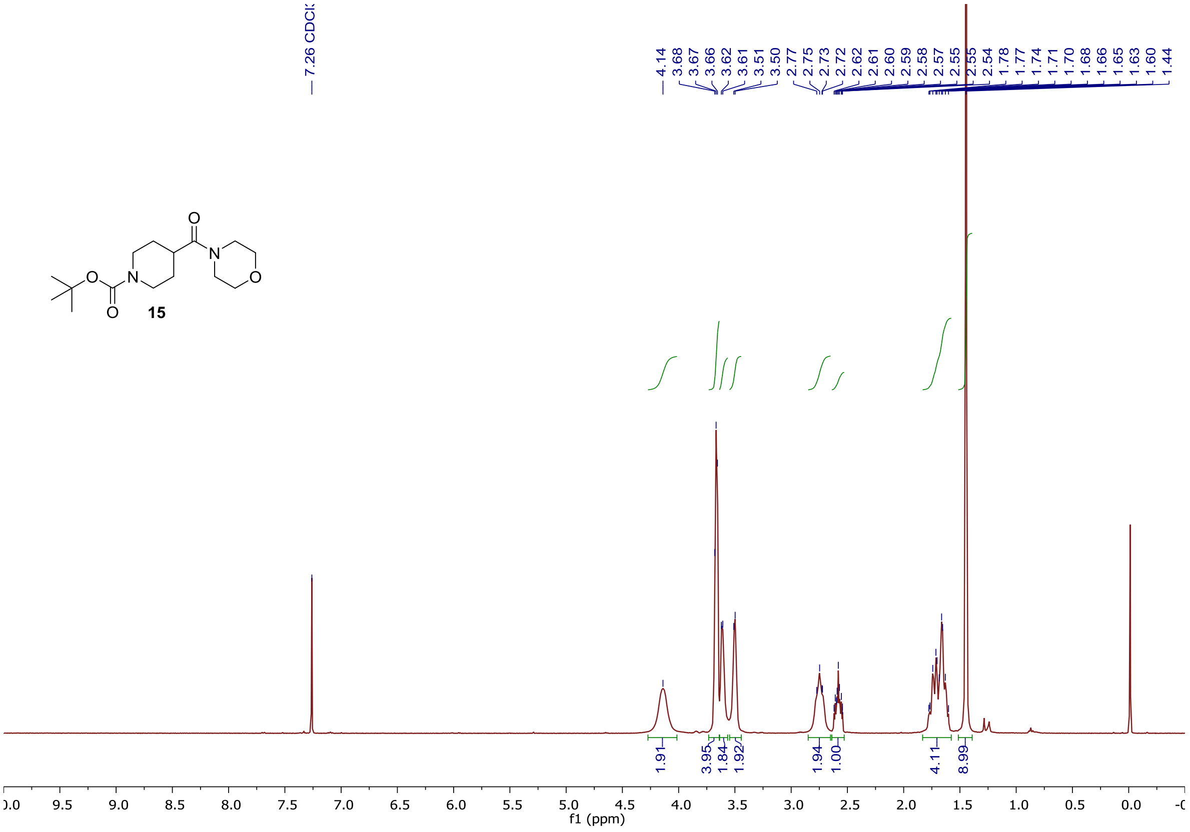
7.26 CDCl3
4.14 3.68 3.67 3.66 3.62 3.61 3.51 3.50 2.77 2.75 2.73 2.72 2.62 2.61 2.60 2.59 2.58 2.57 2.55 2.55 2.54 1.78 1.77 1.74 1.71 1.70 1.68 1.66 1.65 1.63 1.60 1.44
15
1.91 3.95 1.84 1.92 1.94 1.00 4.11 8.99
f1 (ppm)

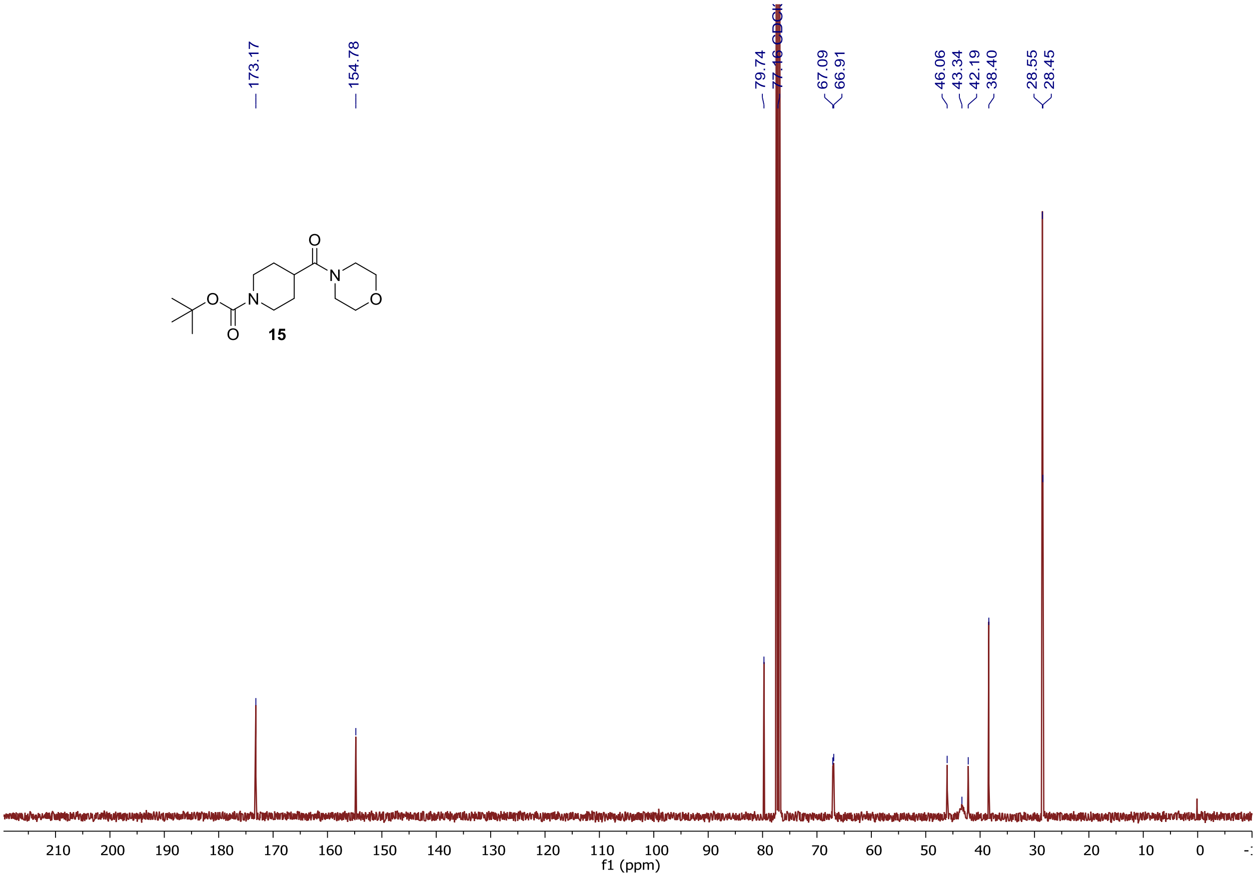
173.17
154.78
79.74
77.16 CDCl3
67.09 66.91
46.06 43.34 42.19 38.40
28.55 28.45
15
f1 (ppm)

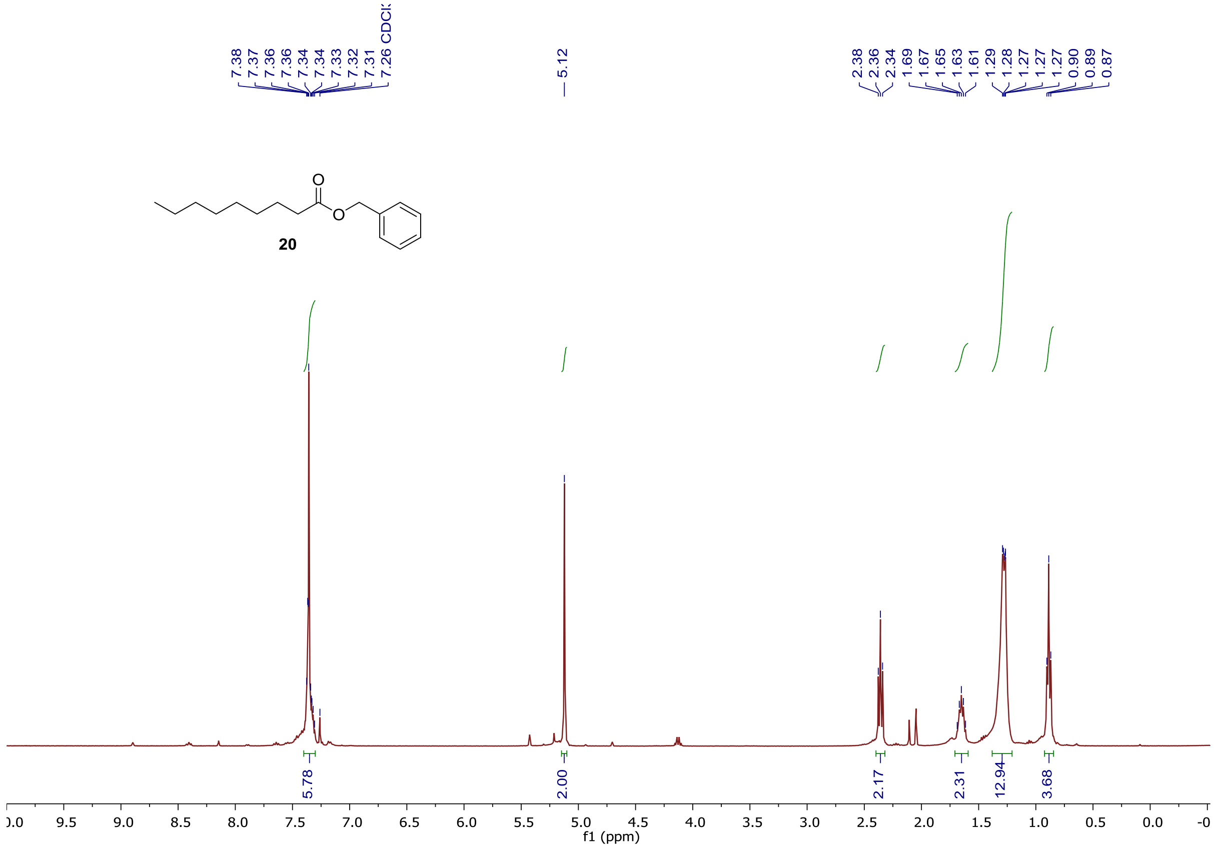
7.38
7.37
7.36
7.36
7.34
7.34
7.33
7.32
7.31
7.26 CDCl3
5.12
2.38
2.36
2.34
1.69
1.67
1.65
1.63
1.61
1.29
1.28
1.27
1.27
1.27
0.90
0.89
0.87
20
5.78
2.00
2.17
2.31
12.94
3.68
f1 (ppm)

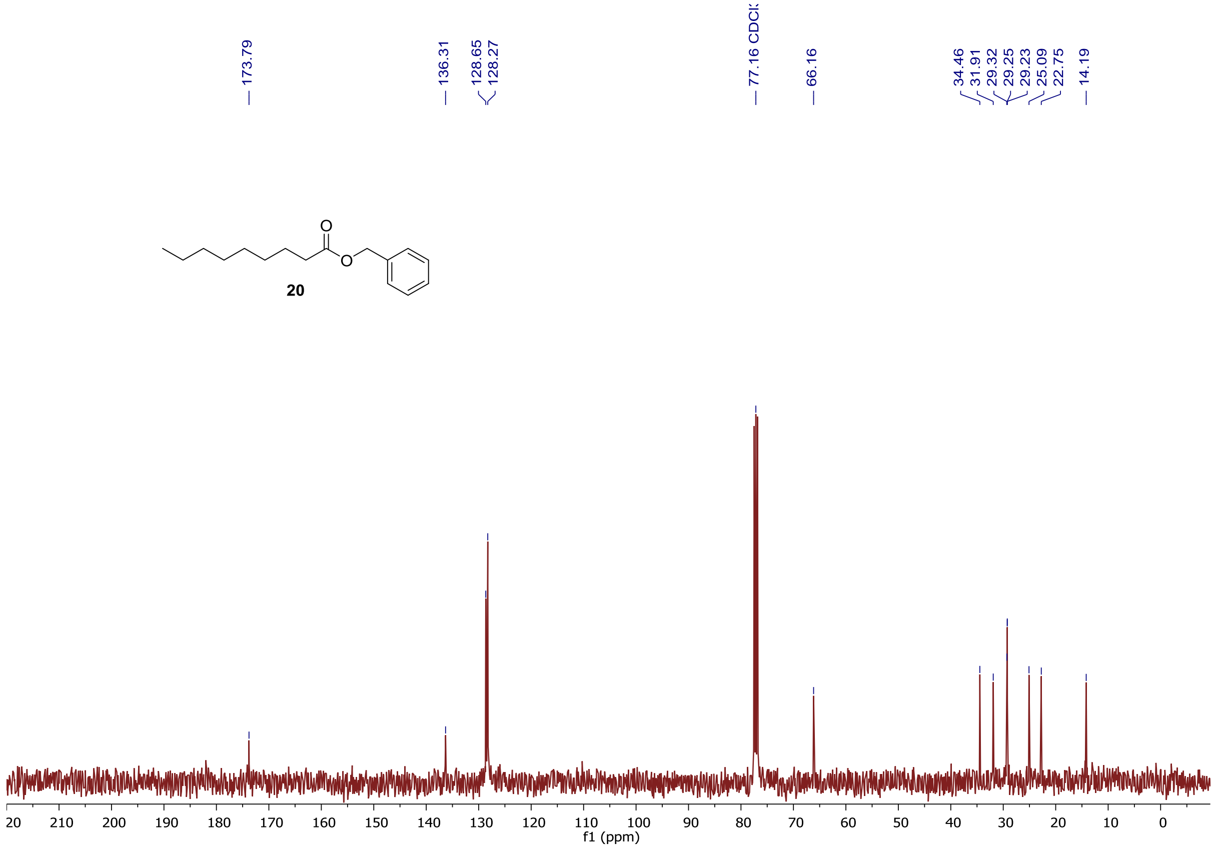
173.79
136.31
128.65
128.27
77.16 CDCl3
66.16
34.46
31.91
29.32
29.25
29.23
25.09
22.75
14.19
20
f1 (ppm)

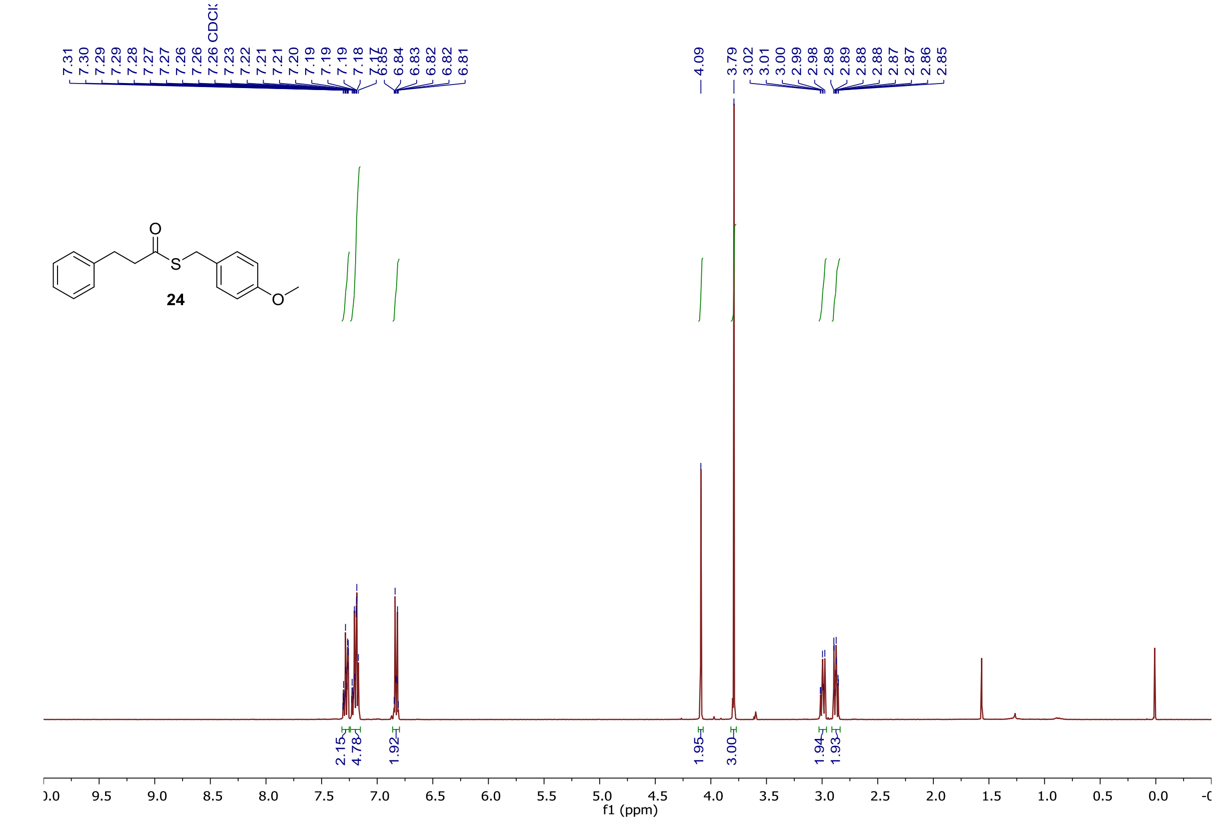
7.31
7.30
7.29
7.29
7.28
7.27
7.27
7.26
7.26
7.26 CDCl3
7.23
7.22
7.21
7.21
7.20
7.19
7.19
7.19
7.18
7.17
6.85
6.84
6.83
6.82
6.82
6.81
4.09
3.79
3.02
3.01
3.00
2.99
2.98
2.89
2.89
2.88
2.88
2.87
2.87
2.86
2.85
O
S
O
24
2.15
4.78
1.92
1.95
3.00
1.94
1.93
f1 (ppm)

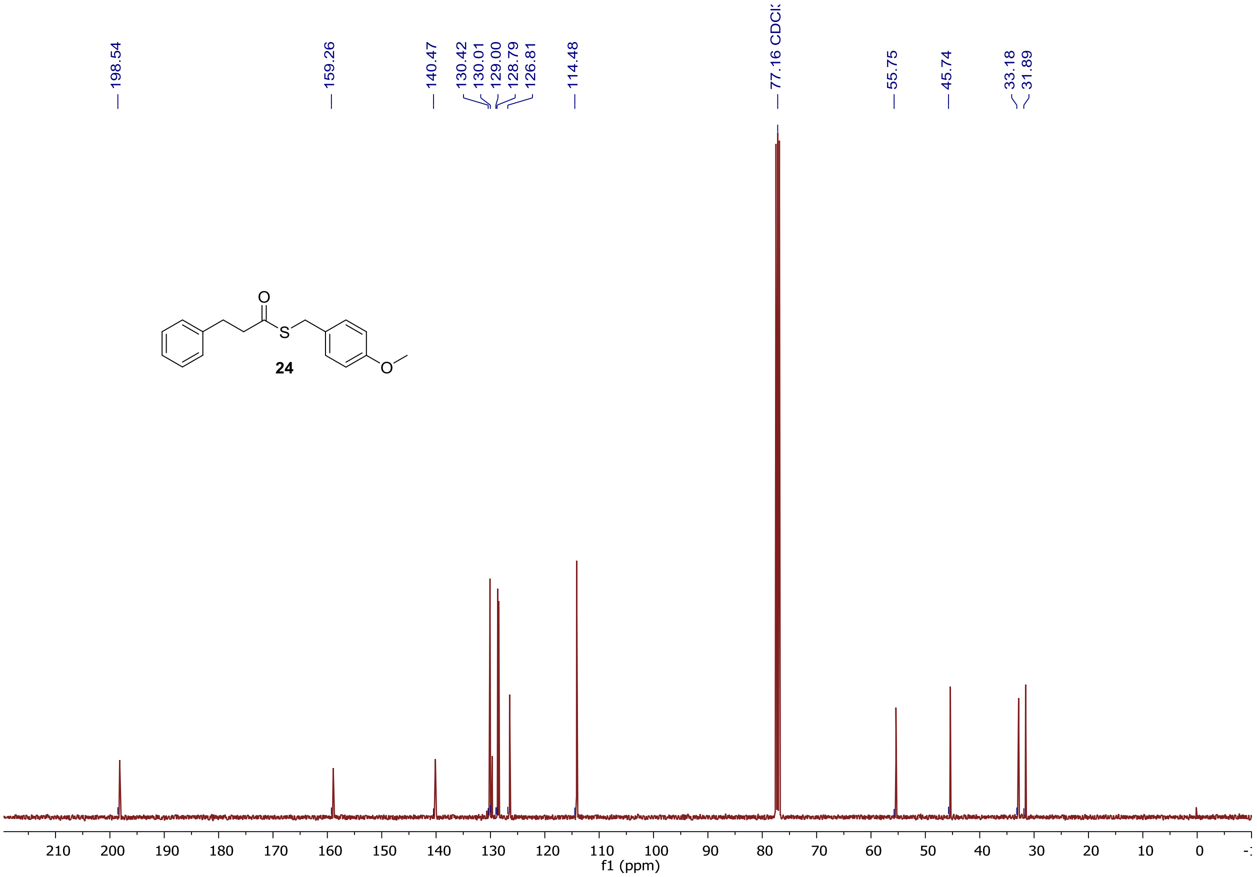
198.54
159.26
140.47
130.42
130.01
129.00
128.79
126.81
114.48
77.16 CDCl3
55.75
45.74
33.18
31.89
O
S
O
24
f1 (ppm)

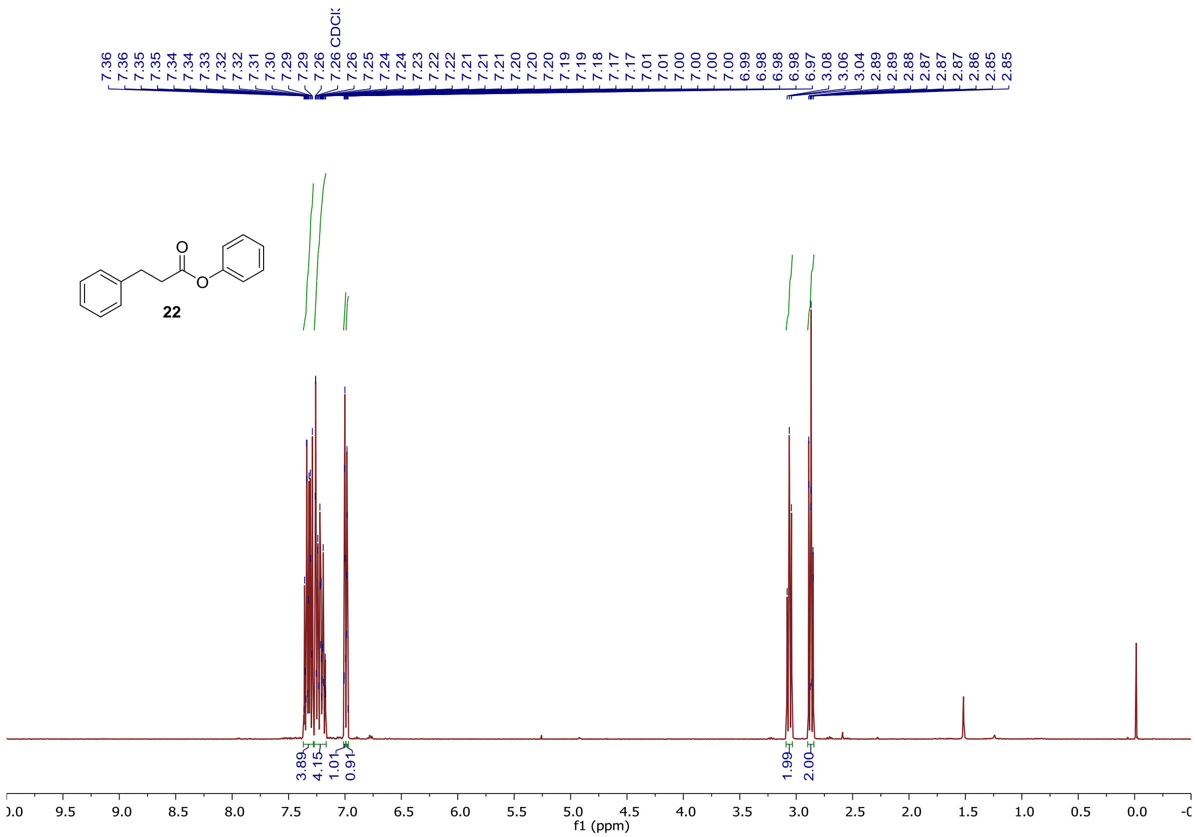
7.36
7.36
7.35
7.35
7.34
7.34
7.33
7.32
7.32
7.31
7.30
7.29
7.29
7.26 CDCl3
7.26
7.25
7.24
7.24
7.23
7.22
7.22
7.21
7.21
7.21
7.20
7.20
7.19
7.19
7.18
7.17
7.17
7.01
7.01
7.00
7.00
7.00
6.99
6.98
6.98
6.97
3.08
3.06
3.04
2.89
2.89
2.88
2.87
2.87
2.86
2.85
2.85
O
O
22
3.89
4.15
1.01
0.91
1.99
2.00
f1 (ppm)

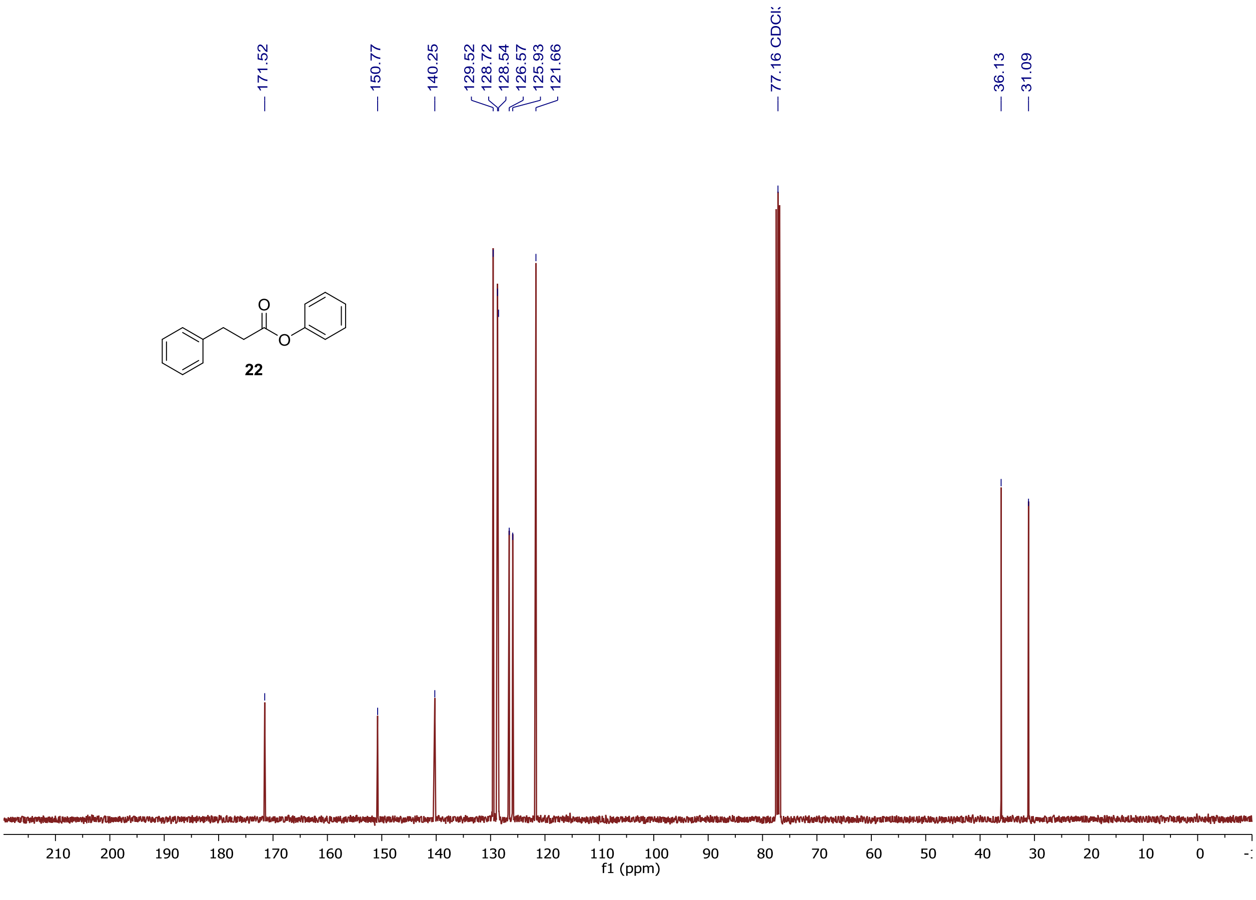
171.52
150.77
140.25
129.52
128.72
128.54
126.57
125.93
121.66
77.16 CDCl3
36.13
31.09
O
O
22
f1 (ppm)

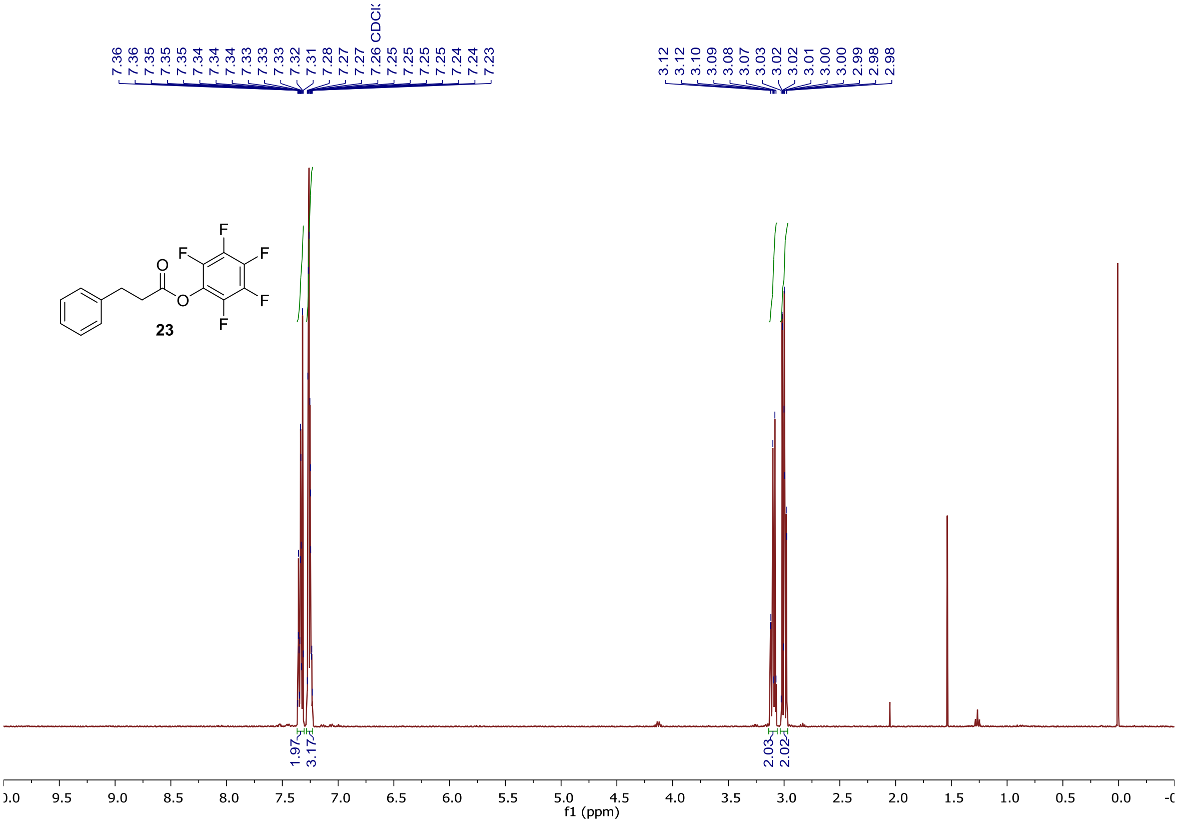

7.36
7.36
7.35
7.35
7.35
7.34
7.34
7.34
7.33
7.33
7.33
7.32
7.31
7.28
7.27
7.27
7.26 CDCl3
7.25
7.25
7.25
7.25
7.24
7.24
7.23
3.12
3.12
3.10
3.09
3.08
3.07
3.03
3.02
3.02
3.01
3.00
2.99
2.98
2.98
F
F
F
F
F
O
O
1.97
3.17
2.03
2.02
f1 (ppm)


23

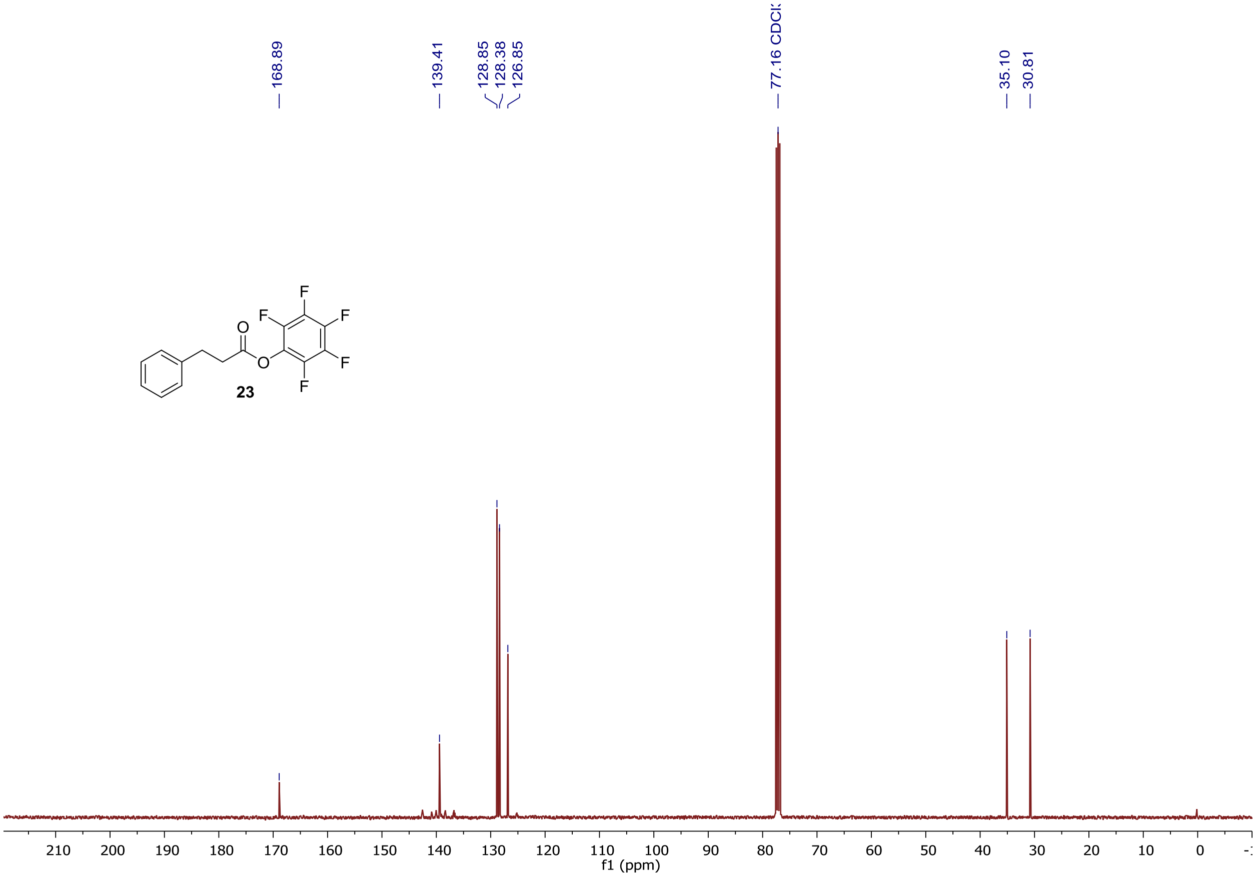

168.89
139.41
128.85
128.38
126.85
77.16 CDCl3
35.10
30.81
F
F
F
F
F
O
O
f1 (ppm)


23

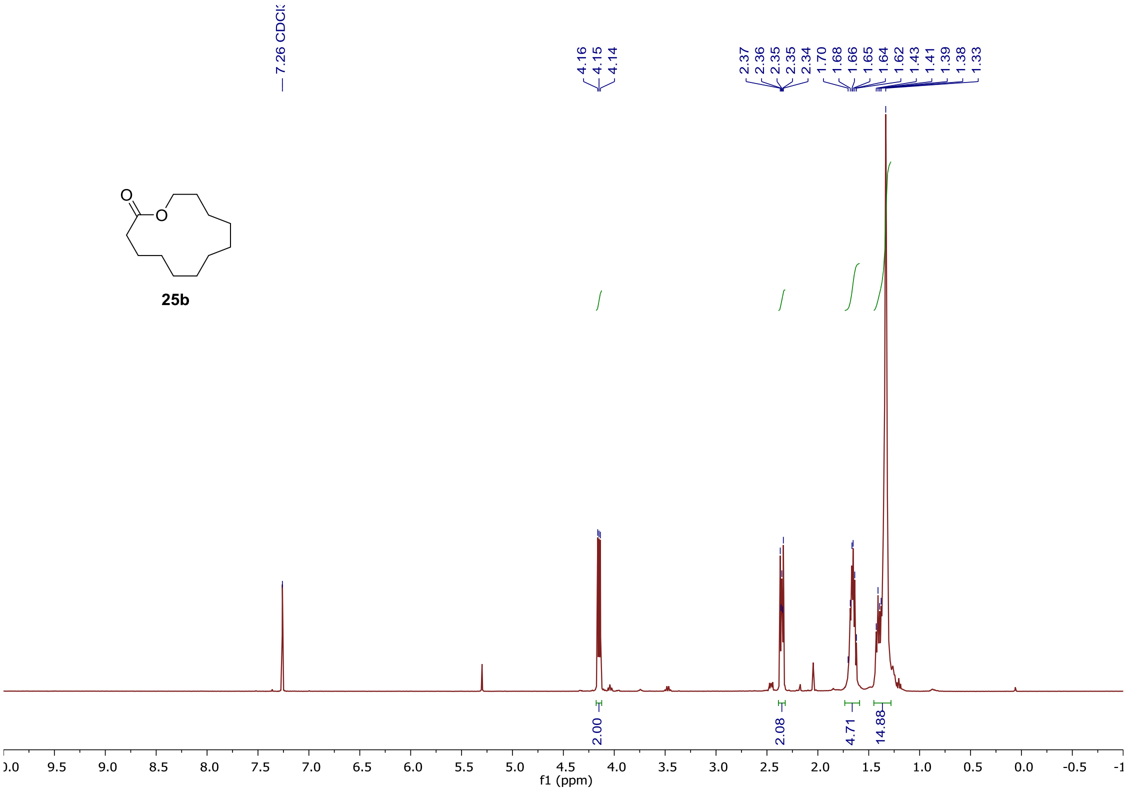
25b
7.26 CDCl3
4.16
4.15
4.14
2.37
2.36
2.35
2.35
2.34
1.70
1.68
1.66
1.65
1.64
1.62
1.43
1.41
1.39
1.38
1.33
2.00
2.08
4.71
14.88
f1 (ppm)

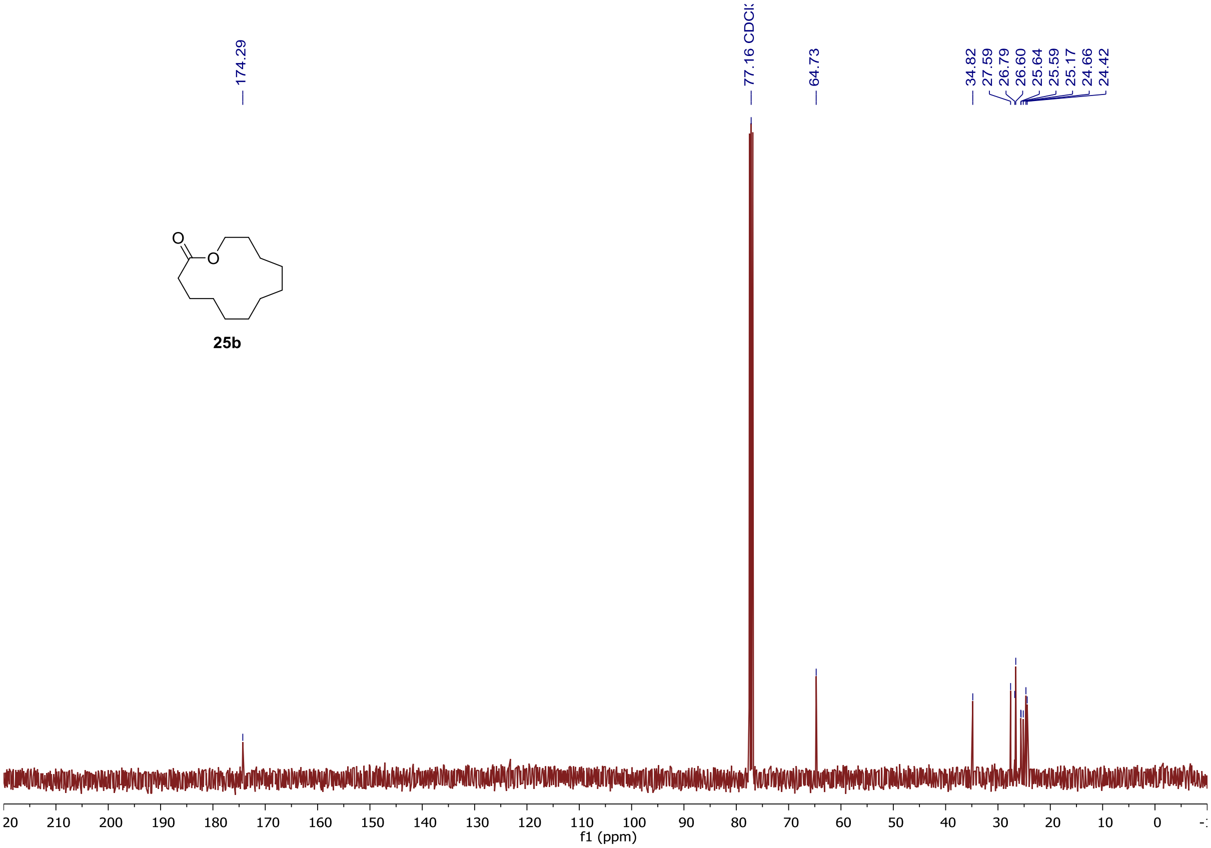
25b
174.29
77.16 CDCl3
64.73
34.82
27.59
26.79
26.60
25.64
25.59
25.17
24.66
24.42
f1 (ppm)

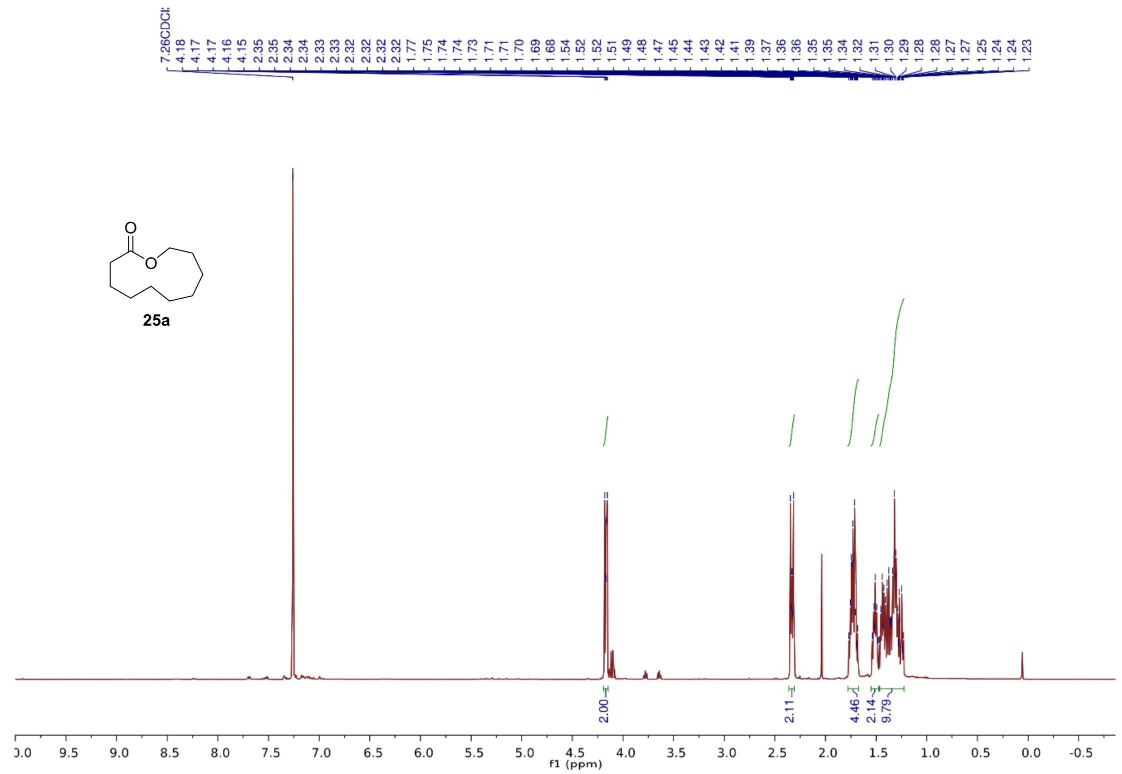
7.26CDCl3
4.18
4.17
4.17
4.16
4.15
2.35
2.35
2.34
2.34
2.33
2.33
2.32
2.32
2.32
2.32
1.77
1.75
1.74
1.74
1.73
1.71
1.71
1.70
1.69
1.68
1.54
1.52
1.52
1.51
1.49
1.48
1.47
1.45
1.44
1.43
1.42
1.41
1.39
1.37
1.36
1.36
1.35
1.35
1.34
1.32
1.31
1.30
1.29
1.28
1.28
1.27
1.27
1.25
1.24
1.24
1.23
O
O
25a
2.00
2.11
4.46
2.14
9.79
9.5 9.0 8.5 8.0 7.5 7.0 6.5 6.0 5.5 5.0 4.5 4.0 3.5 3.0 2.5 2.0 1.5 1.0 0.5 0.0 -0.5
f1 (ppm)

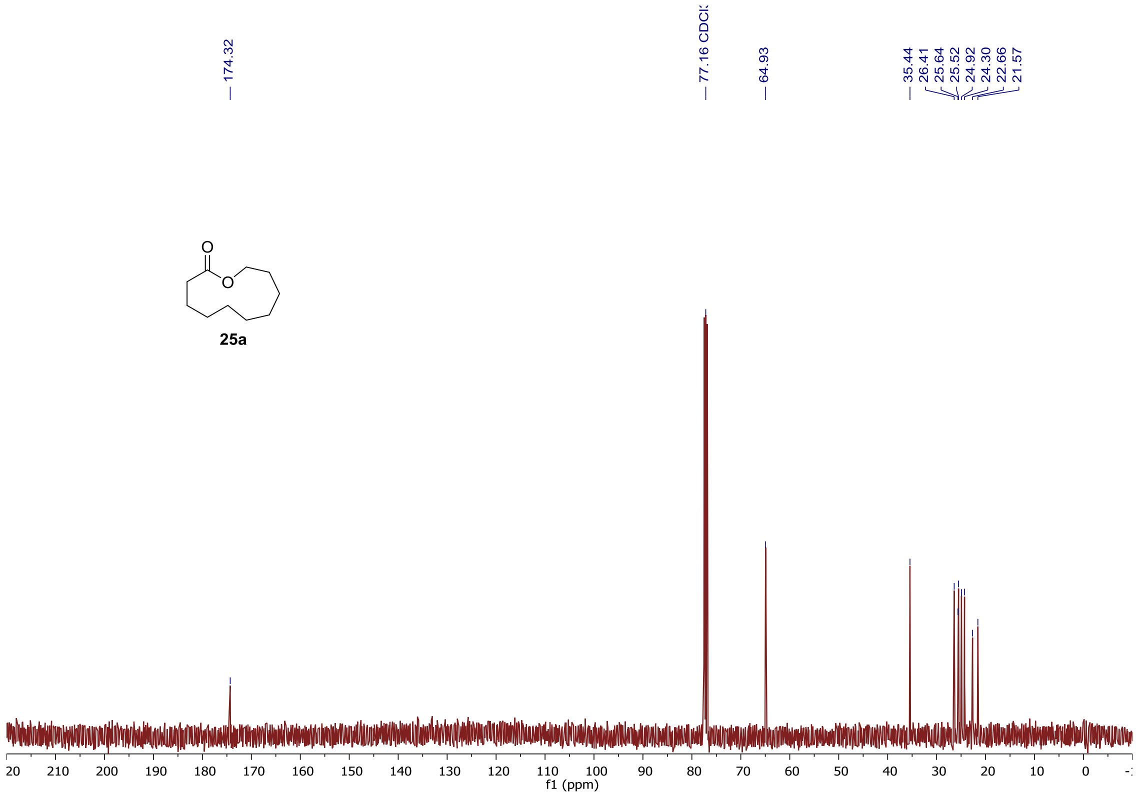
174.32
77.16 CDCl3
64.93
35.44
26.41
25.64
25.52
24.92
24.30
22.66
21.57
O
O
25a
210 200 190 180 170 160 150 140 130 120 110 100 90 80 70 60 50 40 30 20 10 0
f1 (ppm)

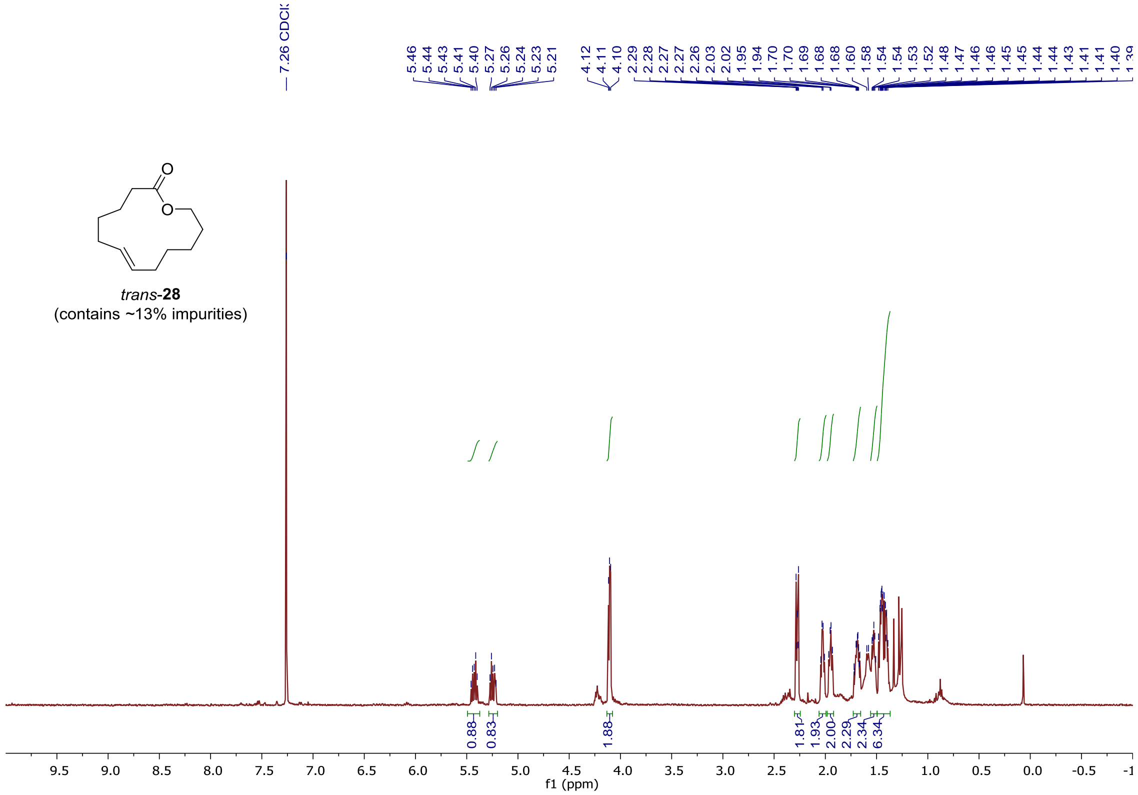
7.26 CDCl3
5.46 5.44 5.43 5.41 5.40 5.27 5.26 5.24 5.23 5.21
4.12 4.11 4.10
2.29 2.28 2.27 2.27 2.26 2.03 2.02 1.95 1.94 1.70 1.69 1.68 1.68 1.60 1.58 1.54 1.54 1.53 1.52 1.48 1.47 1.46 1.46 1.45 1.45 1.44 1.44 1.43 1.41 1.41 1.40
trans-28
(contains ~13% impurities)
0.88 0.83 1.88 1.81 1.93 2.00 2.29 2.34 6.34
f1 (ppm)

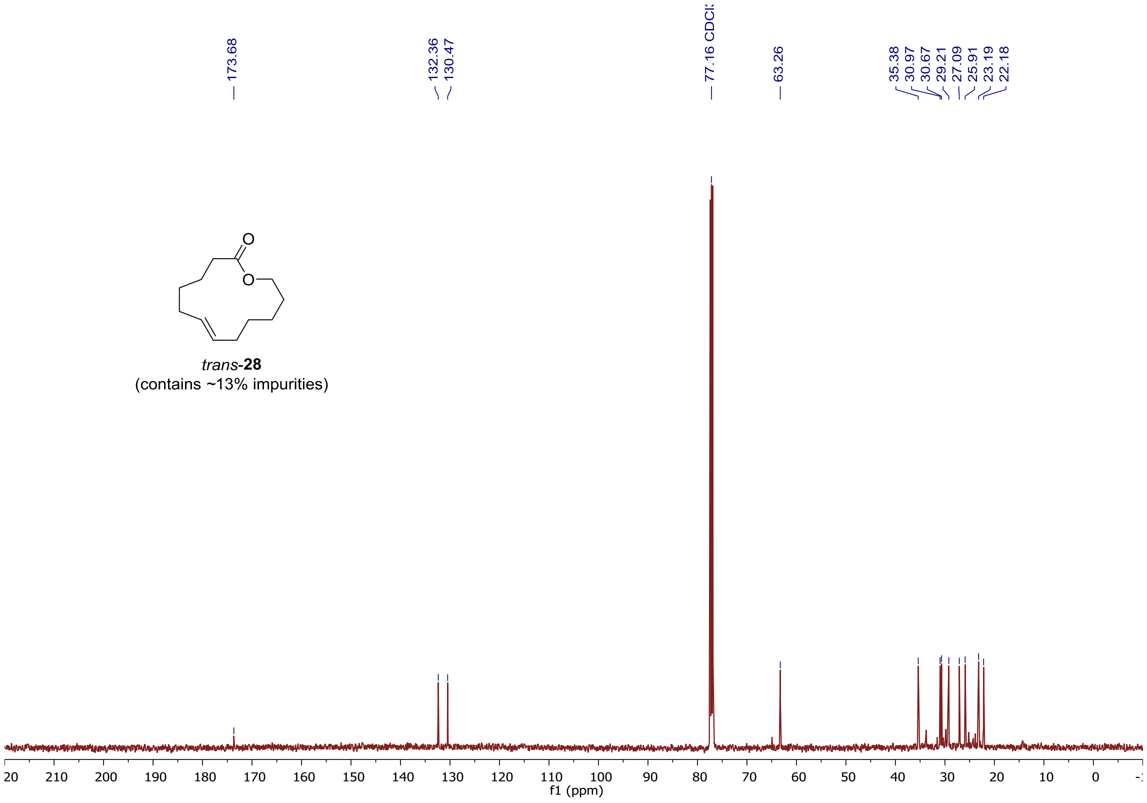
173.68
132.36
130.47
77.16 CDCl3
63.26
35.38 30.97 30.67 29.21 27.09 25.91 23.19 22.18
trans-28
(contains ~13% impurities)
f1 (ppm)

## 5. References


1. Kim, M.-J.; Kim, J.-J.; Won, J.-E.; Kang, S.-B.; Park, S.-E.; Jung, K.-J.; Lee, S.-G.; Yoon, Y.-J. Lactonization of *ω*-Hydroxycarboxylic Acids Using (4,5-Dichloro-6-oxo-6*H*-pyridazin-1-yl)phosphoric Acid Diethyl Ester. *Bull. Korean Chem. Soc.* **2008**, *29*, 2247–2251. https://doi.org/10.5012/bkcs.2008.29.11.2247.
2. Shen, L.-L.; Mun, H.-S.; Jeong, J.-H. Macrocyclisation of Macrodiolide with Dimethylaluminium Methaneselenolate. *Eur. J. Org. Chem.* **2010**, *2010*, 6895–6899. https://doi.org/10.1002/ejoc.201001201.
3. Cha, J. K.; Kulinkovich, O. G. The Kulinkovich Cyclopropanation of Carboxylic Acid Derivatives. *Org. React*. **2012**, *77*, 1–160. https://doi.org/10.1002/0471264180.or077.01.
4. Elek, G. Z.; Borovkov, V.; Lopp, M.; Kananovich, D. G. Enantioselective One-Pot Synthesis of *α*,*β*-Epoxy Ketones via Aerobic Oxidation of Cyclopropanols. *Org. Lett.* **2017**, *19*, 3544–3547. https://doi.org/10.1021/acs.orglett.7b01519.
5. Nomura, K.; Matsubara, S. Stereospecific Construction of Chiral Tertiary and Quaternary Carbon by Nucleophilic Cyclopropanation with Bis(iodozincio)methane. *Chem. Asian J.* **2010**, *5*, 147–152. https://doi.org/10.1002/asia.200900289.
6. Elek, G. Z.; Koppel, K.; Zubrytski, D. M.; Konrad, N.; Järving, I.; Lopp, M.; Kananovich, D. G. Divergent Access to Histone Deacetylase Inhibitory Cyclopeptides via a Late-Stage Cyclopropane Ring Cleavage Strategy. Short Synthesis of Chlamydocin. *Org. Lett.* **2019**, *21*, 8473–8478. https://doi.org/10.1021/acs.orglett.9b03305.
7. Zubrytski, D. M.; Kananovich, D. G.; Kulinkovich, O. G. A highly stereoselective route to medium-ring-sized trans-alkenolides via oxidative fragmentation of bicyclic oxycyclopropane precursors: application to the synthesis of (+)-recifeiolide. *Tetrahedron* **2014**, *70*, 2944–2950. https://doi.org/10.1016/j.tet.2014.03.032.
8. Taber, D. F.; Qiu, J. Permaleic Acid: Baeyer–Villiger Oxidation of Cyclododecanone. *J. Chem. Educ.* **2013**, *90*, 1103–1104. https://doi.org/10.1021/ed400083m.
